# Supplementary material for: The caspase-activated DNase promotes cellular senescence
Source: EMBO J. 2024 Jul 8;43(16):11. doi: 10.1038/s44318-024-00163-9 (PMC11329656; doi:10.1038/s44318-024-00163-9)
Supplement: Supplementary file 1 — Appendix [file 44318_2024_163_MOESM1_ESM.pdf]

Appendix for

The caspase-activated DNase promotes cellular senescence

Aladin Haimovici[1], Valentin Rupp[1], Tarek Amer[1], Abdul Moeed[1], Arnim Weber[1] and Georg Häcker[1, 2*],

* Corresponding author. Email: georg.haecker@uniklinik-freiburg.de
1 Institute of Medical Microbiology and Hygiene, Medical Center, University of Freiburg, Faculty of Medicine, Freiburg, Germany

2 BIOSS Centre for Biological Signalling Studies, University of Freiburg, Germany

Table of contents **Page**

- Appendix Figure S1 to S4 2-5
- Appendix Table S1 6

Appendix Figure S1

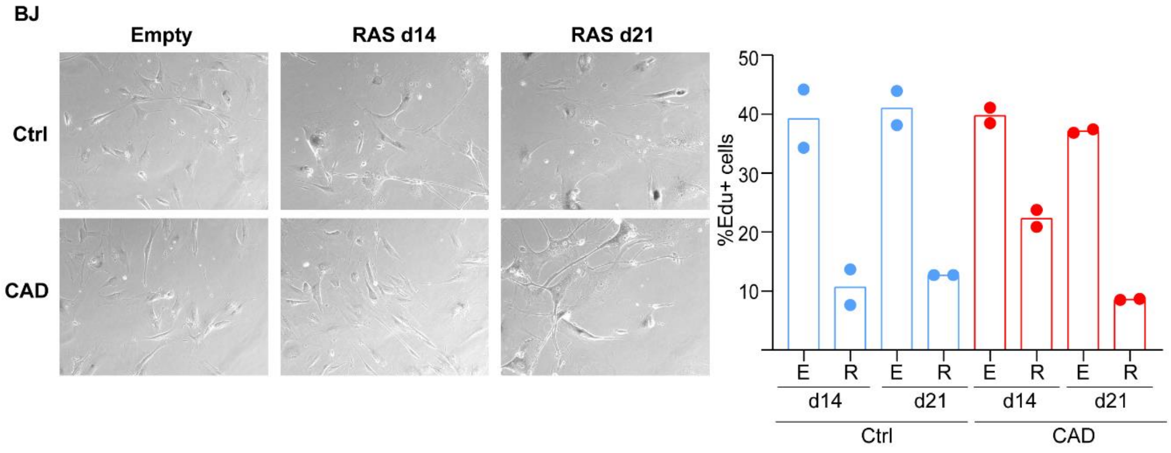

Appendix Figure S1

BJ fibroblasts (carrying a non-targeting gRNA (Ctrl) or CAD-deficient) were transduced with empty retrovirus or pLNCX2-ER:H-RAS^{G12V}-virus and stimulated each day with tamoxifen to activate H-RAS^{G12V}. Brightfield pictures were taken 14 and 21 days post-transduction. Proliferation was measured by Edu incorporation 14 and 21 days after virus transduction. Percentage of Edu^{+} cells is shown. Each symbol represent one experiment.

Appendix Figure S2

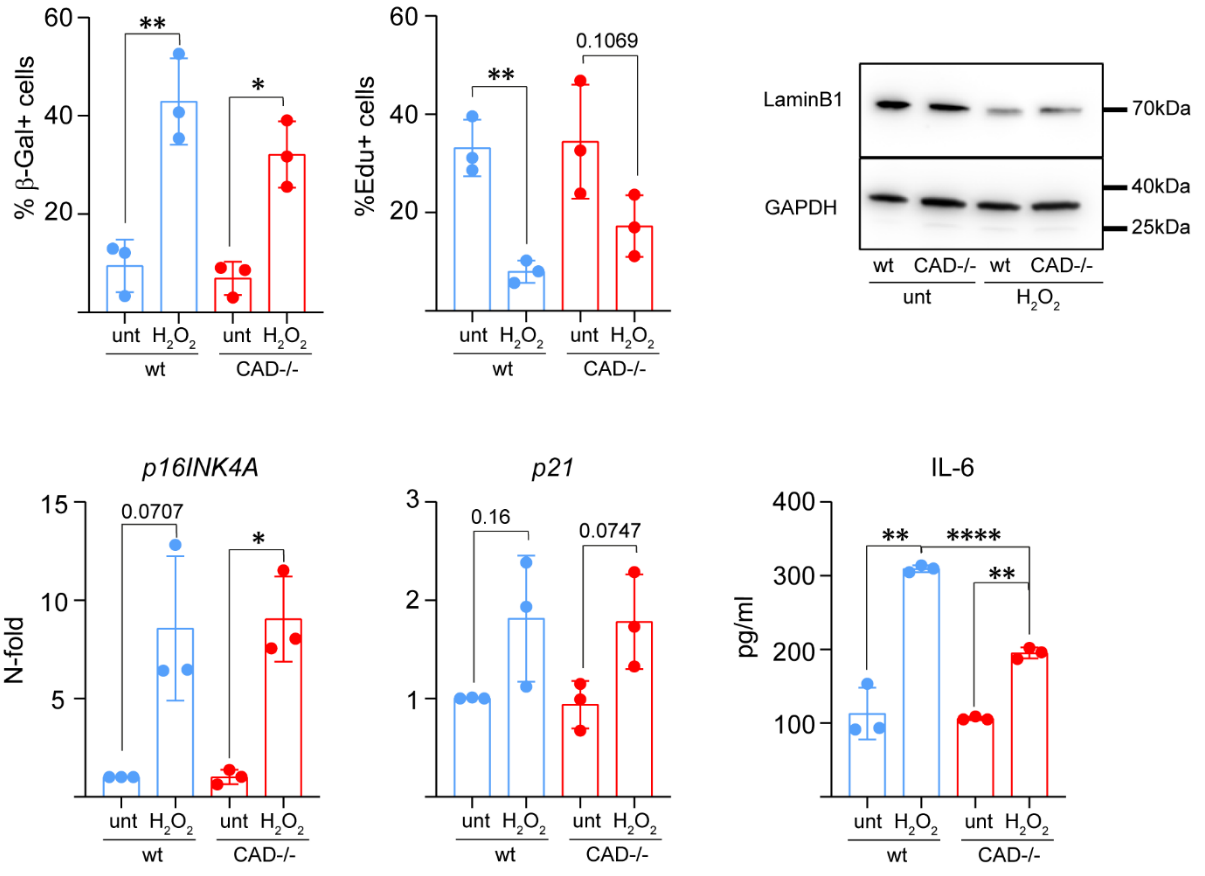

Appendix Figure S2

Wt and CAD-deficient MEFs were treated with 500 µM H_2O_2 in serum-free medium for 1 h. Medium was replaced, and cells were incubated for 7 days. Senescence profile was assessed by measurement of β-Galactosidase activity, proliferation (Edu), LaminB1 protein (Western blot), mRNA expression of senescence-associated gene (RT-PCR) and IL-6 protein (ELISA). Data are the mean/SD of 3 independent experiments. Each symbol represents one experiment. *$p < 0.05$, **$p < 0.01$, ****$p < 0.0001$.

Appendix Figure S3

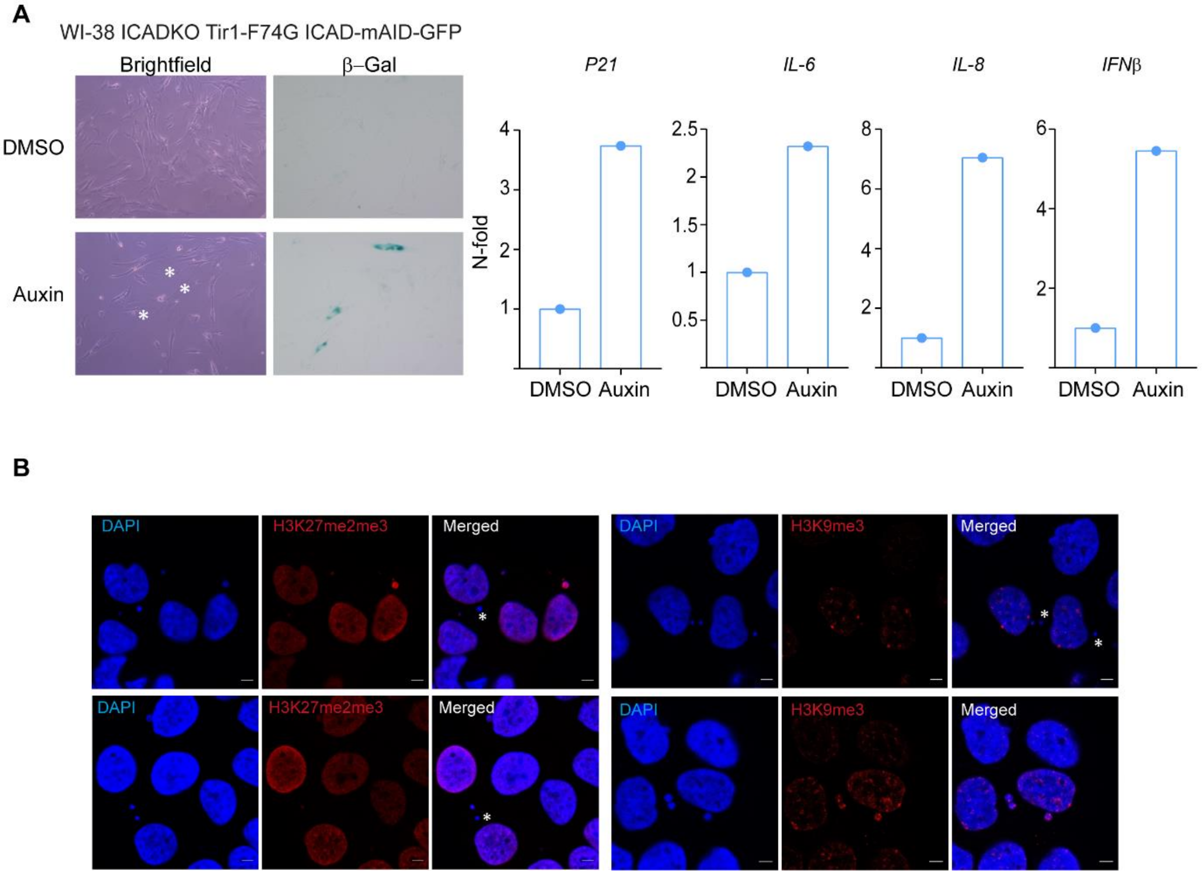

Appendix Figure S3

A, WI-38-ICAD-TIR1-F74G-T2A-mICAD-mAID-GFP cells were treated with auxin for 24 h, media was replaced and cells incubated for 24h. Auxin treatment was repeated two more times (with 24h recovery in normal media in between treatment) and cells were photographed and stained for β-Galactosidase. Brightfield images (left panel) show cells with flat and enlarge morphology (white stars) and right panel show cells positive for β-Galactosidase. Expression of senescence-associated genes was measured by RT-PCR. Symbols show results from one independent experiment. **B**, HaCaT-ICAD-mAID-GFP cells were treated with auxin for 6h. Media was then replaced and cells were incubated for 24h in normal media. Cells were fixed and stained for H3K9me3 or H3K27me2me3. Nucleus were stained with DAPI. Slides were imaged with confocal microscopy. Scale bar: 10µM.

Appendix Figure S4

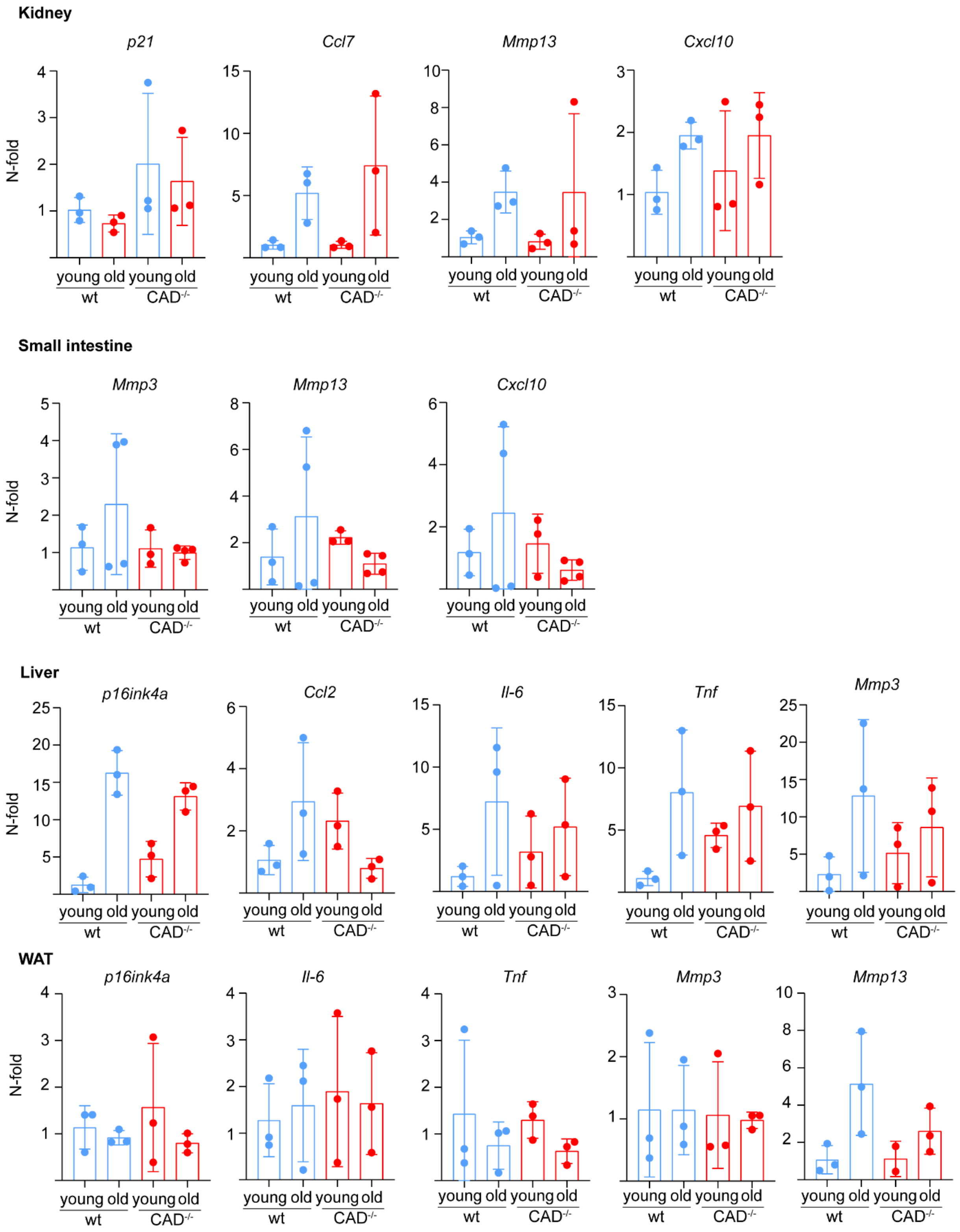

Appendix Figure S4

Expression of senescence-associated genes in kidney, small intestine, WAT and liver samples. Gene expression was measured by RT-PCR. Young (8-10 weeks) (n=3) and old (75 weeks) (n=3-4) animals were analyzed. Each symbol represents one mouse.

Appendix Table S1

Target	Species	Sequence forward 5‘-3‘	Sequence reverse 5’-3’
Gapdh	Mouse	GTCATCCCAGAGCTGAACG	TCATACTTGGCAGGTTTCTCC
Cdkn2a	Mouse	GATTCAGGTGATGATGATGGGC	TGCACCGTAGTTGAGCAGAAG
p21	Mouse	CTGGTGATGTCCGACCTGTT	TCAAAGTTCCACCGTTCTCG
Ccl2	Mouse	CCTGCTGCTACTCATTCACCA	ATTCCTTCTTGGGGTCAGCA
Ccl7	Mouse	GAGGATCTCTGCCACGCTTC	ACACCGACTACTGGTGATCC
Il-6	Mouse	TAGTCCTTCCTACCCCAATTTCC	TTGGTCCTTAGCCACTCCTTC
Tnf	Mouse	CAGGCGGTGCCTATGTCTC	CGATCACCCCGAAGTTCAGTAG
Mmp3	Mouse	CCTGATGTTGGTGGCTTCA	TCCTGTAGGTGATGTGGGATTTC
Mmp13	Mouse	ACTTCTACCCATTTGATGGACCTT	AAGCTCATGGGCAGCAACA
GAPDH	Human	GAGTCAACGGATTTGGTCGT	GACAAGCTTCCCGTTCTCAG
P21	Human	TCACTGTCTTGTACCCTTGTGC	GGCGTTTGGAGTGGTAGAAA
TNF	Human	CTGCCCCAATCCCTTTATT	CCCAATCTCTTTTTGAGCC
IL-8	Human	ACTGAGAGTGATTGAGAGTC	AACCCTCTGCACCCAGTTTTC
MMP1	Human	ATGCACAGCTTTCCTCCACT	GTTGTCCCGATGATCTCCCC
CXCL10	Human	AGTGGCATTCAAGGAGTACC	TGATGGCCTTCGATTCTGGATACC
IFNβ	Human	ATGACCAACAAGTGTCTCCTCC	GGAATCCAAGCAAGTTGTAGCTC
