# Supplementary figures and images for: The caspase-activated DNase promotes cellular senescence
Source: EMBO J. 2024 Jul 8;43(16):11. doi: 10.1038/s44318-024-00163-9 (PMC11329656; doi:10.1038/s44318-024-00163-9)

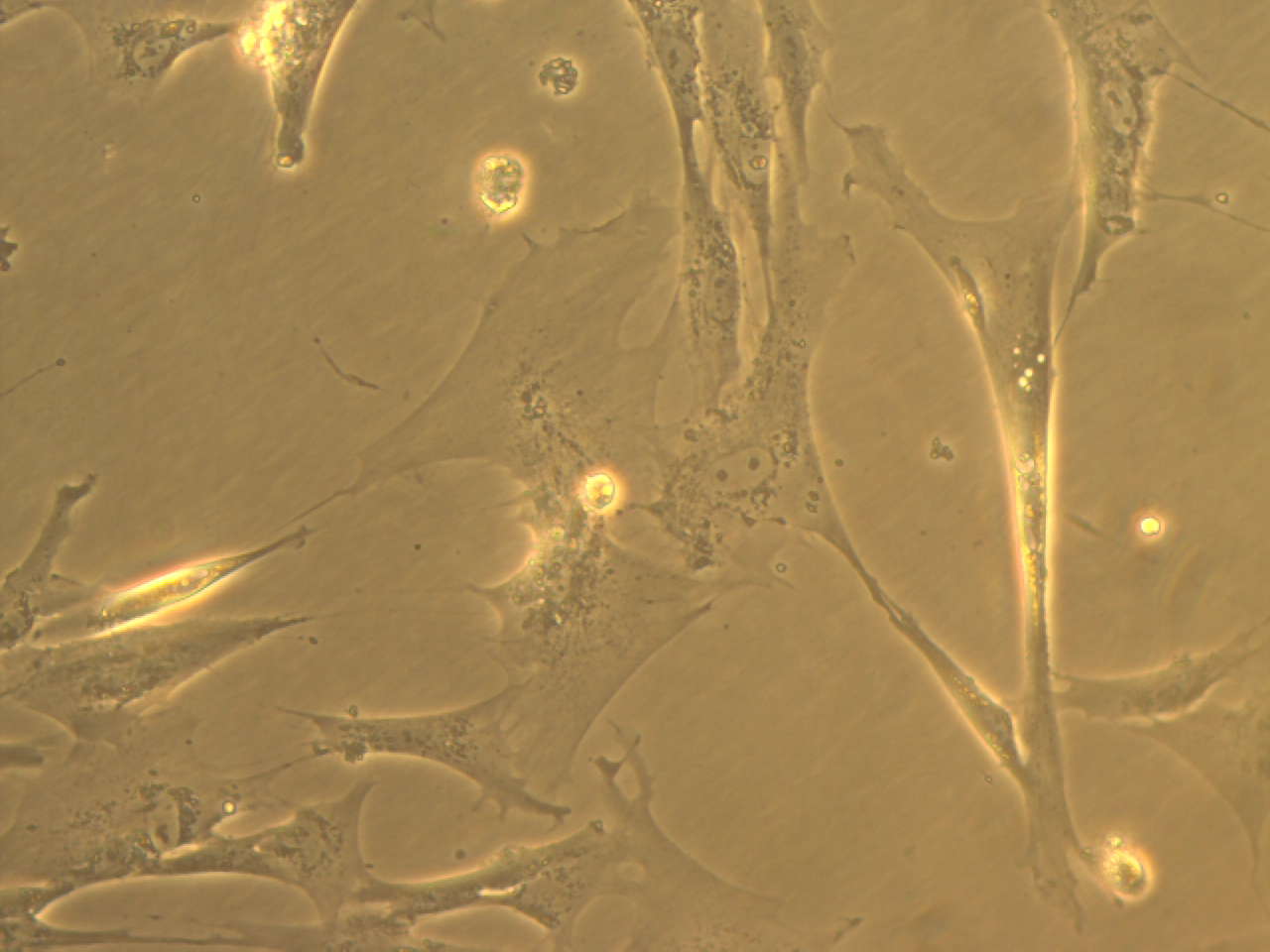

Supplement: Supplementary file 3 — Source data Fig. 1 [file 44318_2024_163_MOESM3_ESM.zip › Figure 1/1A/Light microscopy pic WI38 ABT.tif]

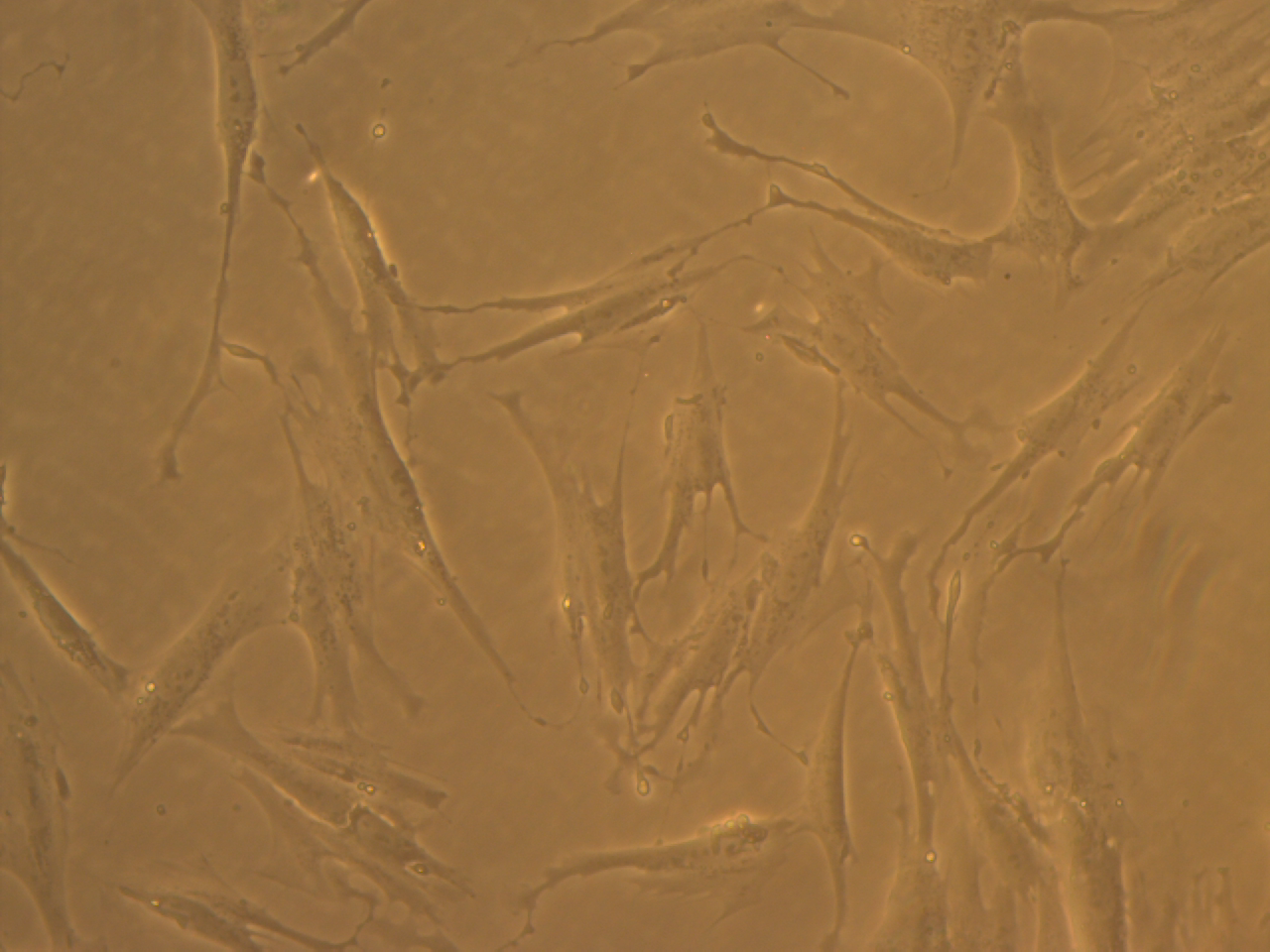

Supplement: Supplementary file 3 — Source data Fig. 1 [file 44318_2024_163_MOESM3_ESM.zip › Figure 1/1A/Light microscopy pic WI38 DMSO.tif]

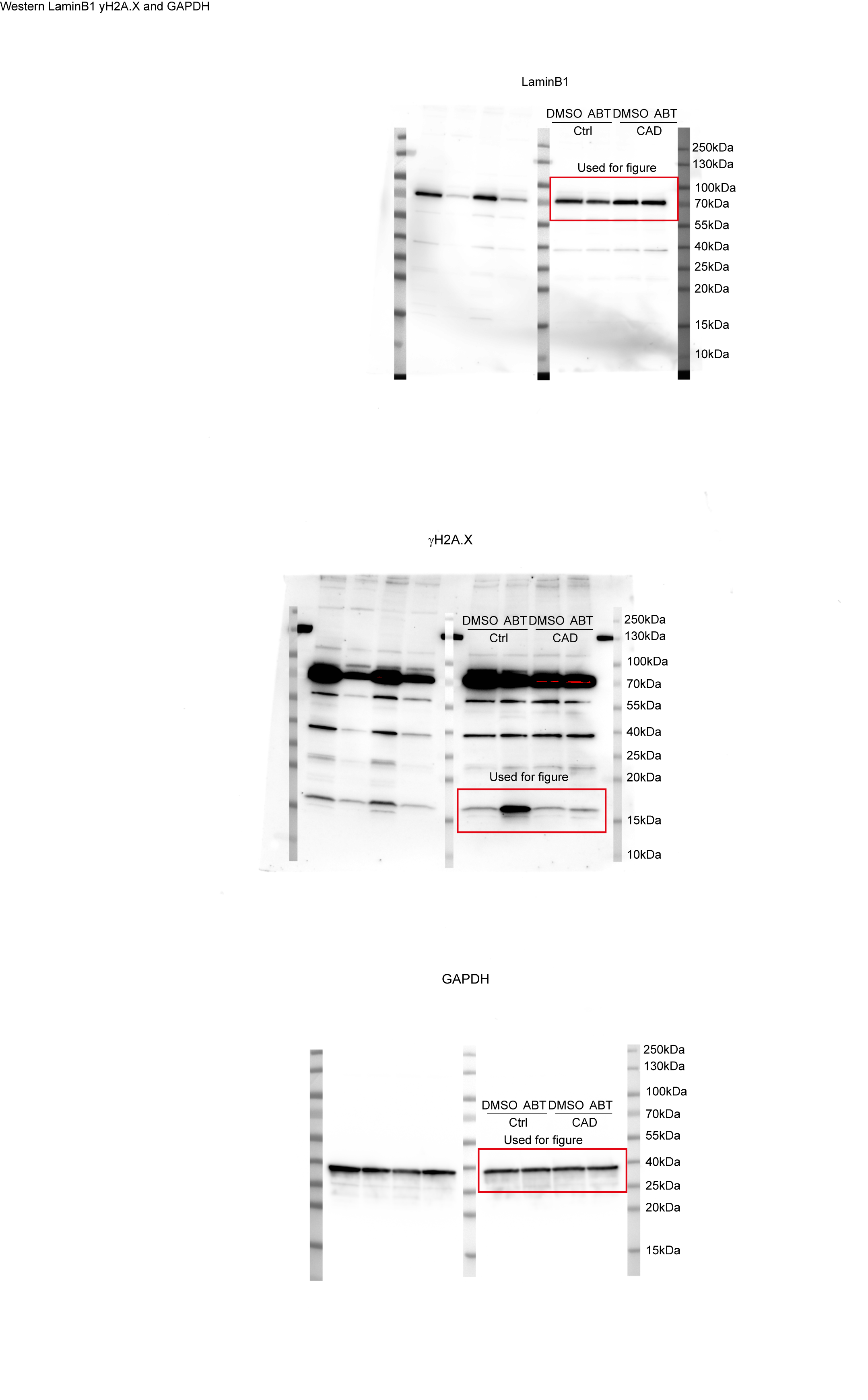

Supplement: Supplementary file 3 — Source data Fig. 1 [file 44318_2024_163_MOESM3_ESM.zip › Figure 1/1B/Western yH2AX LaminB1 GAPDH WI38 ABT.tif]

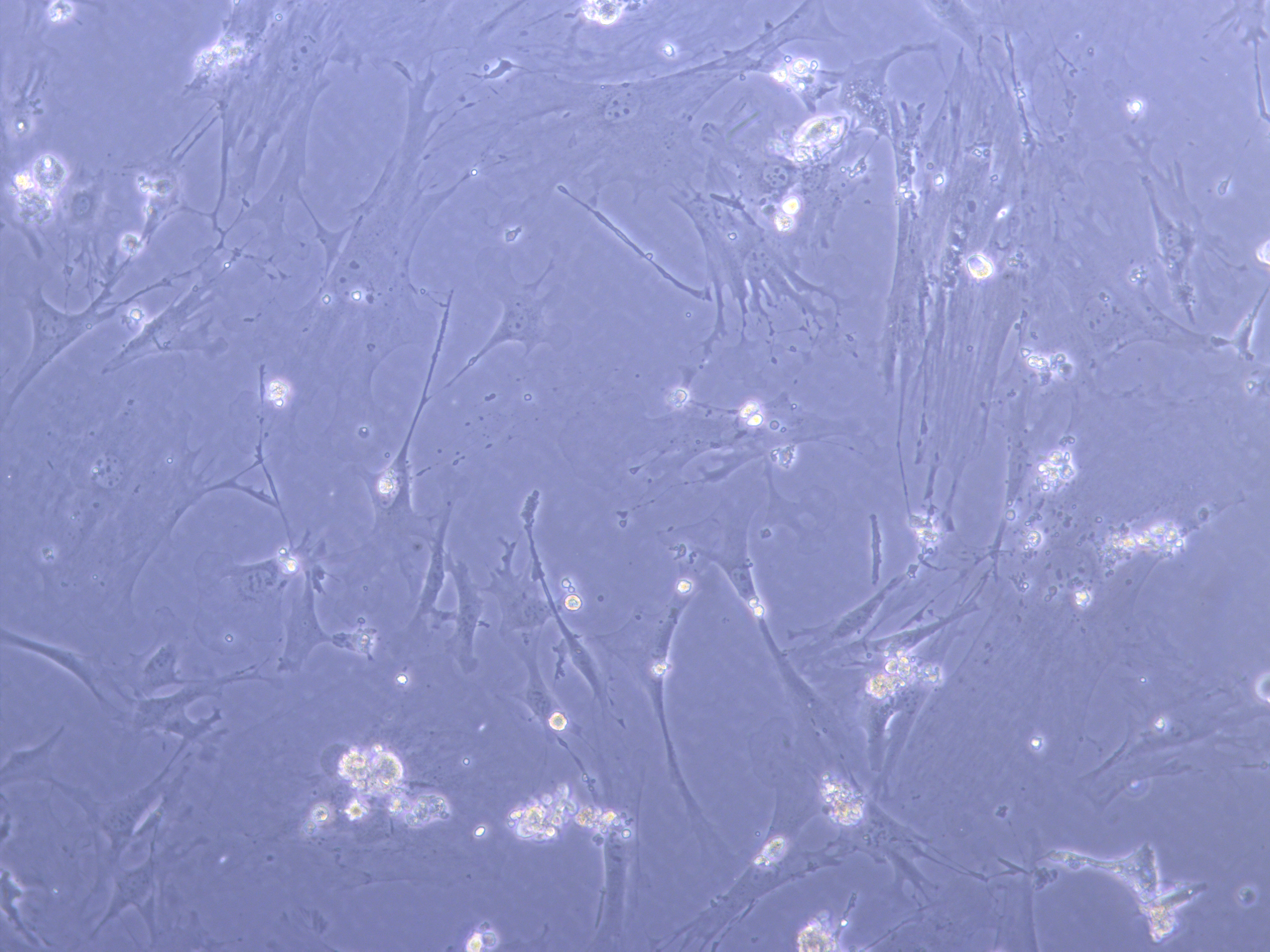

Supplement: Supplementary file 3 — Source data Fig. 1 [file 44318_2024_163_MOESM3_ESM.zip › Figure 1/1E/Light microscopy pic MEF ABT 7d.tif]

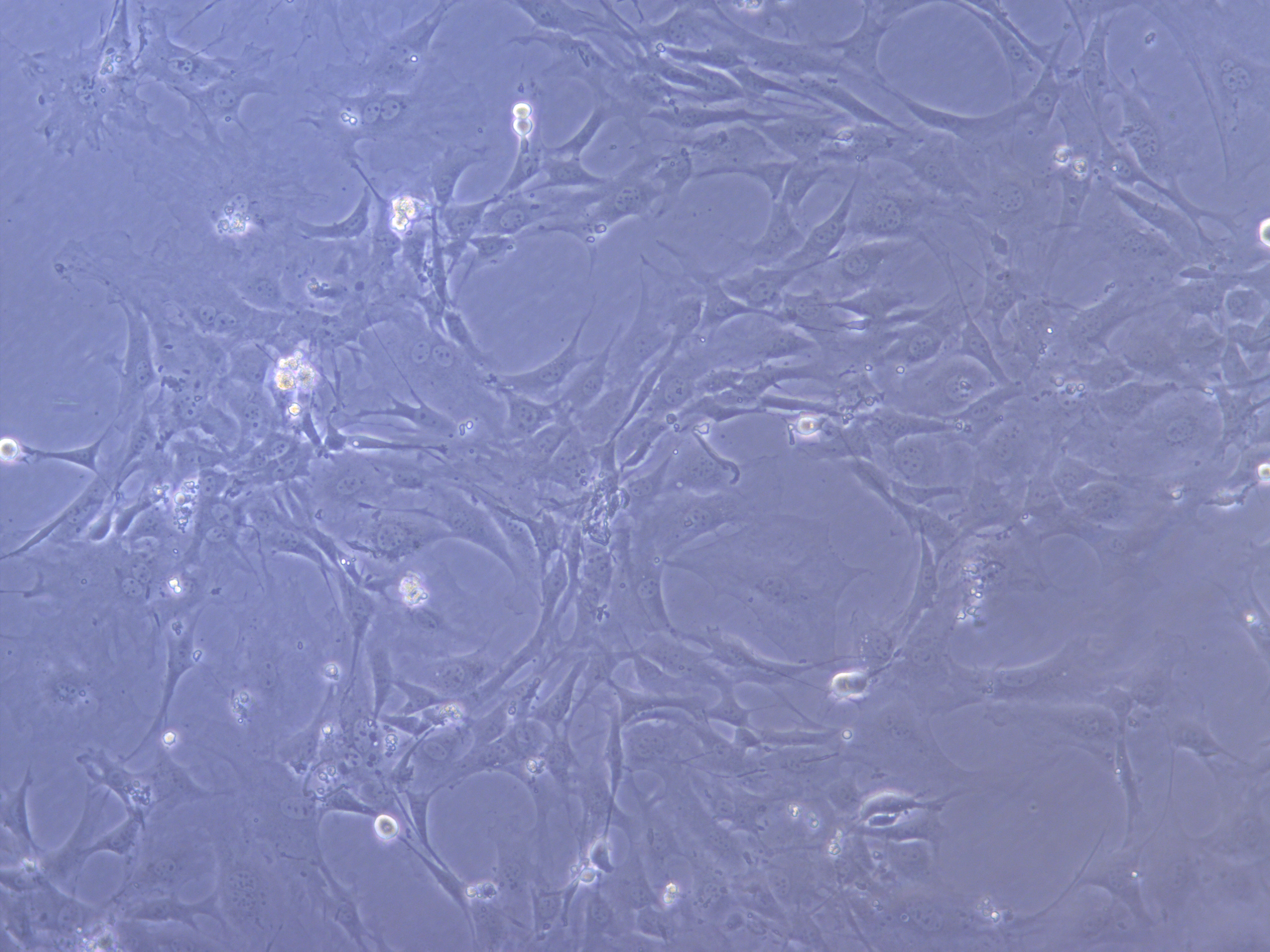

Supplement: Supplementary file 3 — Source data Fig. 1 [file 44318_2024_163_MOESM3_ESM.zip › Figure 1/1E/Light microscopy pic MEF DMSO 7d.tif]

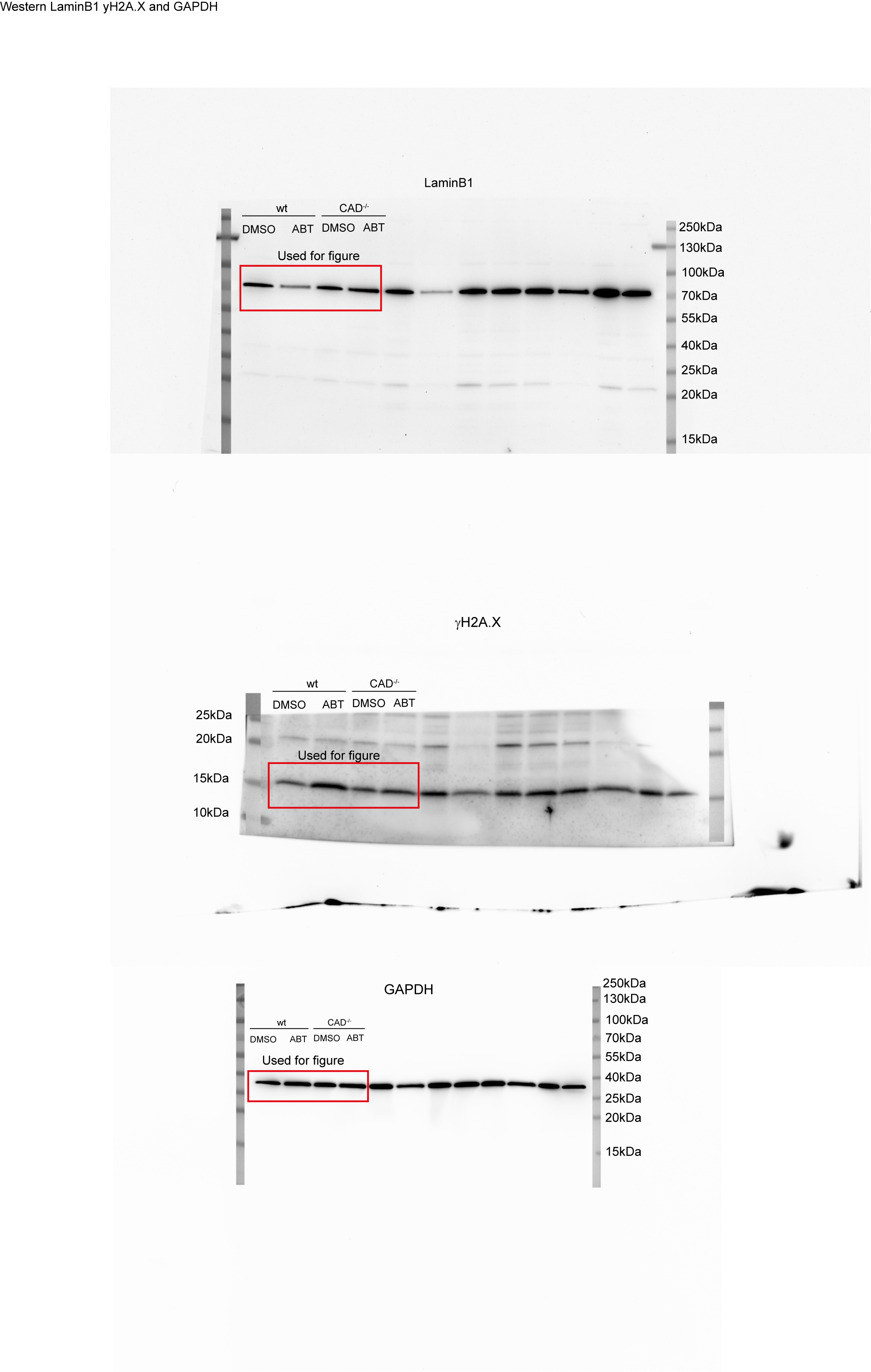

Supplement: Supplementary file 3 — Source data Fig. 1 [file 44318_2024_163_MOESM3_ESM.zip › Figure 1/1F/Western yH2AX LaminB1 GAPDH MEF ABT.tif]

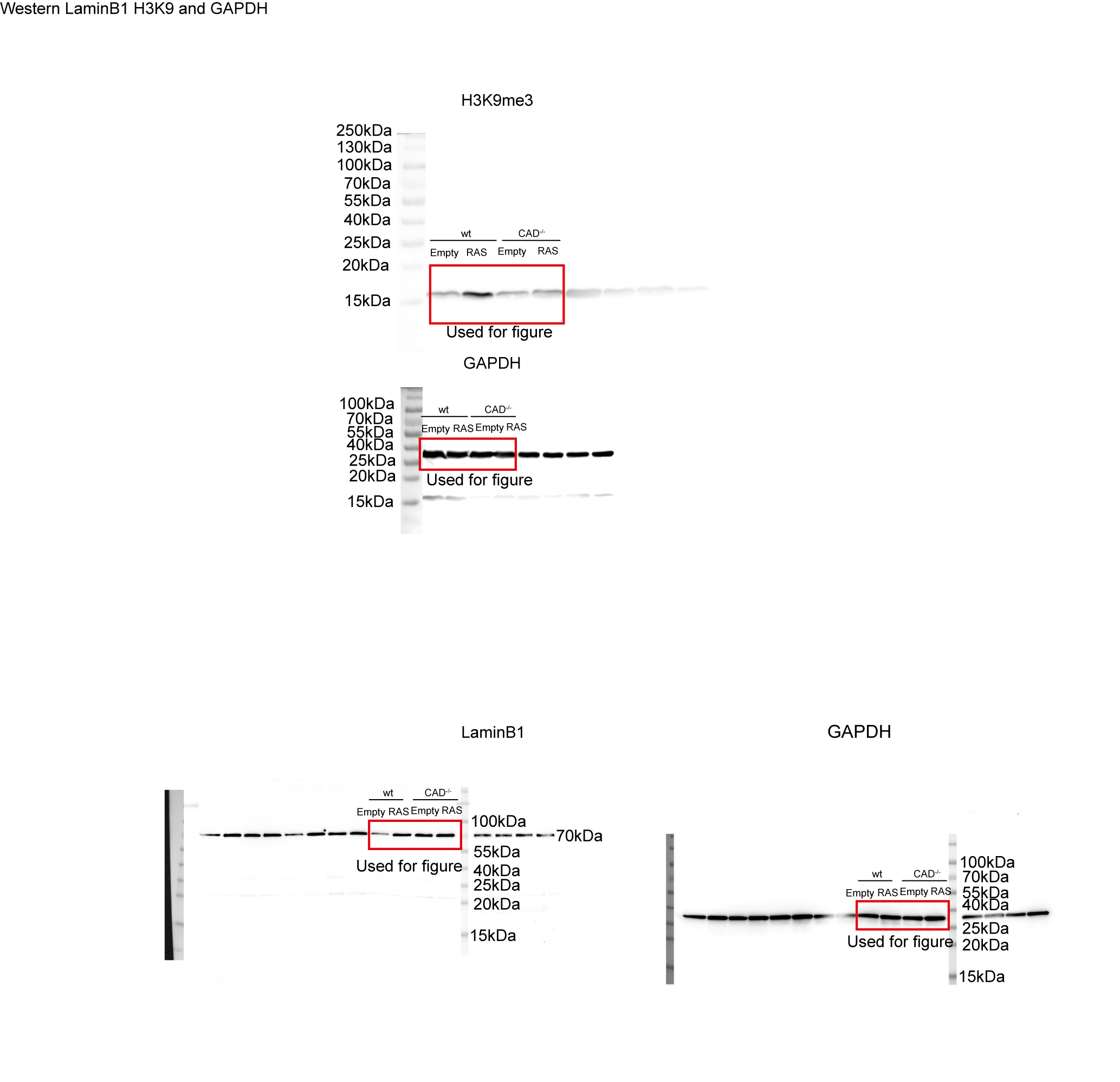

Supplement: Supplementary file 4 — Source data Fig. 2 [file 44318_2024_163_MOESM4_ESM.zip › Figure 2/2C/Western LaminB1 H3K9 GAPDH MEF RAS.tif]

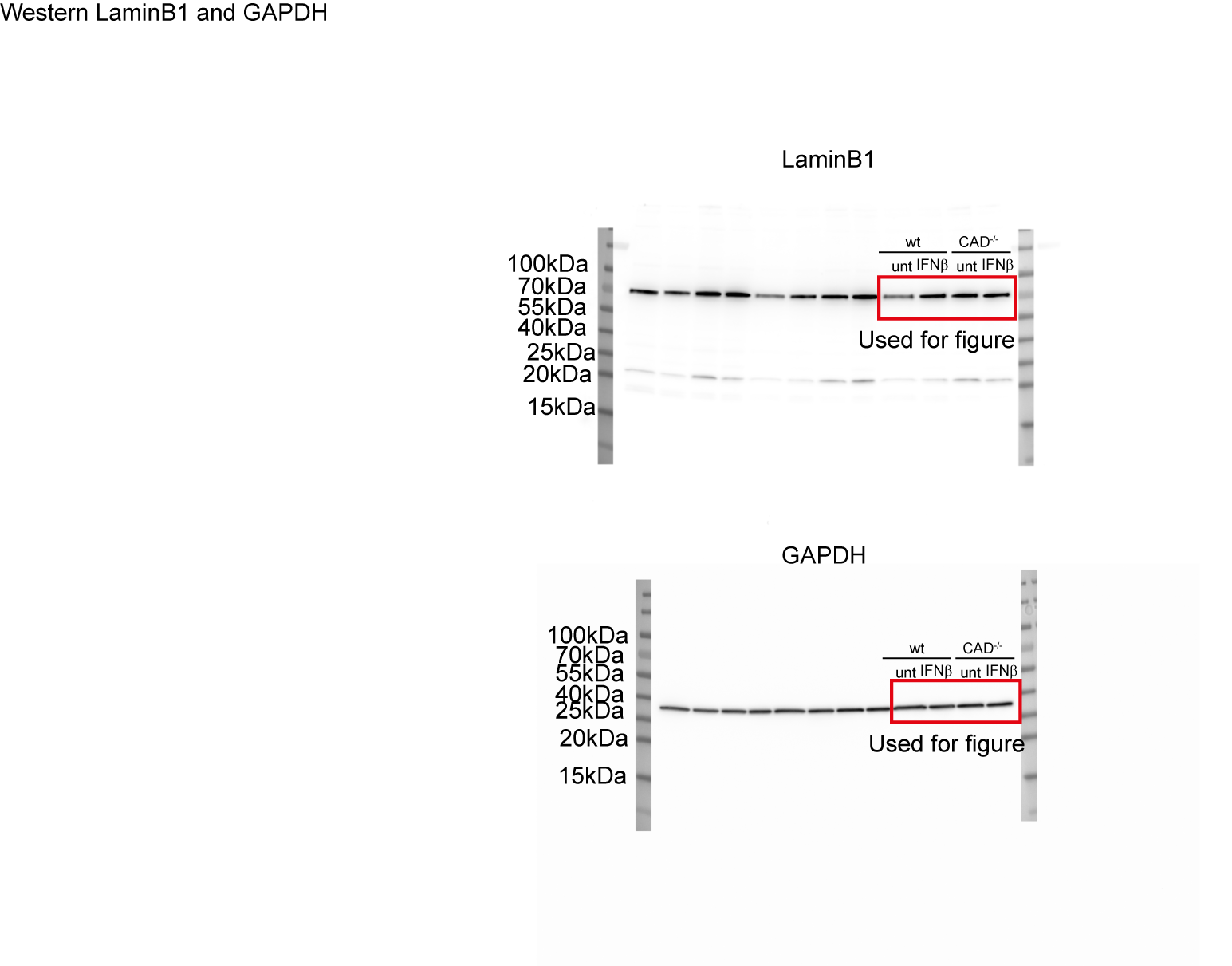

Supplement: Supplementary file 4 — Source data Fig. 2 [file 44318_2024_163_MOESM4_ESM.zip › Figure 2/2G/Western LaminB1 GAPDH MEF IFNb.tif]

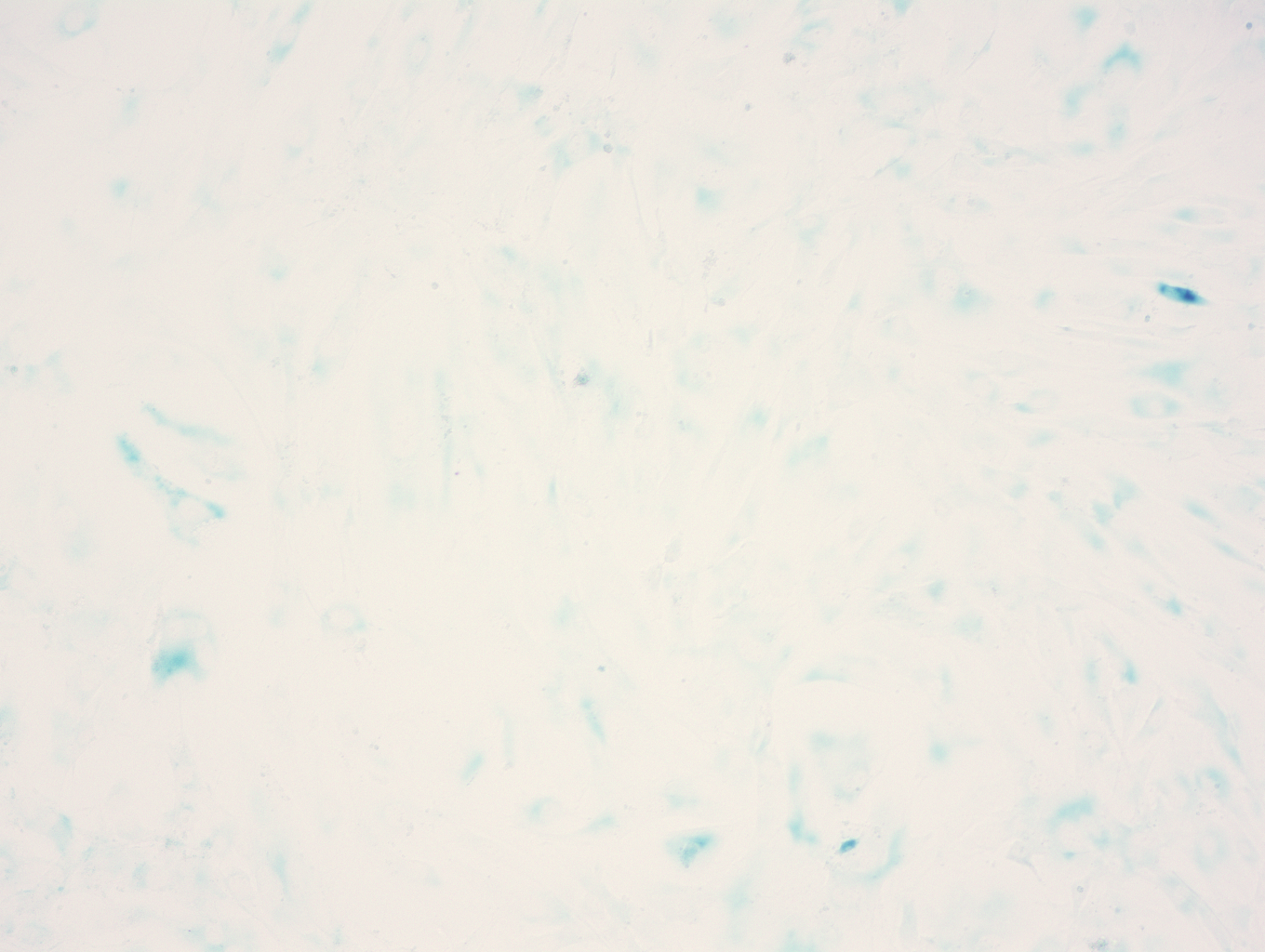

Supplement: Supplementary file 5 — Source data Fig. 3 [file 44318_2024_163_MOESM5_ESM.zip › Figure 3/3A/betaGal CAD KO d33.tif]

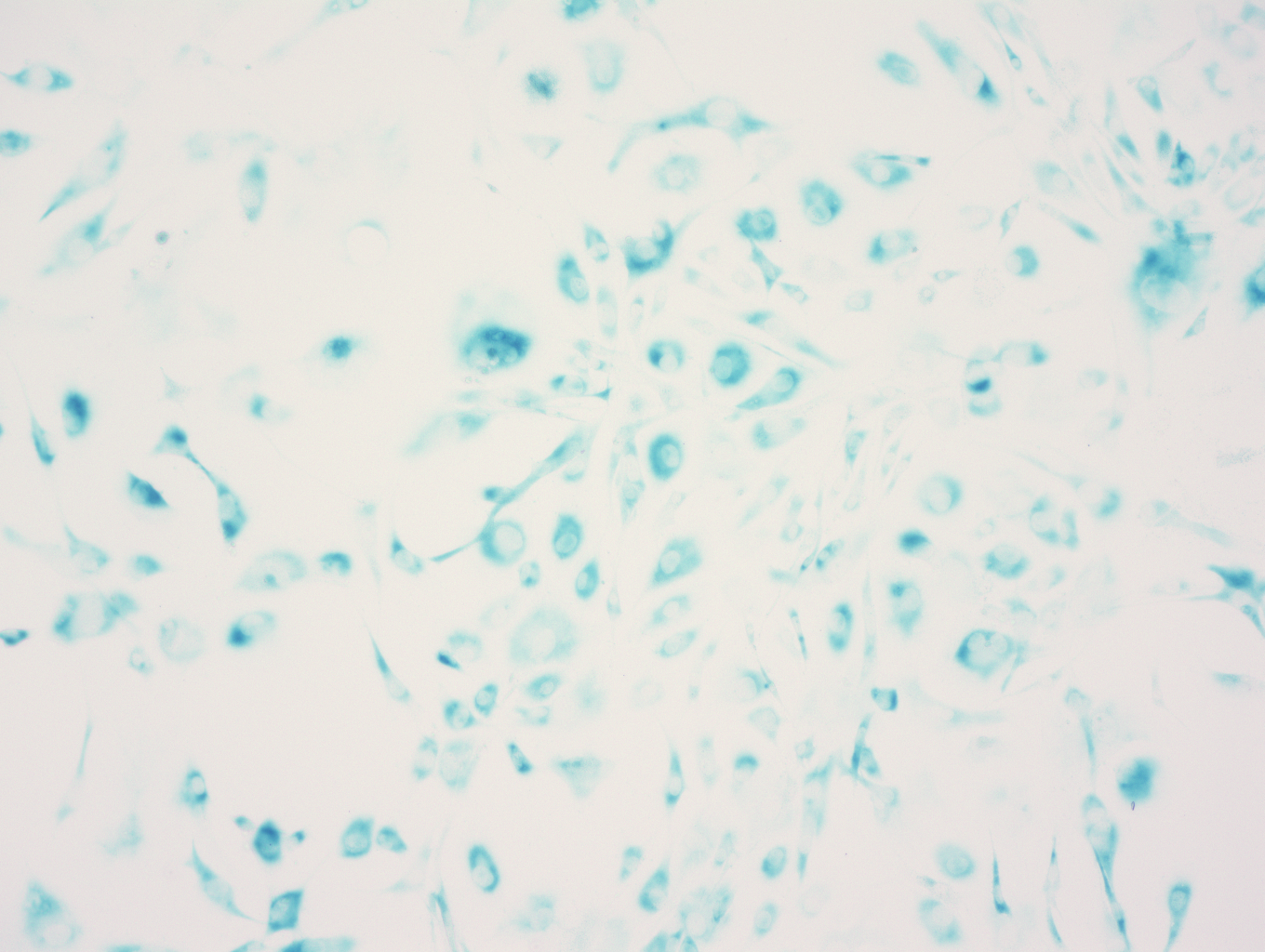

Supplement: Supplementary file 5 — Source data Fig. 3 [file 44318_2024_163_MOESM5_ESM.zip › Figure 3/3A/betaGal wt d33.tif]

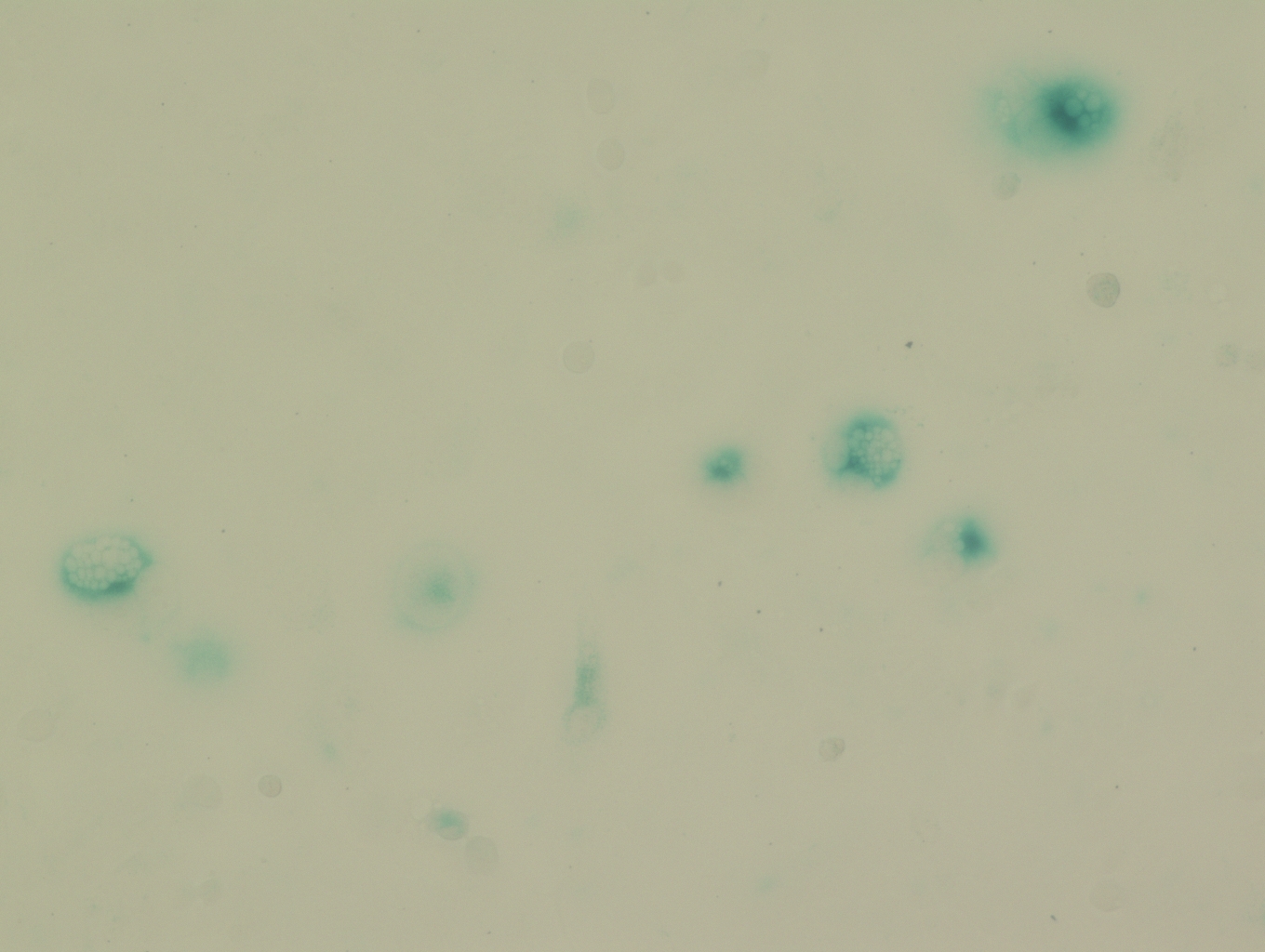

Supplement: Supplementary file 6 — Source data Fig. 4 [file 44318_2024_163_MOESM6_ESM.zip › Figure 4/4B/betaGal.jpg]

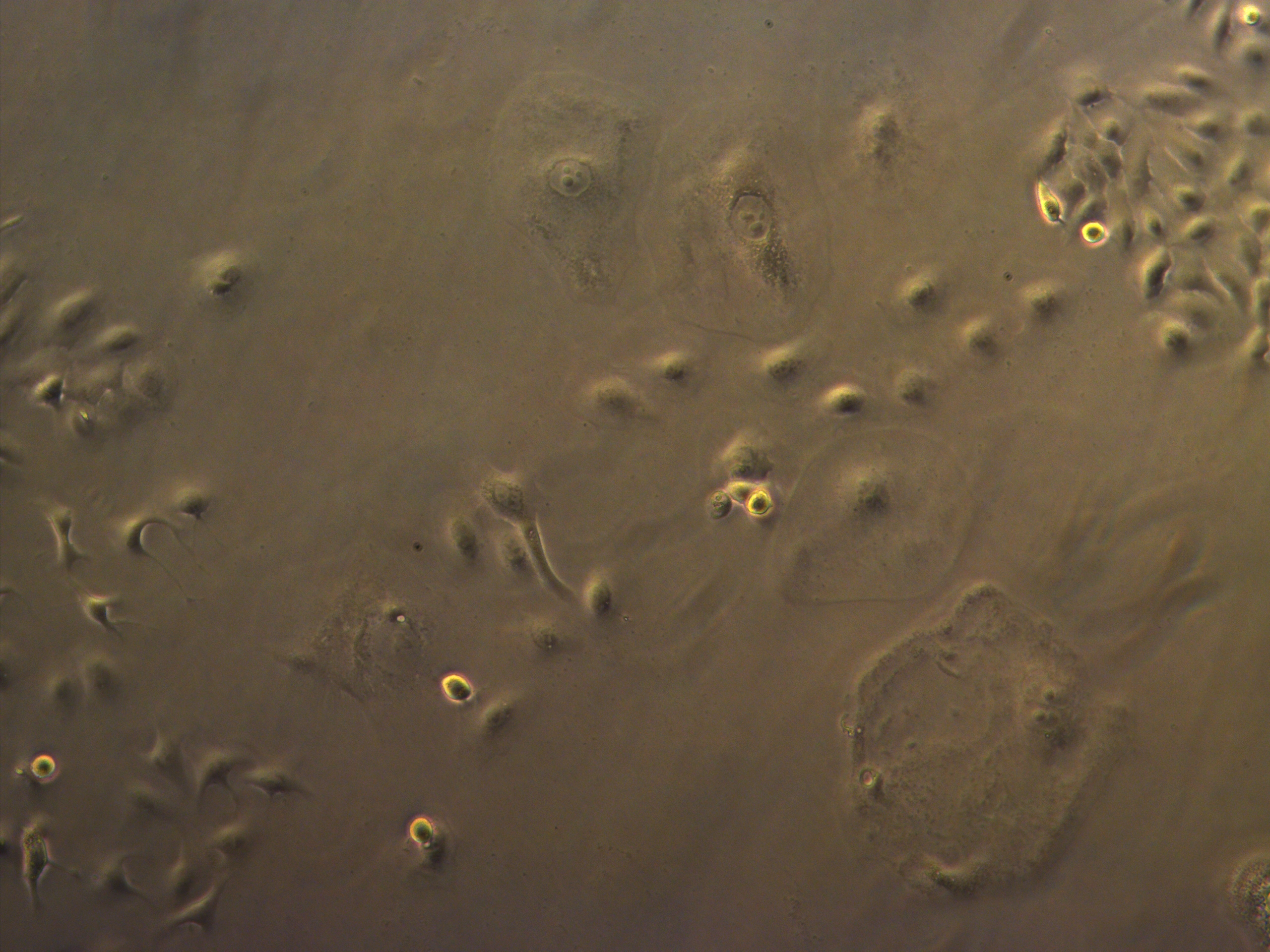

Supplement: Supplementary file 6 — Source data Fig. 4 [file 44318_2024_163_MOESM6_ESM.zip › Figure 4/4B/Light microscopy.jpg]

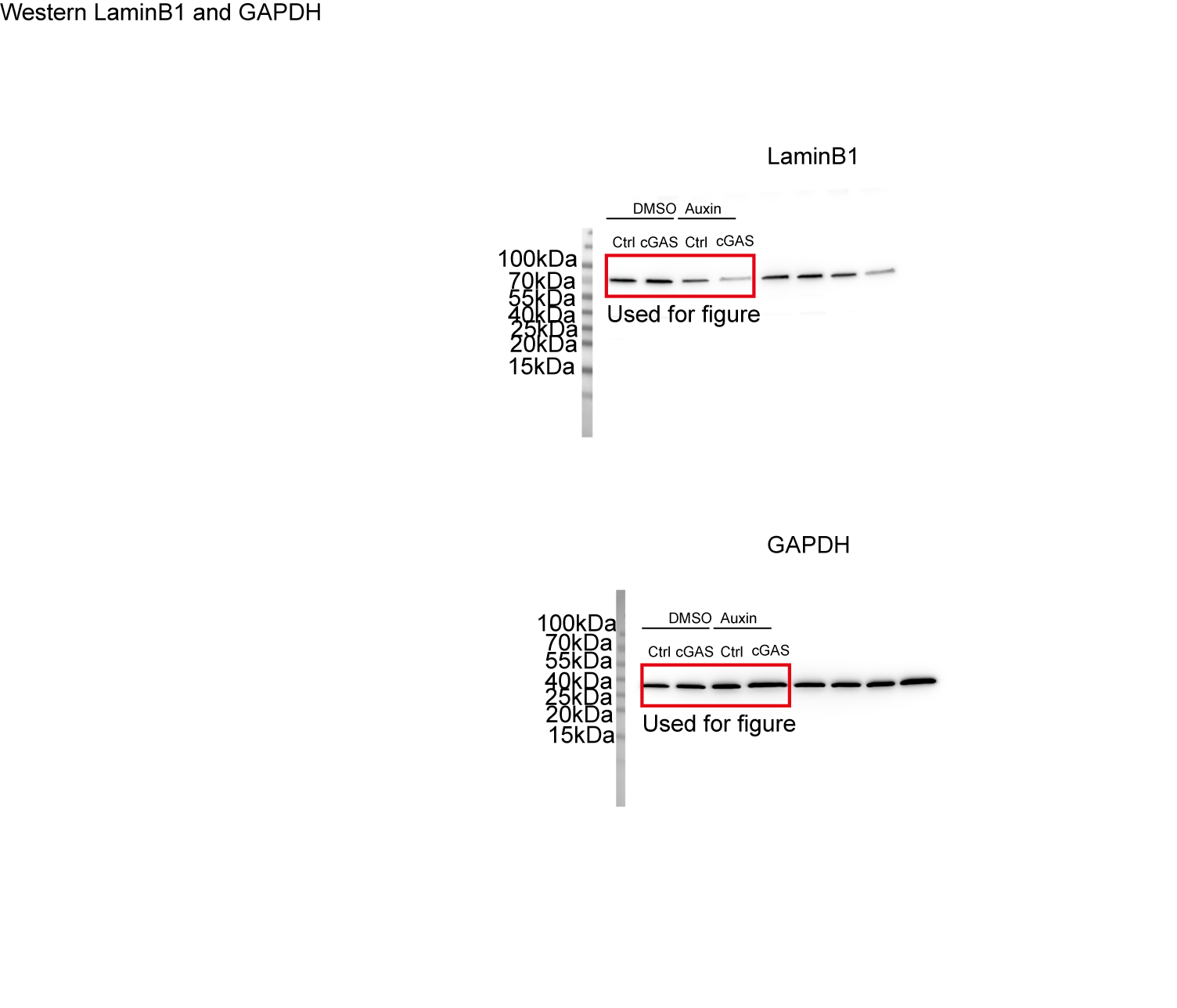

Supplement: Supplementary file 6 — Source data Fig. 4 [file 44318_2024_163_MOESM6_ESM.zip › Figure 4/4C/Western LaminB1 GAPDH HaCaT.tif]

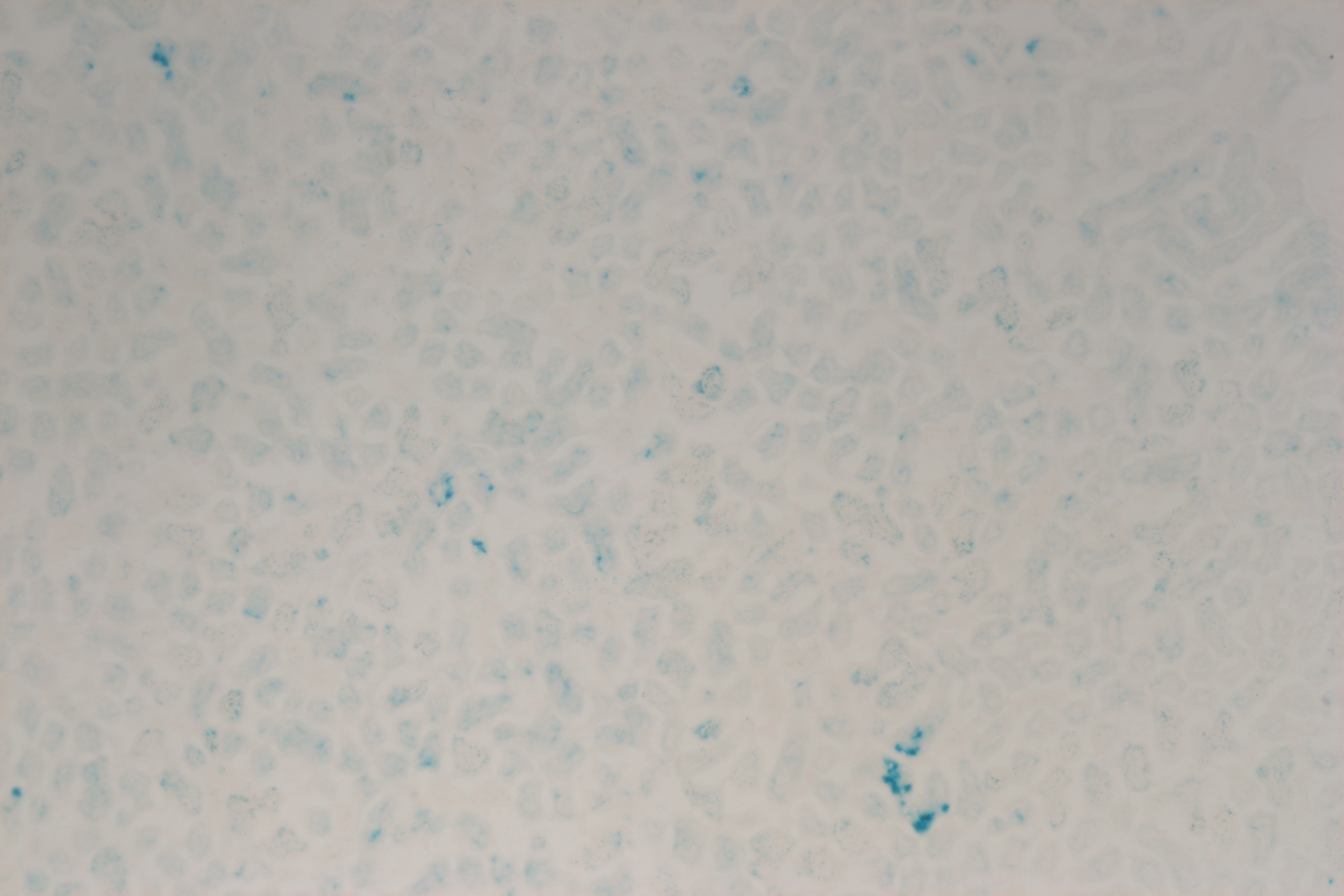

Supplement: Supplementary file 7 — Source data Fig. 5 [file 44318_2024_163_MOESM7_ESM.zip › Figure 5/5A/betaGal CAD KO.jpg]

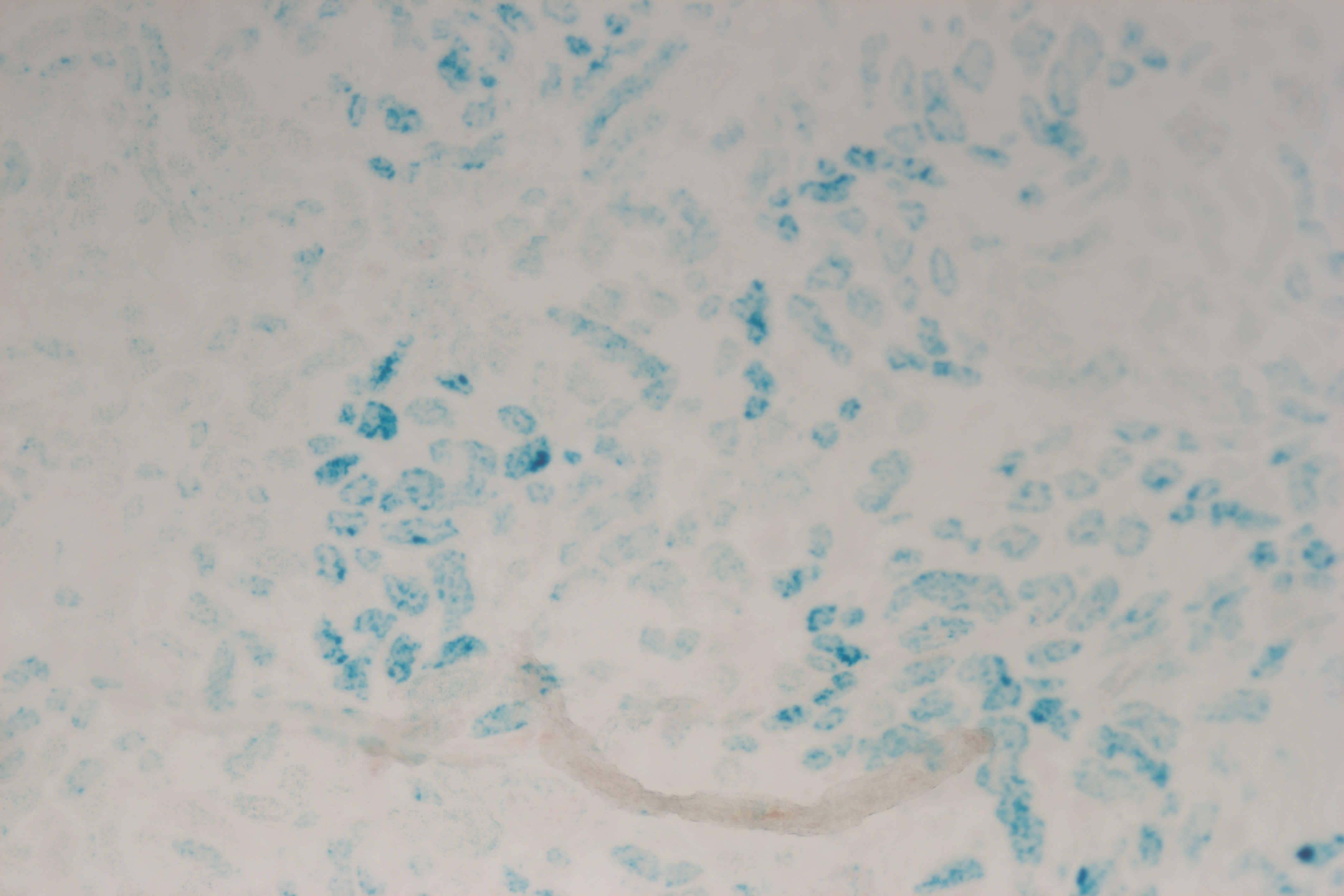

Supplement: Supplementary file 7 — Source data Fig. 5 [file 44318_2024_163_MOESM7_ESM.zip › Figure 5/5A/betaGal wt.jpg]

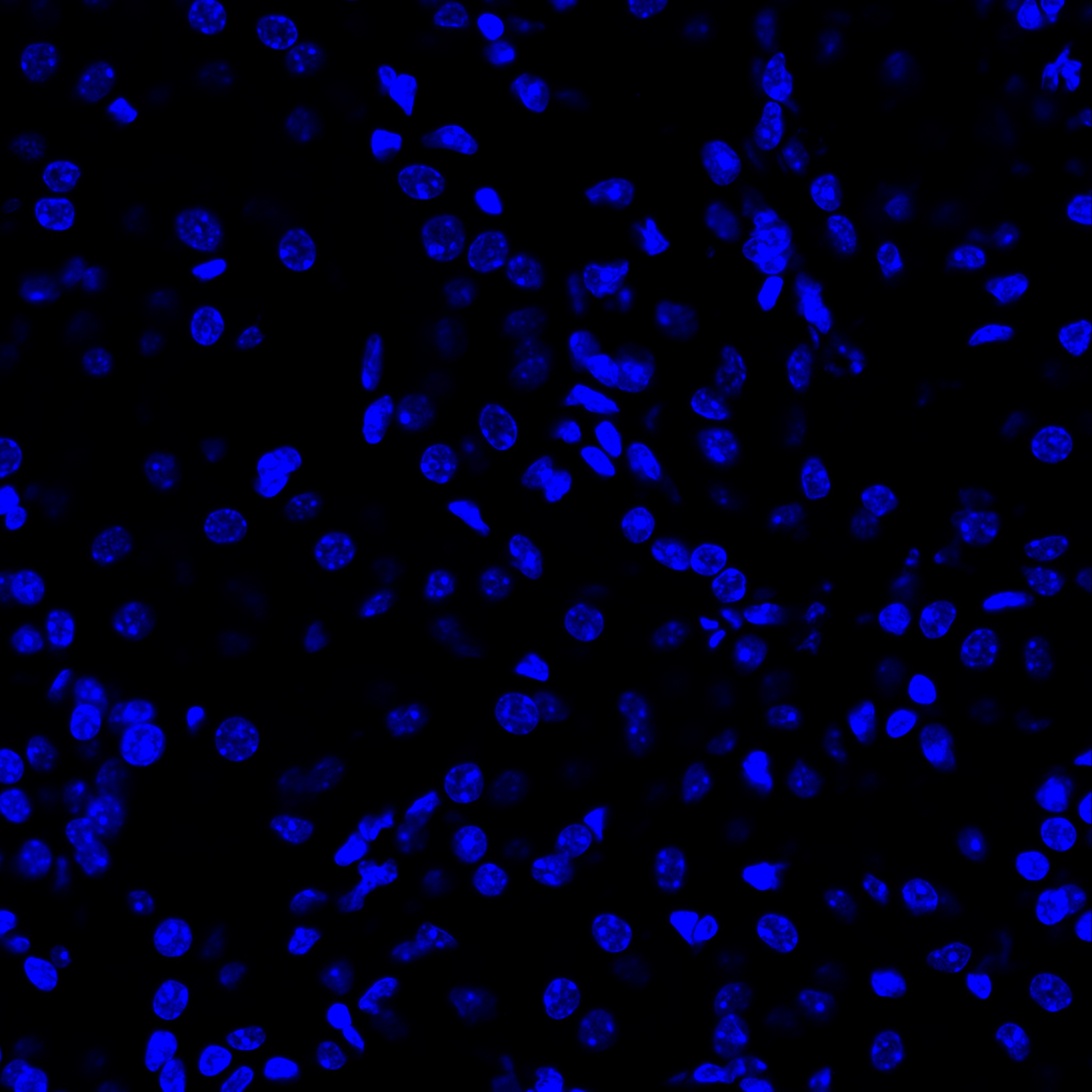

Supplement: Supplementary file 7 — Source data Fig. 5 [file 44318_2024_163_MOESM7_ESM.zip › Figure 5/5B/Microscopy CAD KO Kidney DAPI.jpg]

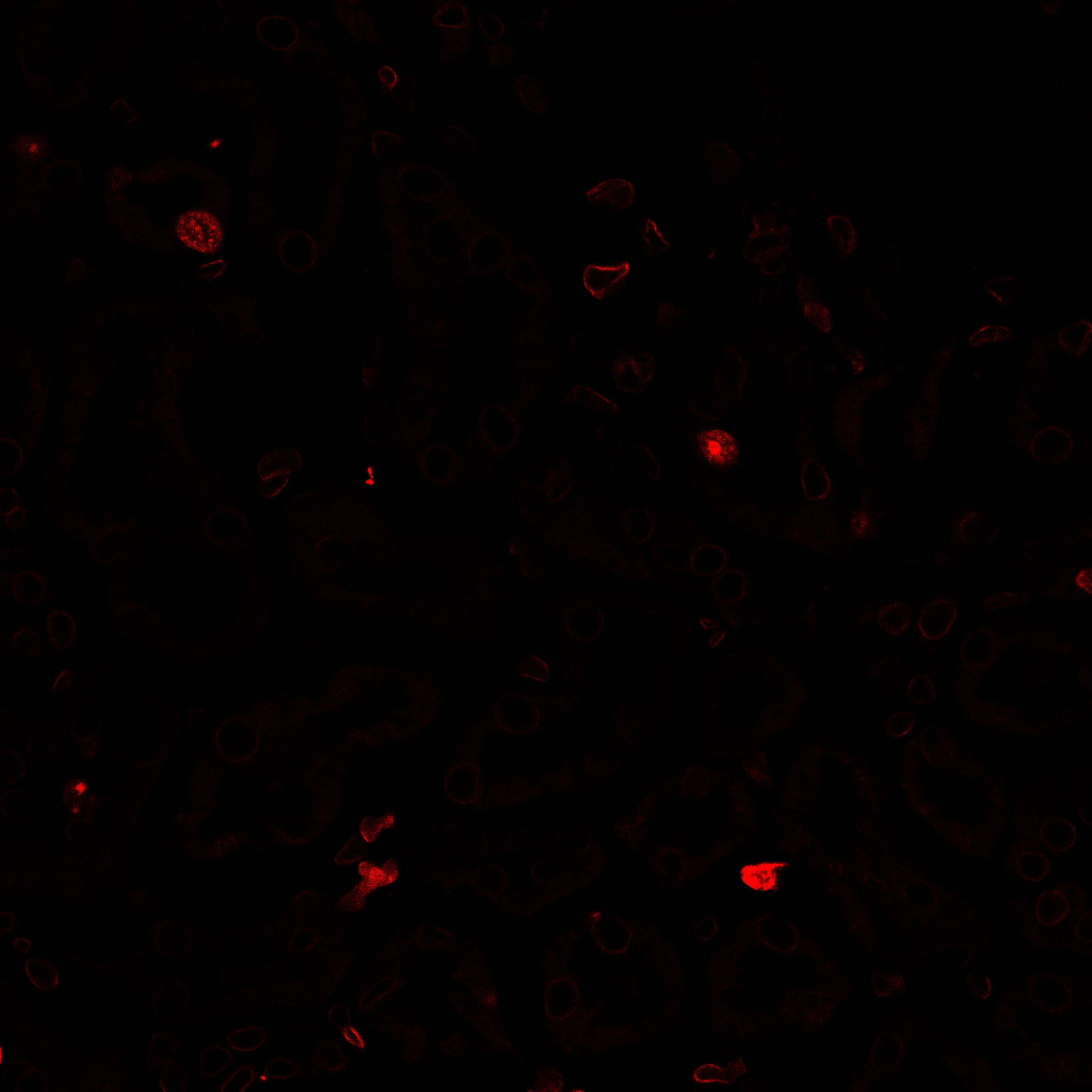

Supplement: Supplementary file 7 — Source data Fig. 5 [file 44318_2024_163_MOESM7_ESM.zip › Figure 5/5B/Microscopy CAD KO Kidney Ki67.jpg]

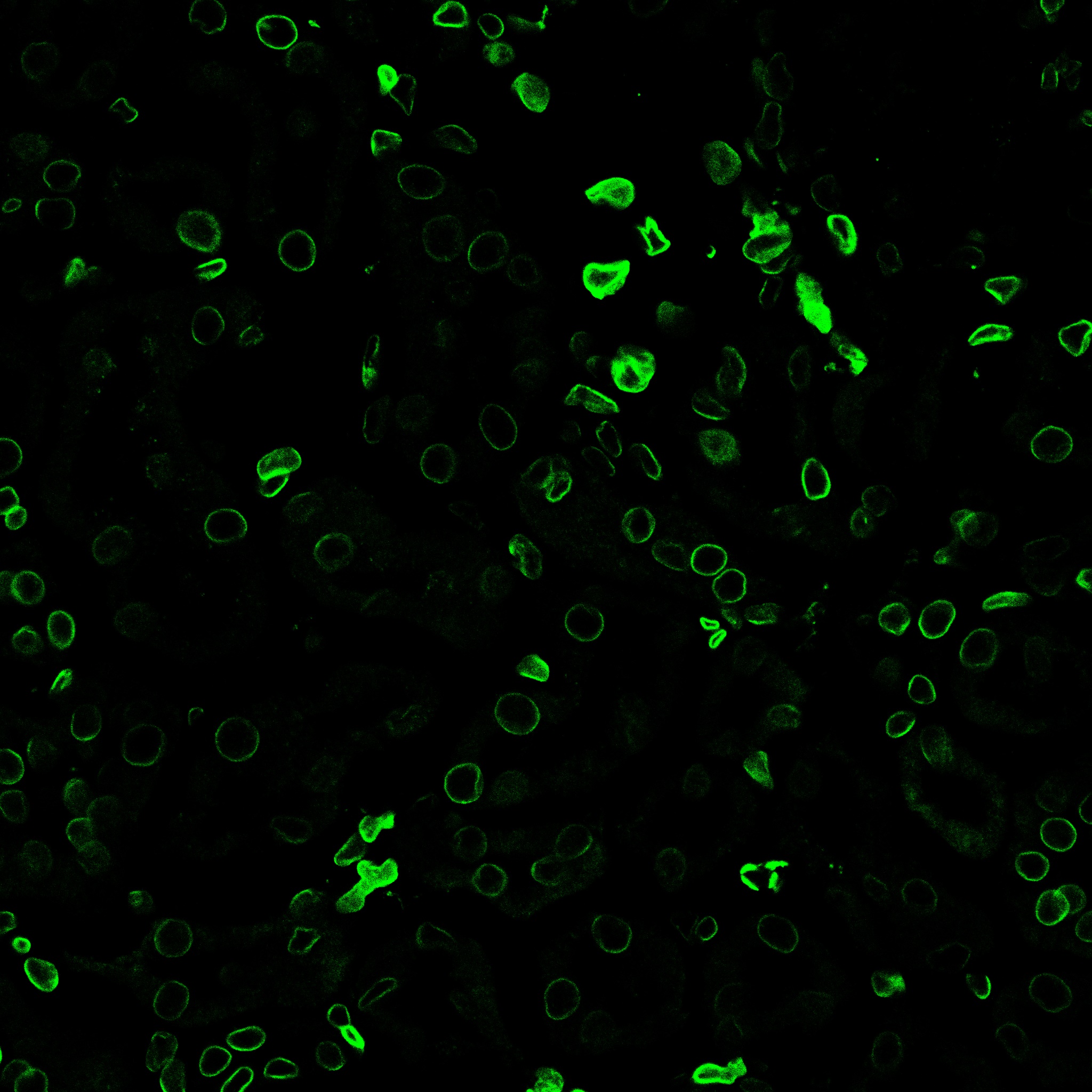

Supplement: Supplementary file 7 — Source data Fig. 5 [file 44318_2024_163_MOESM7_ESM.zip › Figure 5/5B/Microscopy CAD KO Kidney LaminB1.jpg]

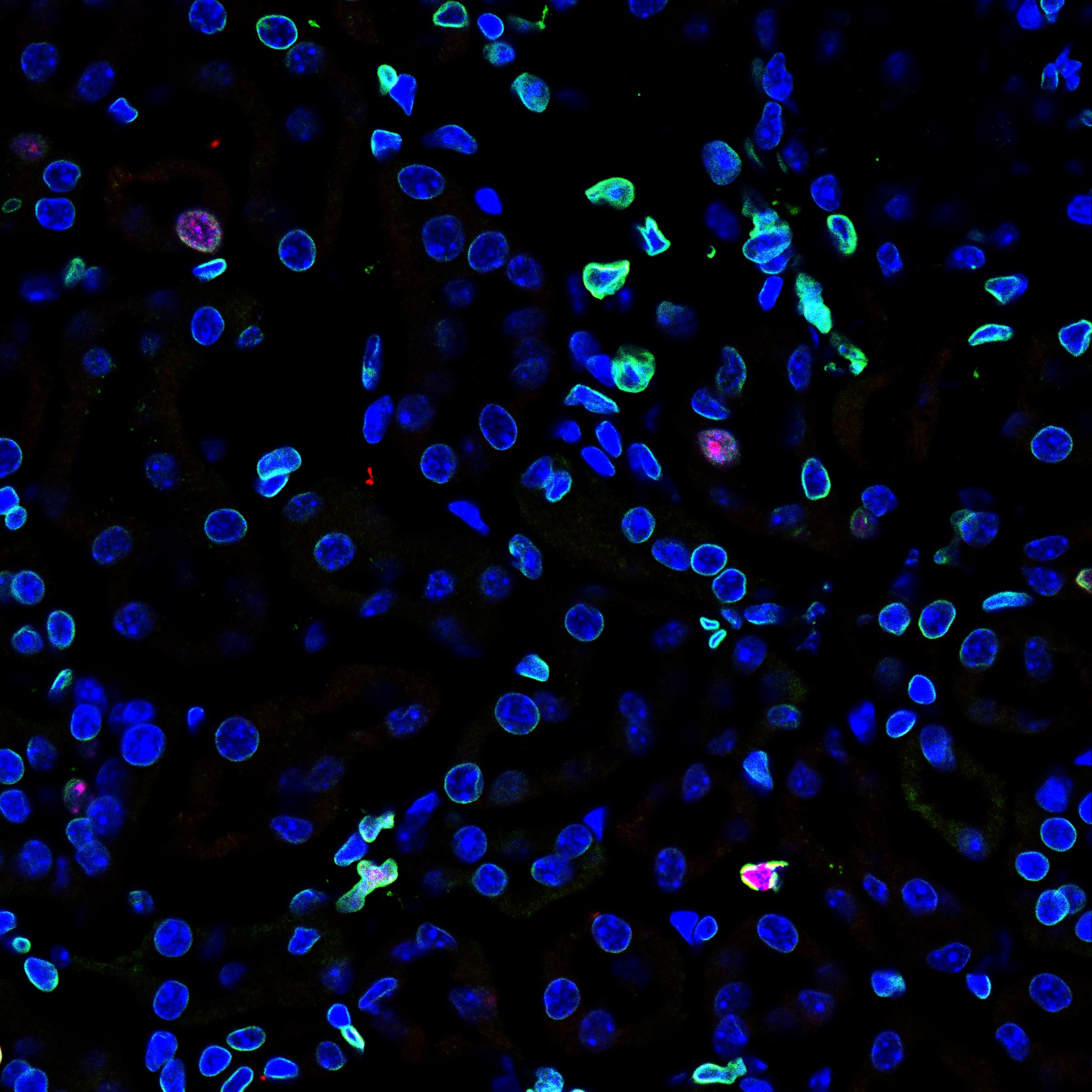

Supplement: Supplementary file 7 — Source data Fig. 5 [file 44318_2024_163_MOESM7_ESM.zip › Figure 5/5B/Microscopy CAD KO Kidney merged.jpg]

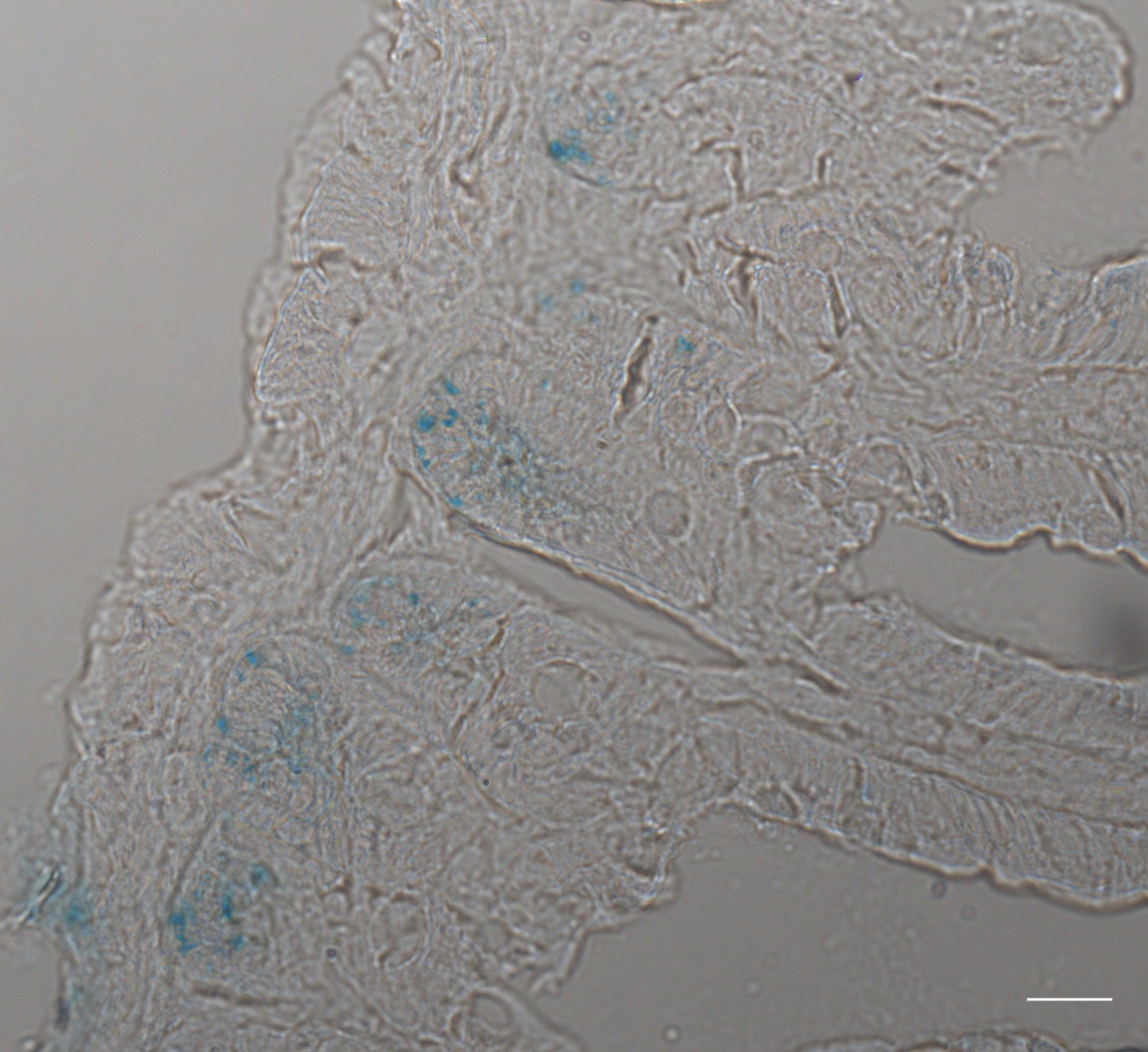

Supplement: Supplementary file 7 — Source data Fig. 5 [file 44318_2024_163_MOESM7_ESM.zip › Figure 5/5D/betaGal CAD KO.jpg]

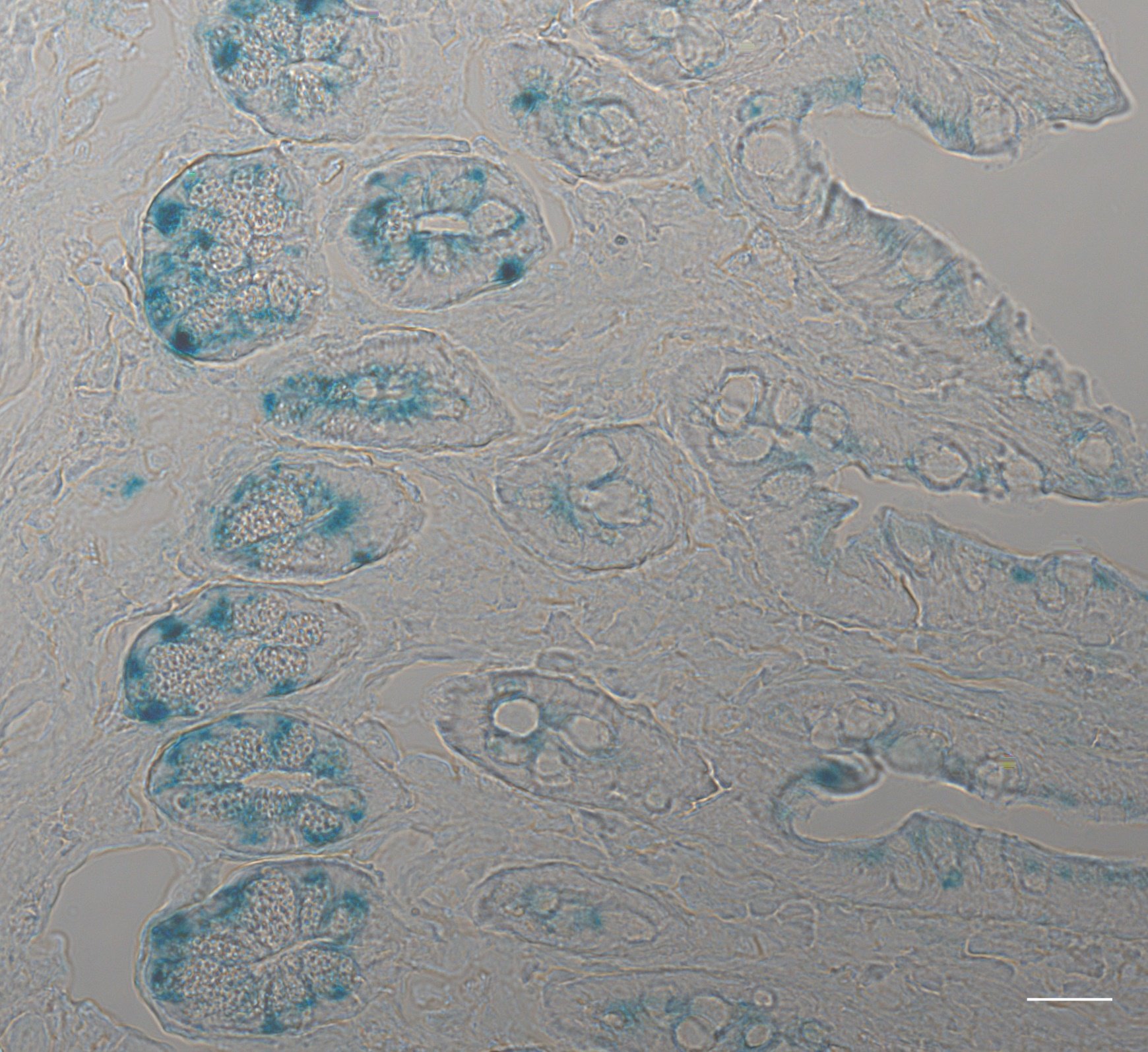

Supplement: Supplementary file 7 — Source data Fig. 5 [file 44318_2024_163_MOESM7_ESM.zip › Figure 5/5D/betaGal wt.jpg]

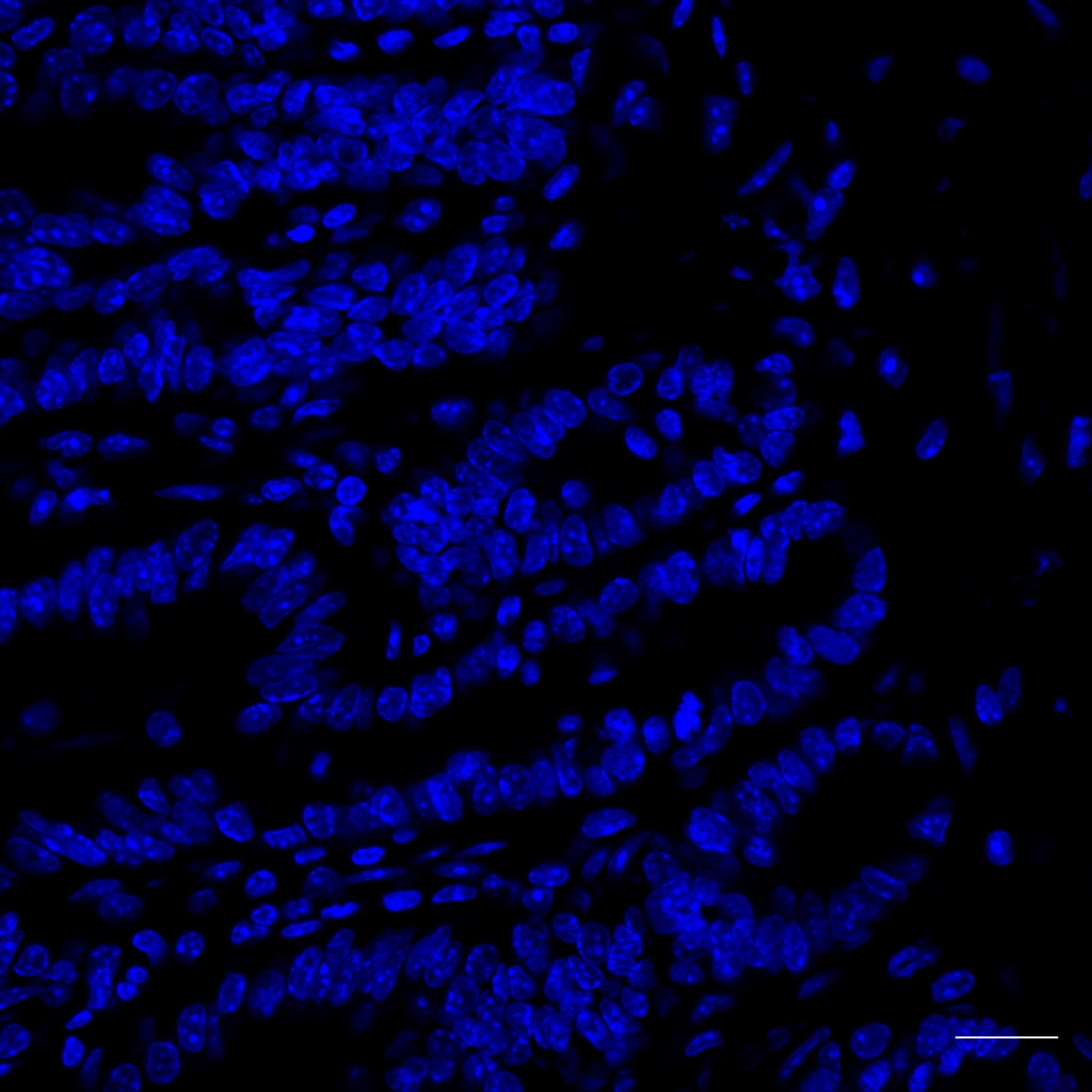

Supplement: Supplementary file 7 — Source data Fig. 5 [file 44318_2024_163_MOESM7_ESM.zip › Figure 5/5E/Microscopy small intestine CAD KO DAPI.jpg]

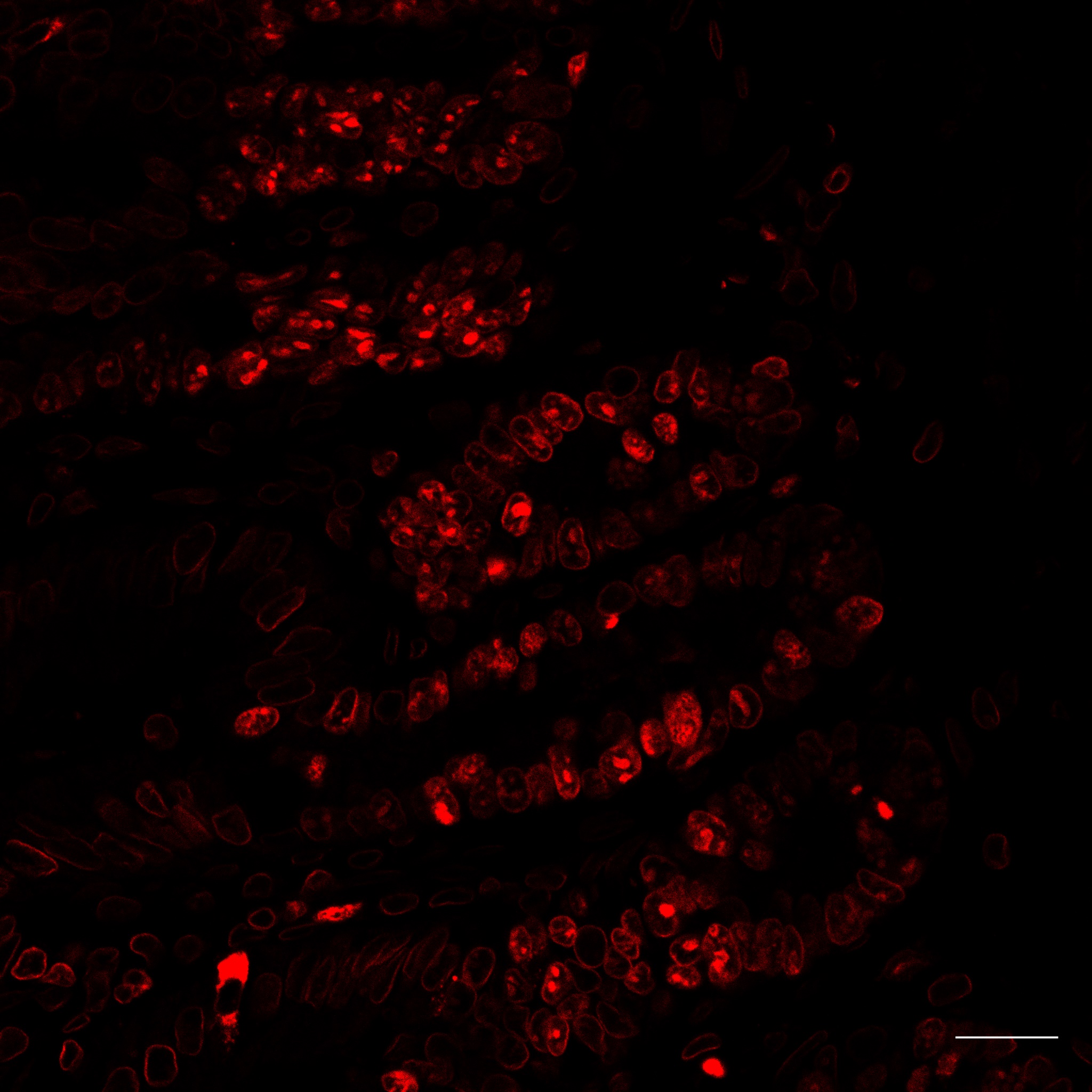

Supplement: Supplementary file 7 — Source data Fig. 5 [file 44318_2024_163_MOESM7_ESM.zip › Figure 5/5E/Microscopy small intestine CAD KO Ki67.jpg]

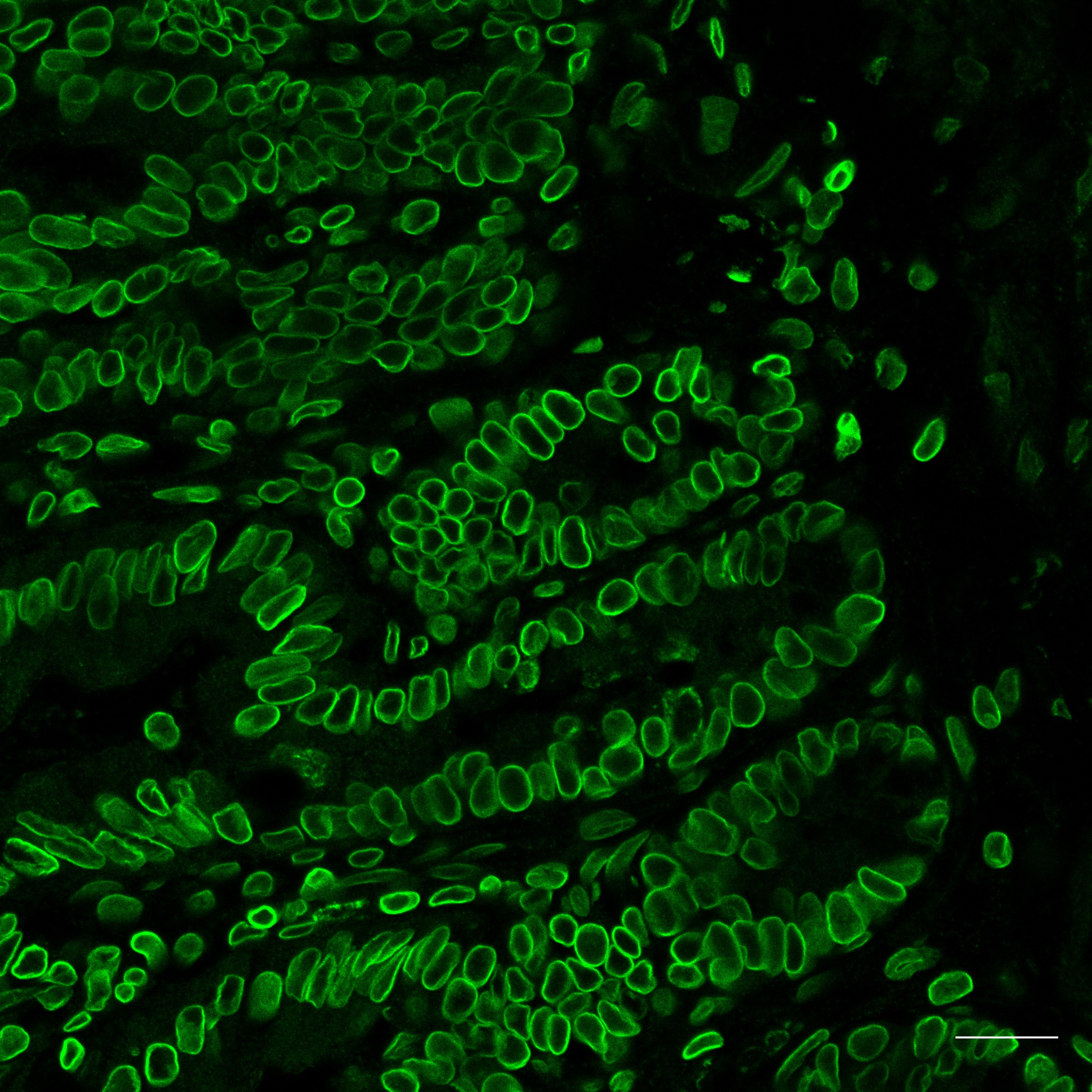

Supplement: Supplementary file 7 — Source data Fig. 5 [file 44318_2024_163_MOESM7_ESM.zip › Figure 5/5E/Microscopy small intestine CAD KO LaminB1.jpg]

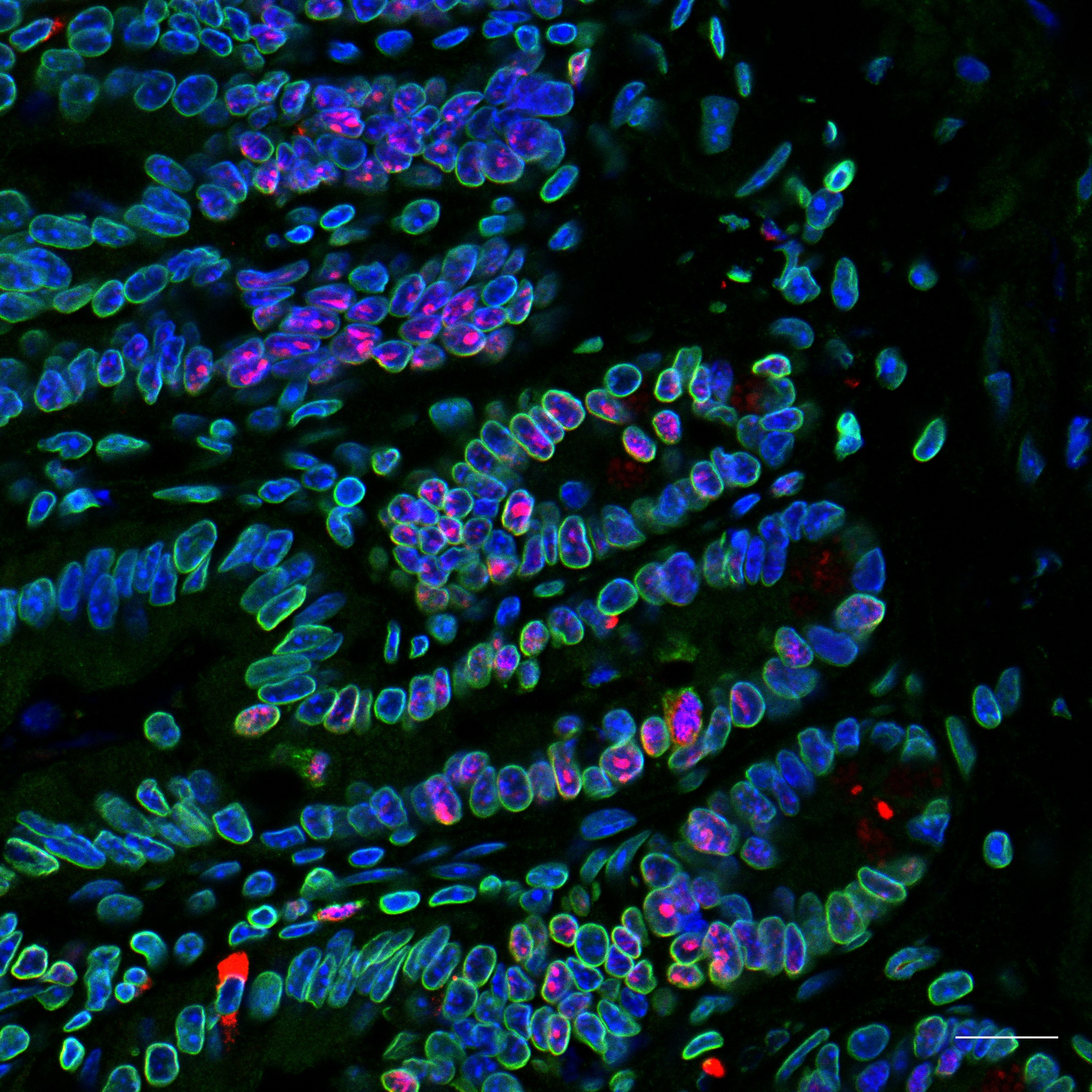

Supplement: Supplementary file 7 — Source data Fig. 5 [file 44318_2024_163_MOESM7_ESM.zip › Figure 5/5E/Microscopy small intestine CAD KO merged.jpg]

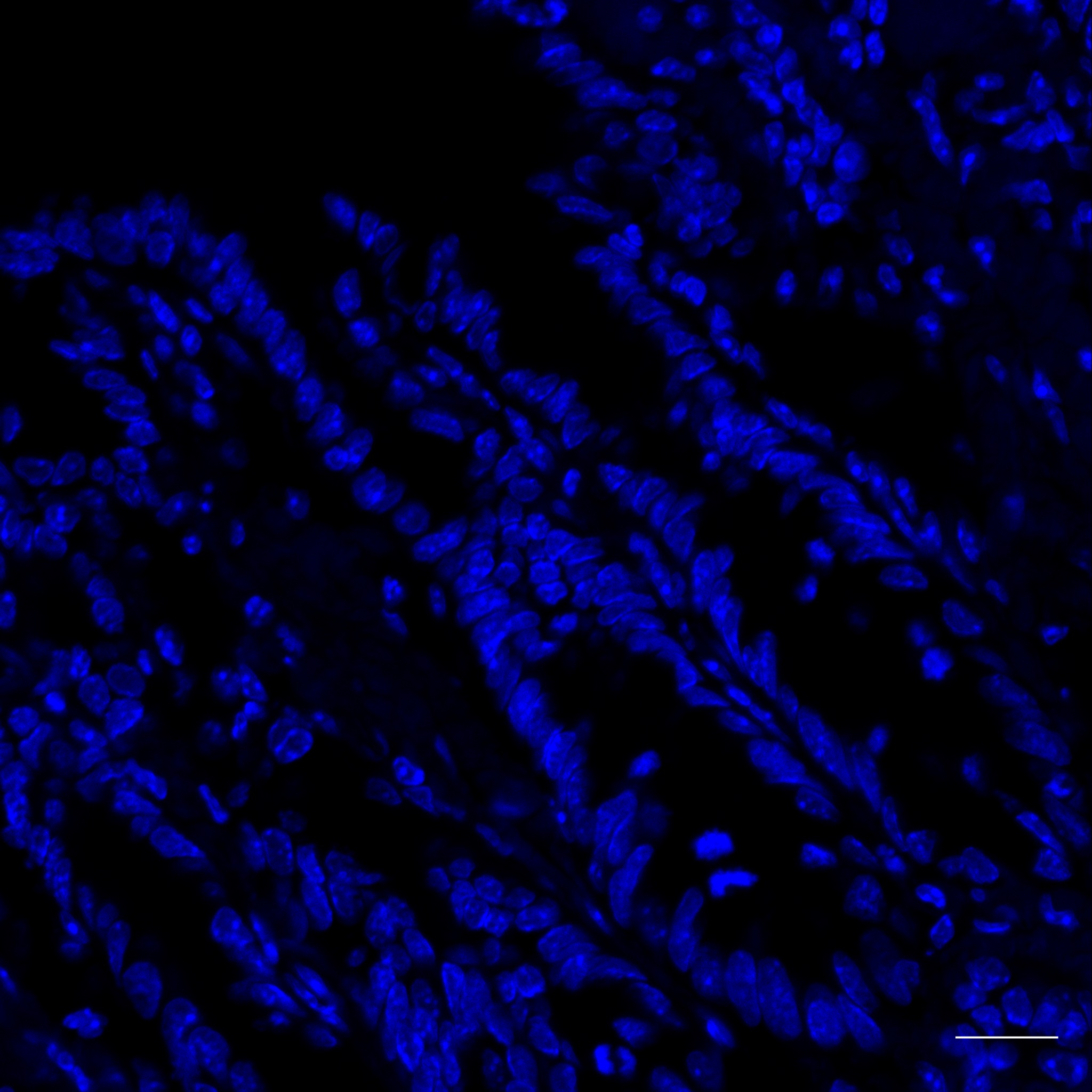

Supplement: Supplementary file 7 — Source data Fig. 5 [file 44318_2024_163_MOESM7_ESM.zip › Figure 5/5E/Microscopy small intestine wt DAPI.jpg]

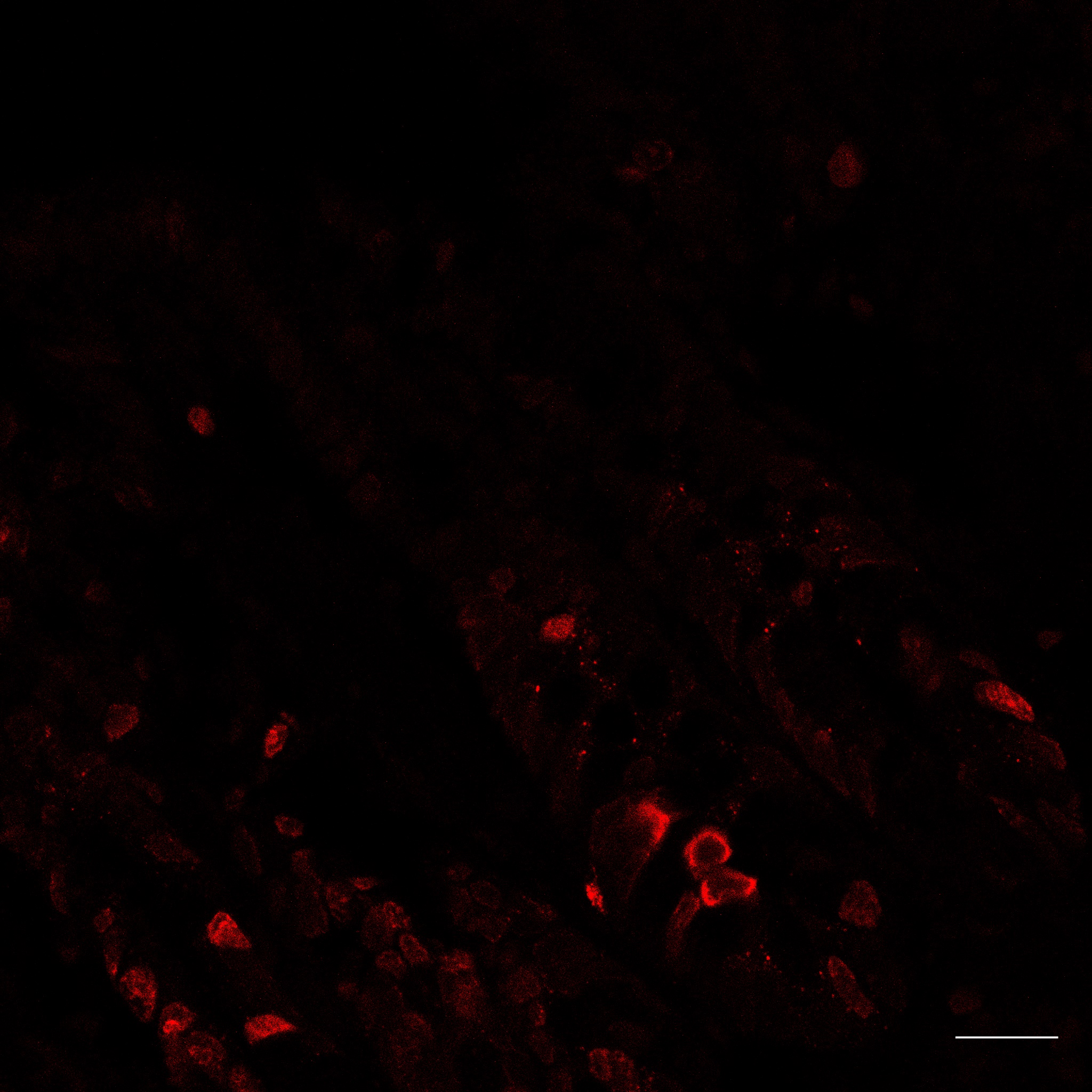

Supplement: Supplementary file 7 — Source data Fig. 5 [file 44318_2024_163_MOESM7_ESM.zip › Figure 5/5E/Microscopy small intestine wt Ki67.jpg]

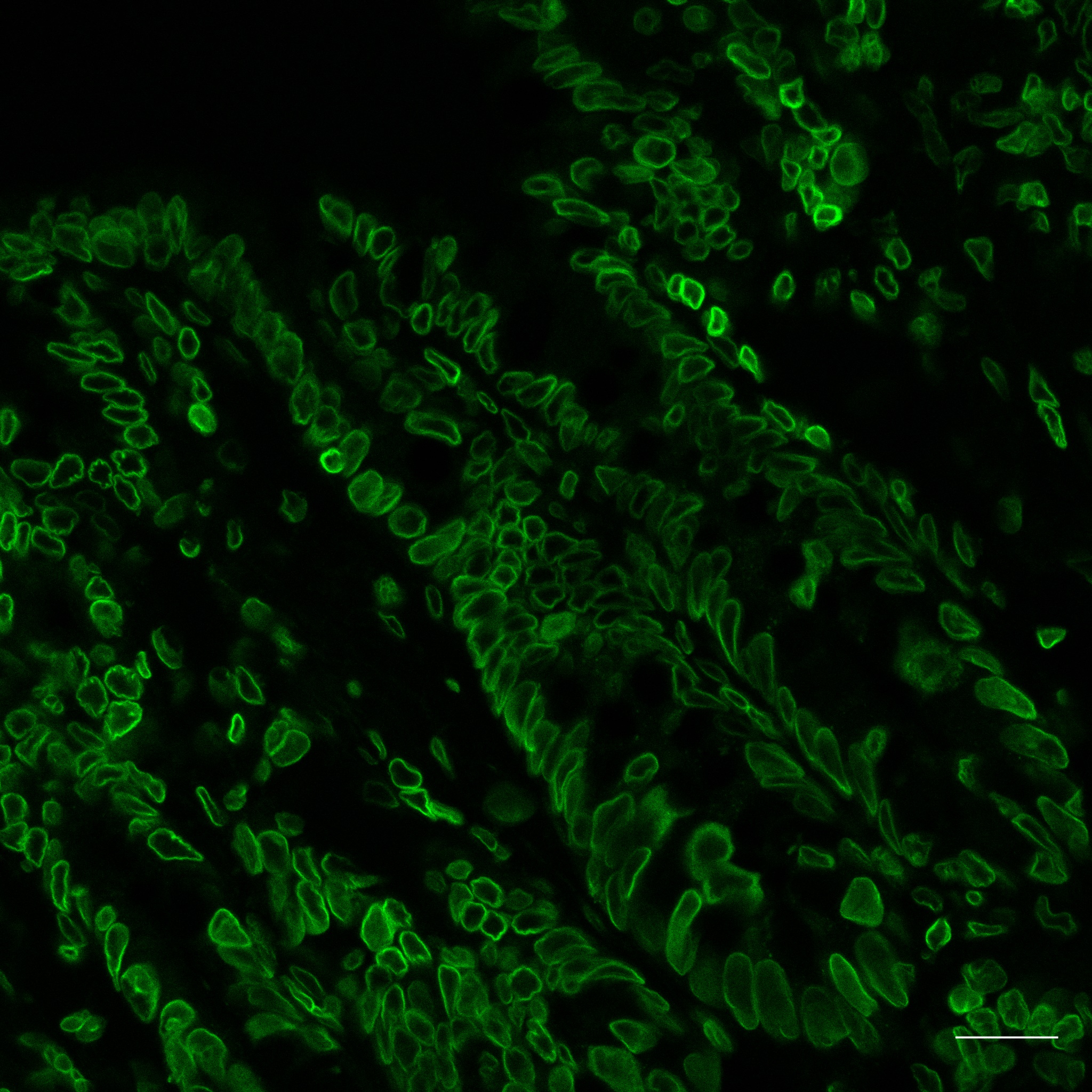

Supplement: Supplementary file 7 — Source data Fig. 5 [file 44318_2024_163_MOESM7_ESM.zip › Figure 5/5E/Microscopy small intestine wt LaminB1.jpg]

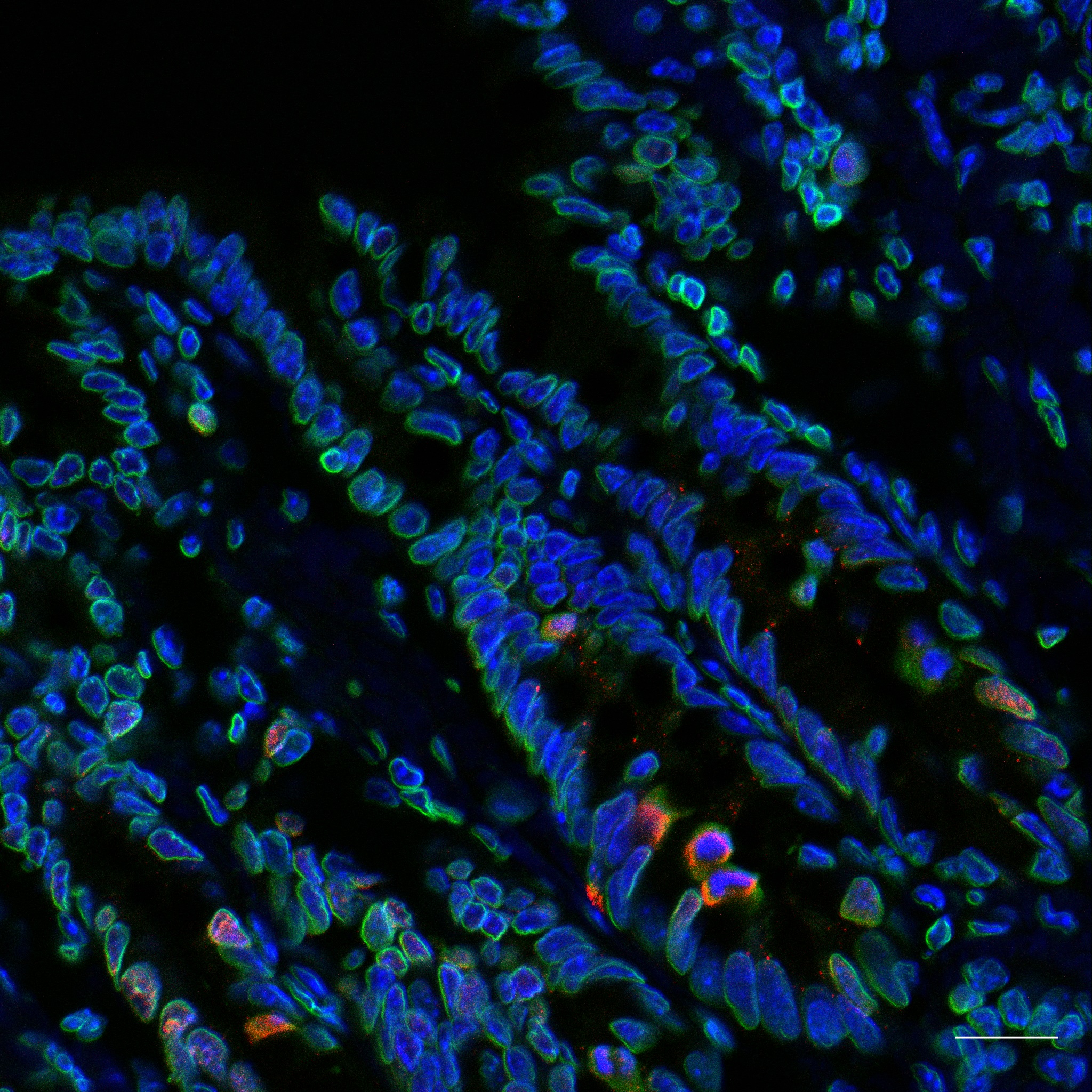

Supplement: Supplementary file 7 — Source data Fig. 5 [file 44318_2024_163_MOESM7_ESM.zip › Figure 5/5E/Microscopy small intestine wt merged.jpg]

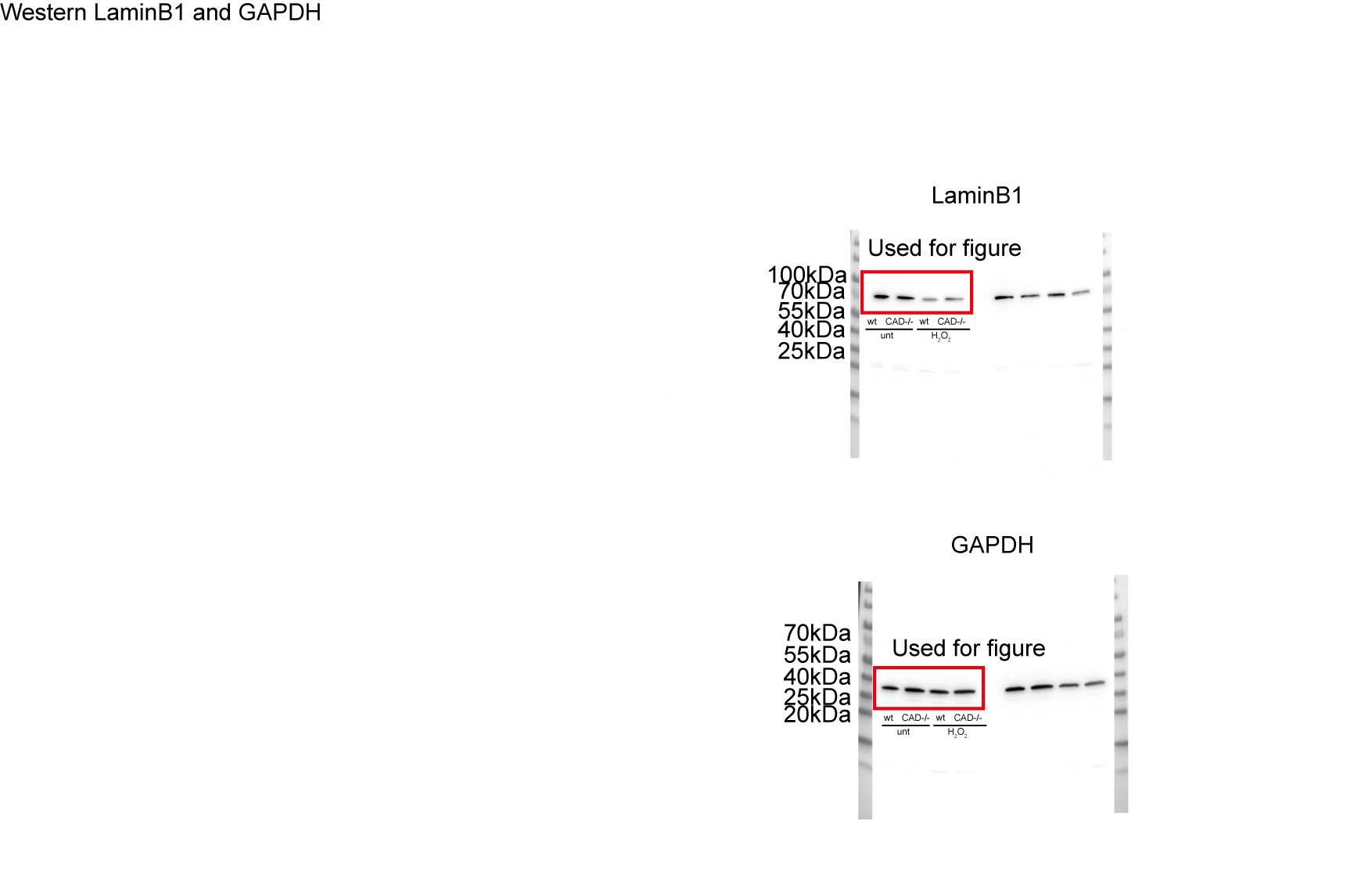

Supplement: Supplementary file 8 — EV and Appendix Figure Source Data [file 44318_2024_163_MOESM8_ESM.zip › Source Data for Expanded View and Appendix/Appendix Figure S2/S2/Western LaminB1 GAPDH MEF H2O2.tif]

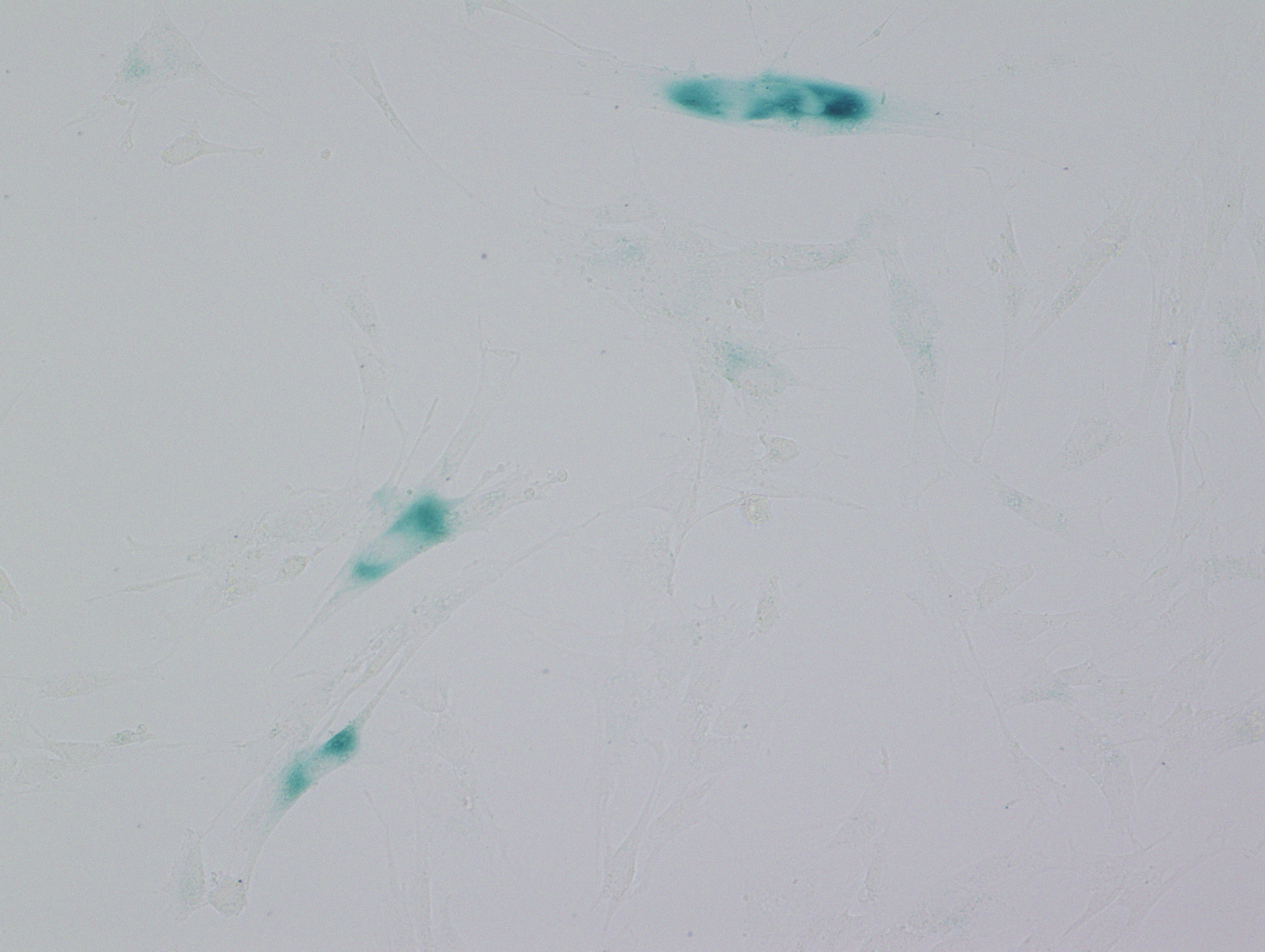

Supplement: Supplementary file 8 — EV and Appendix Figure Source Data [file 44318_2024_163_MOESM8_ESM.zip › Source Data for Expanded View and Appendix/Appendix Figure S3/S3A/betaGal WI38 Auxin.tif]

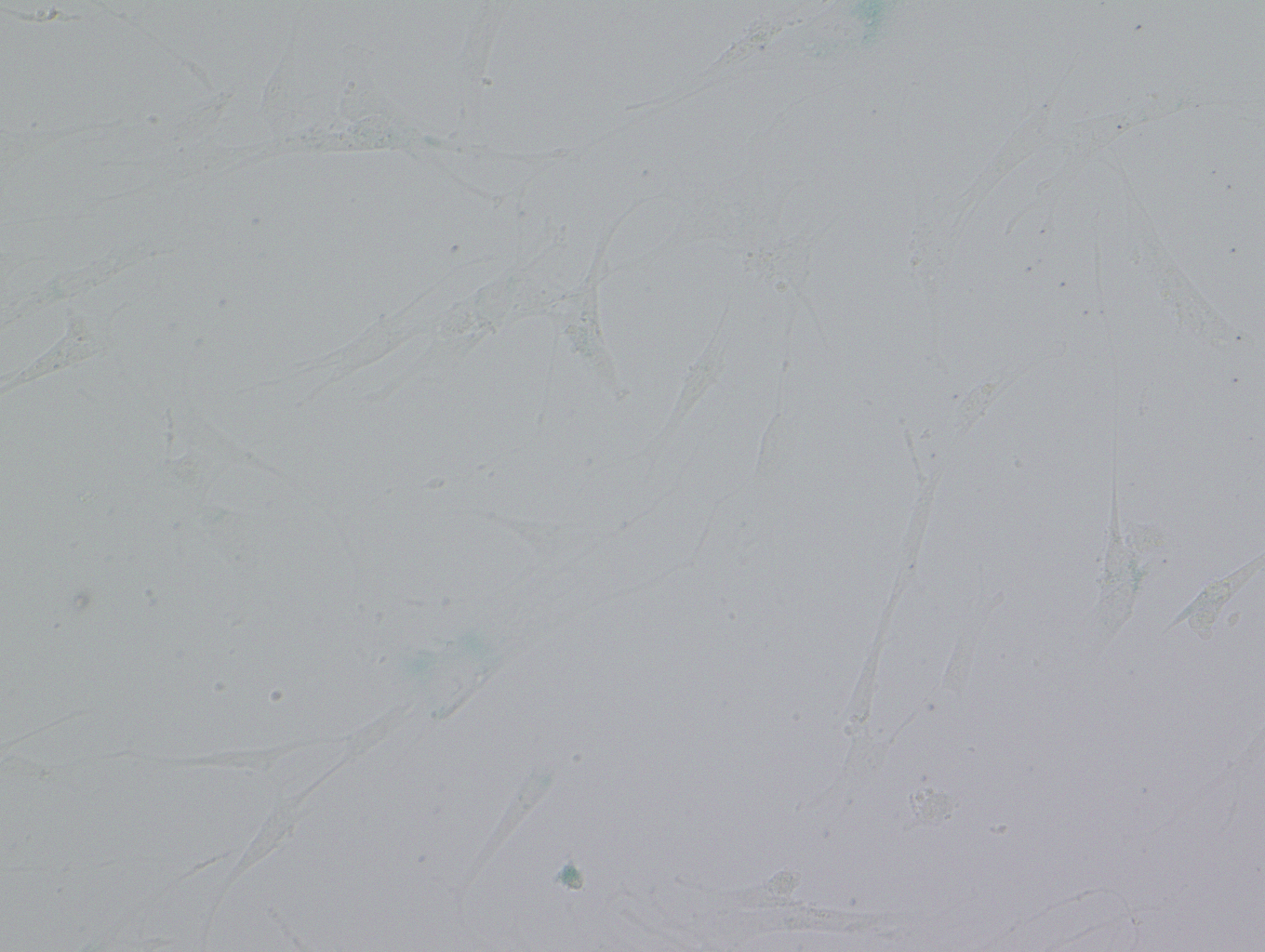

Supplement: Supplementary file 8 — EV and Appendix Figure Source Data [file 44318_2024_163_MOESM8_ESM.zip › Source Data for Expanded View and Appendix/Appendix Figure S3/S3A/betaGal WI38 DMSO.tif]

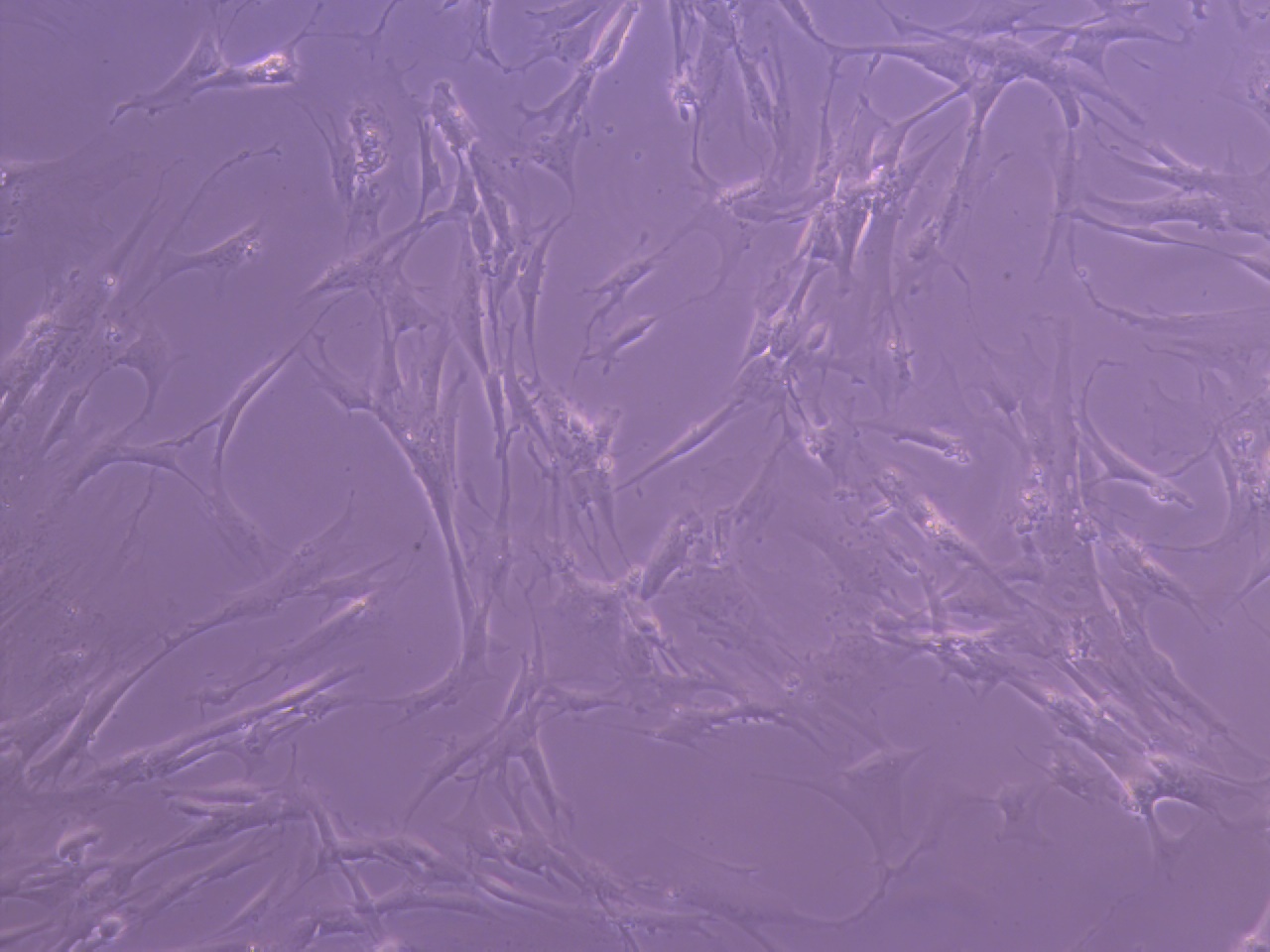

Supplement: Supplementary file 8 — EV and Appendix Figure Source Data [file 44318_2024_163_MOESM8_ESM.zip › Source Data for Expanded View and Appendix/Appendix Figure S3/S3A/Microscopy brighfield WI38 DMSO.jpg]

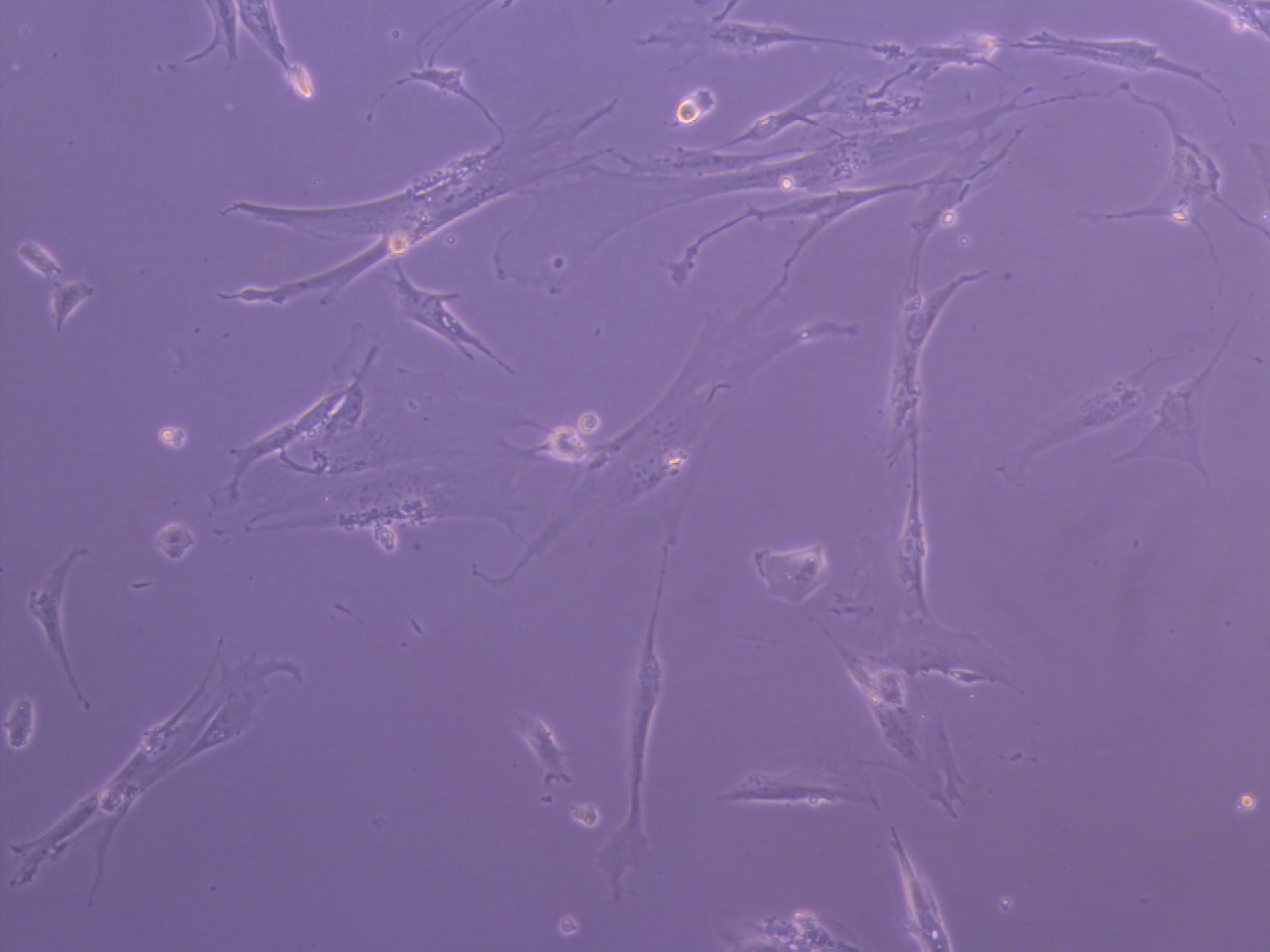

Supplement: Supplementary file 8 — EV and Appendix Figure Source Data [file 44318_2024_163_MOESM8_ESM.zip › Source Data for Expanded View and Appendix/Appendix Figure S3/S3A/Microscopy brightfield WI38 Auxin.jpg]

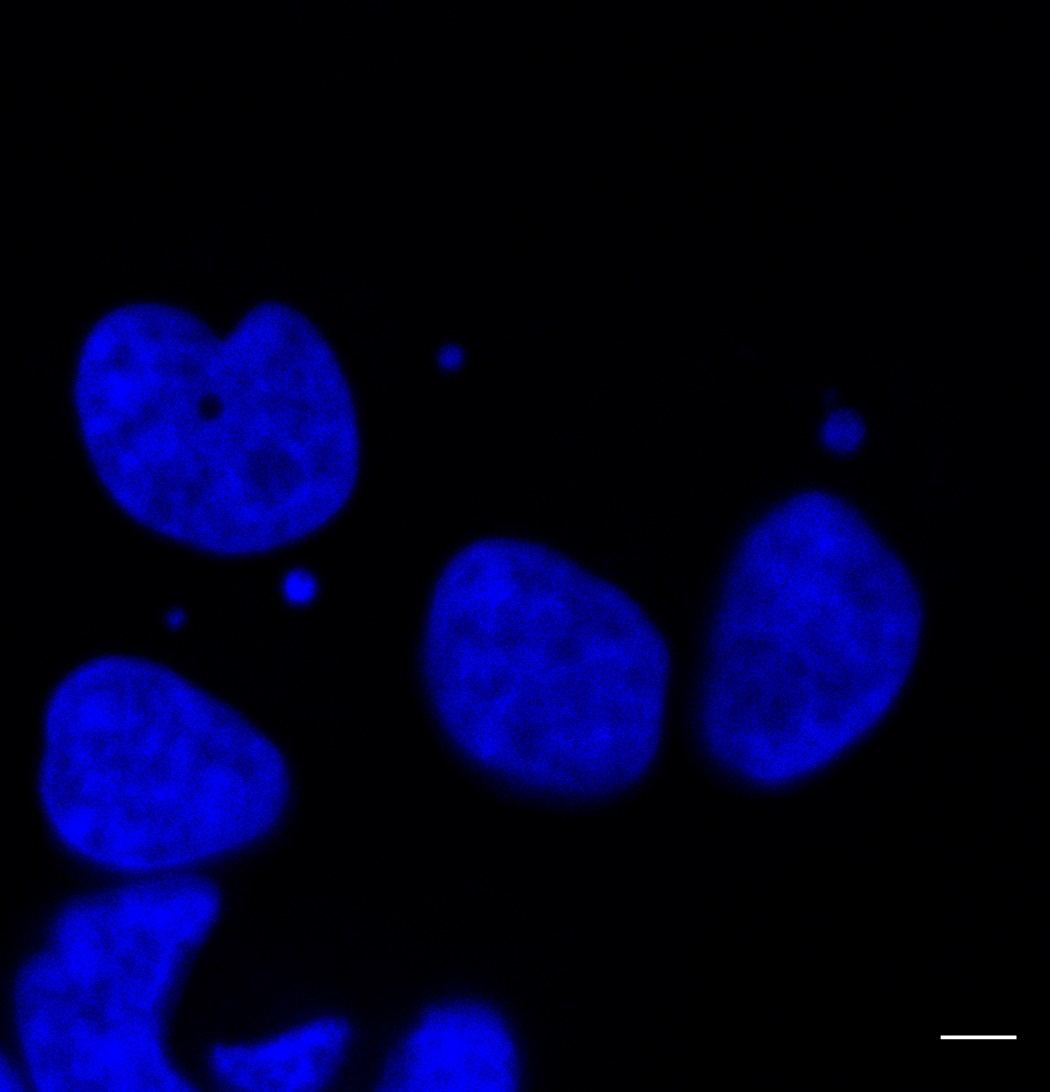

Supplement: Supplementary file 8 — EV and Appendix Figure Source Data [file 44318_2024_163_MOESM8_ESM.zip › Source Data for Expanded View and Appendix/Appendix Figure S3/S3B/Microscopy H3K27 DAPI pic1.jpg]

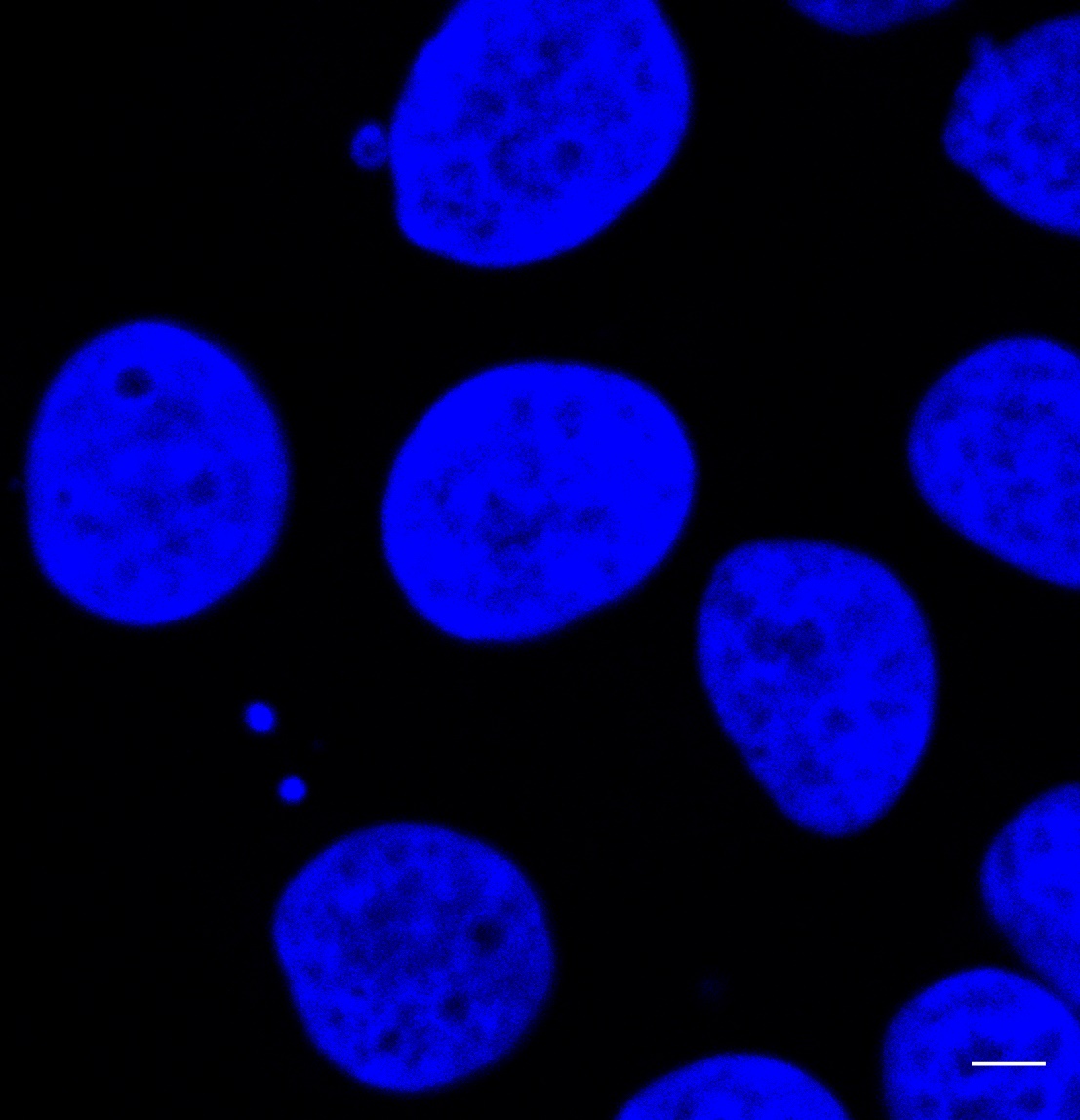

Supplement: Supplementary file 8 — EV and Appendix Figure Source Data [file 44318_2024_163_MOESM8_ESM.zip › Source Data for Expanded View and Appendix/Appendix Figure S3/S3B/Microscopy H3K27 DAPI pic2.jpg]

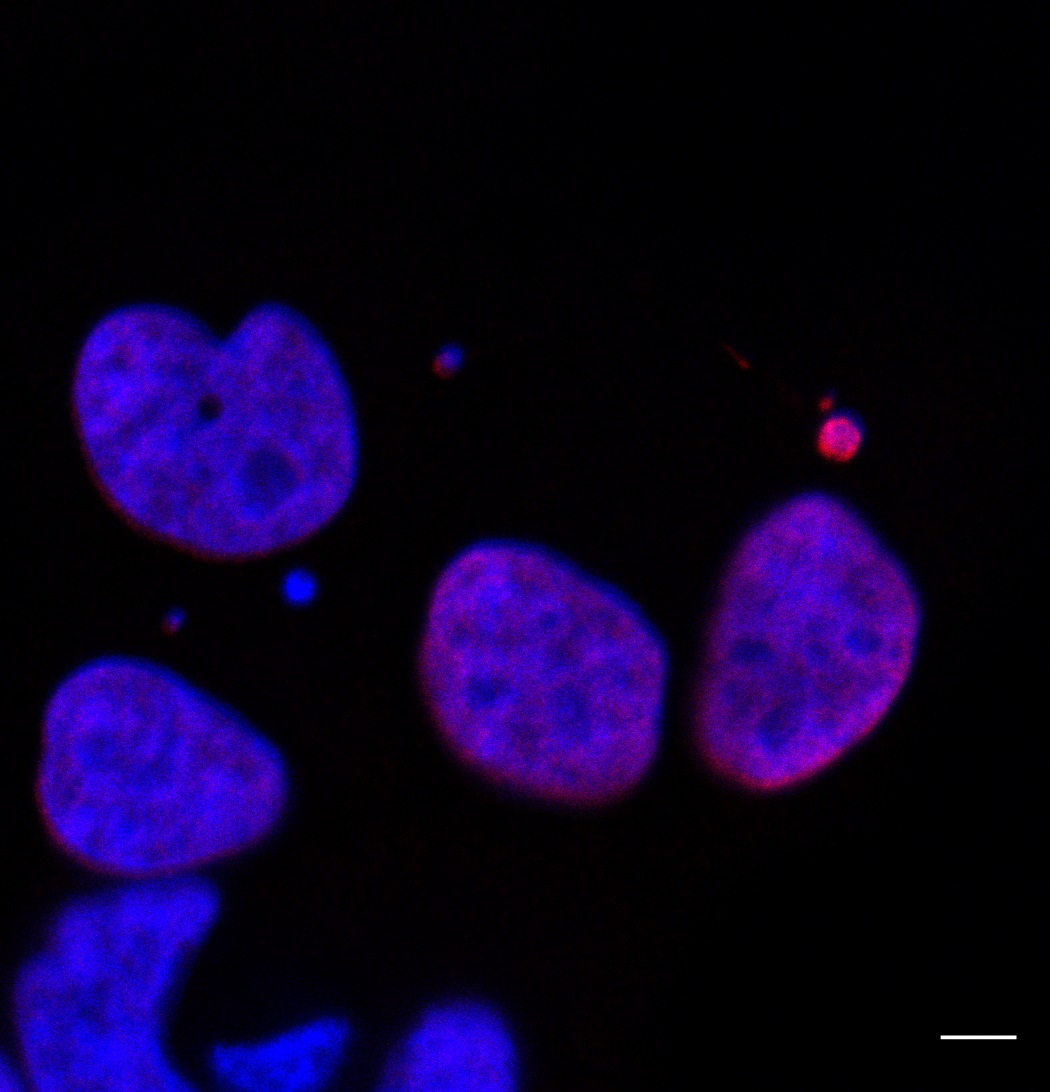

Supplement: Supplementary file 8 — EV and Appendix Figure Source Data [file 44318_2024_163_MOESM8_ESM.zip › Source Data for Expanded View and Appendix/Appendix Figure S3/S3B/Microscopy H3K27 merged pic1.jpg]

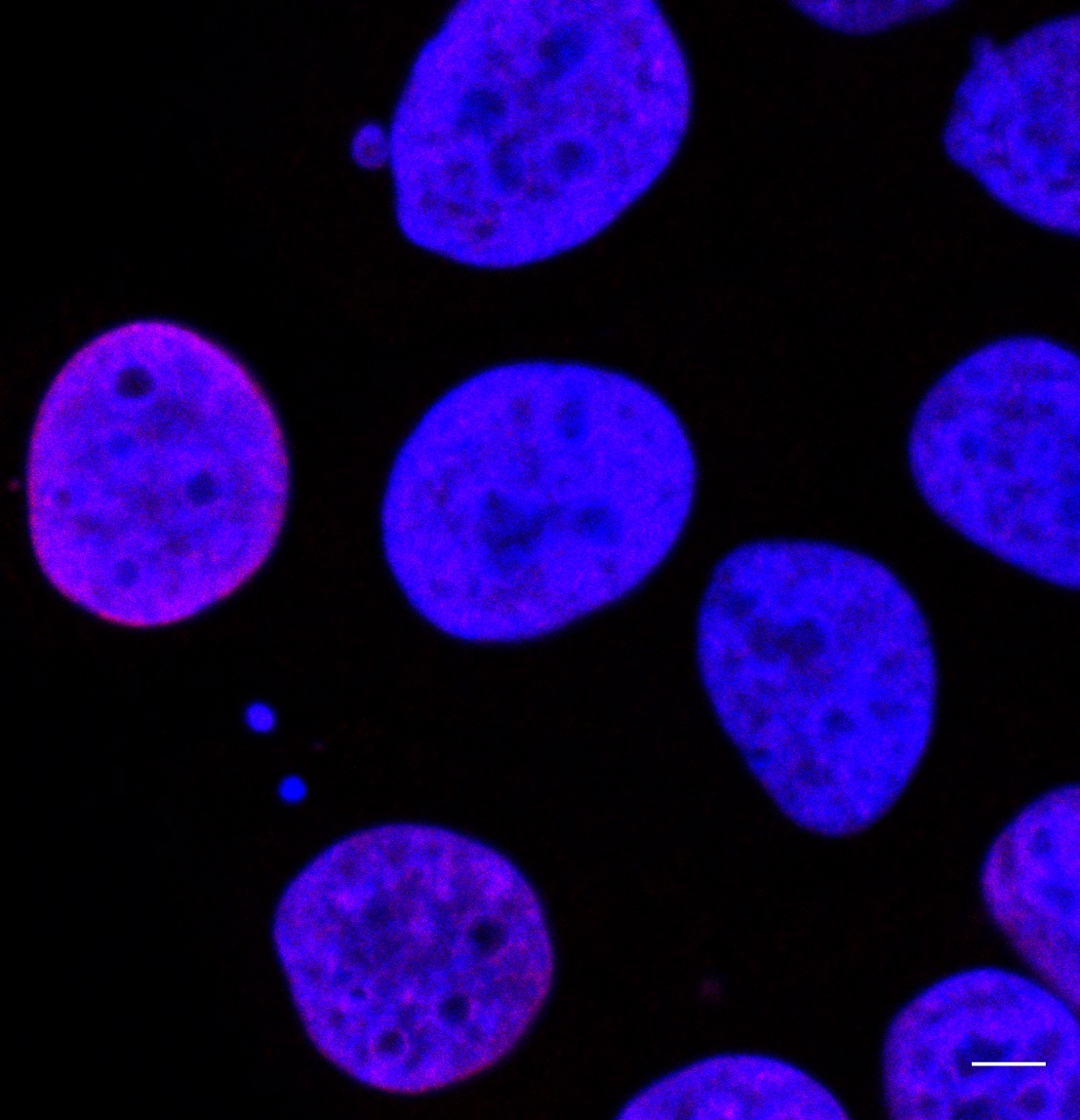

Supplement: Supplementary file 8 — EV and Appendix Figure Source Data [file 44318_2024_163_MOESM8_ESM.zip › Source Data for Expanded View and Appendix/Appendix Figure S3/S3B/Microscopy H3K27 merged pic2.jpg]

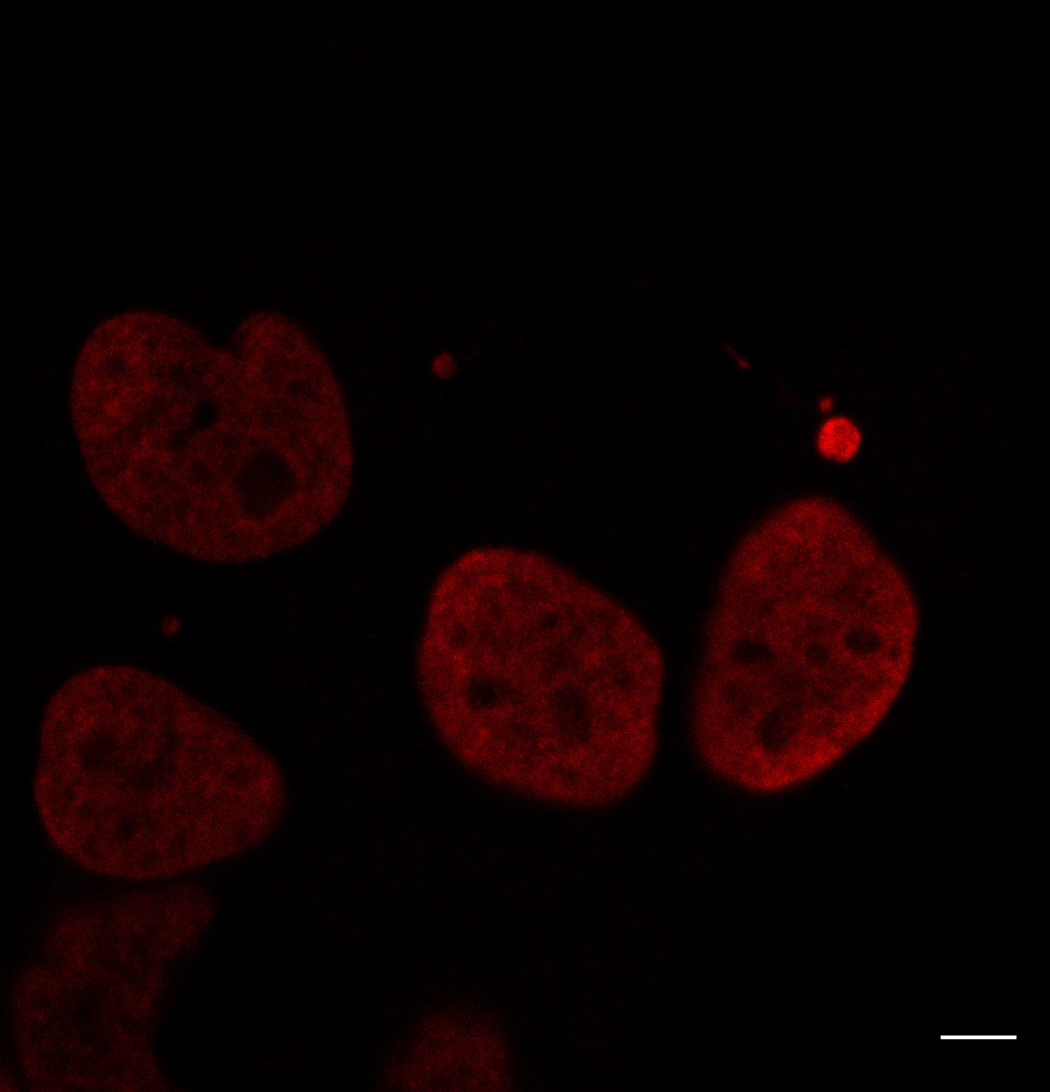

Supplement: Supplementary file 8 — EV and Appendix Figure Source Data [file 44318_2024_163_MOESM8_ESM.zip › Source Data for Expanded View and Appendix/Appendix Figure S3/S3B/Microscopy H3K27 pic1.jpg]

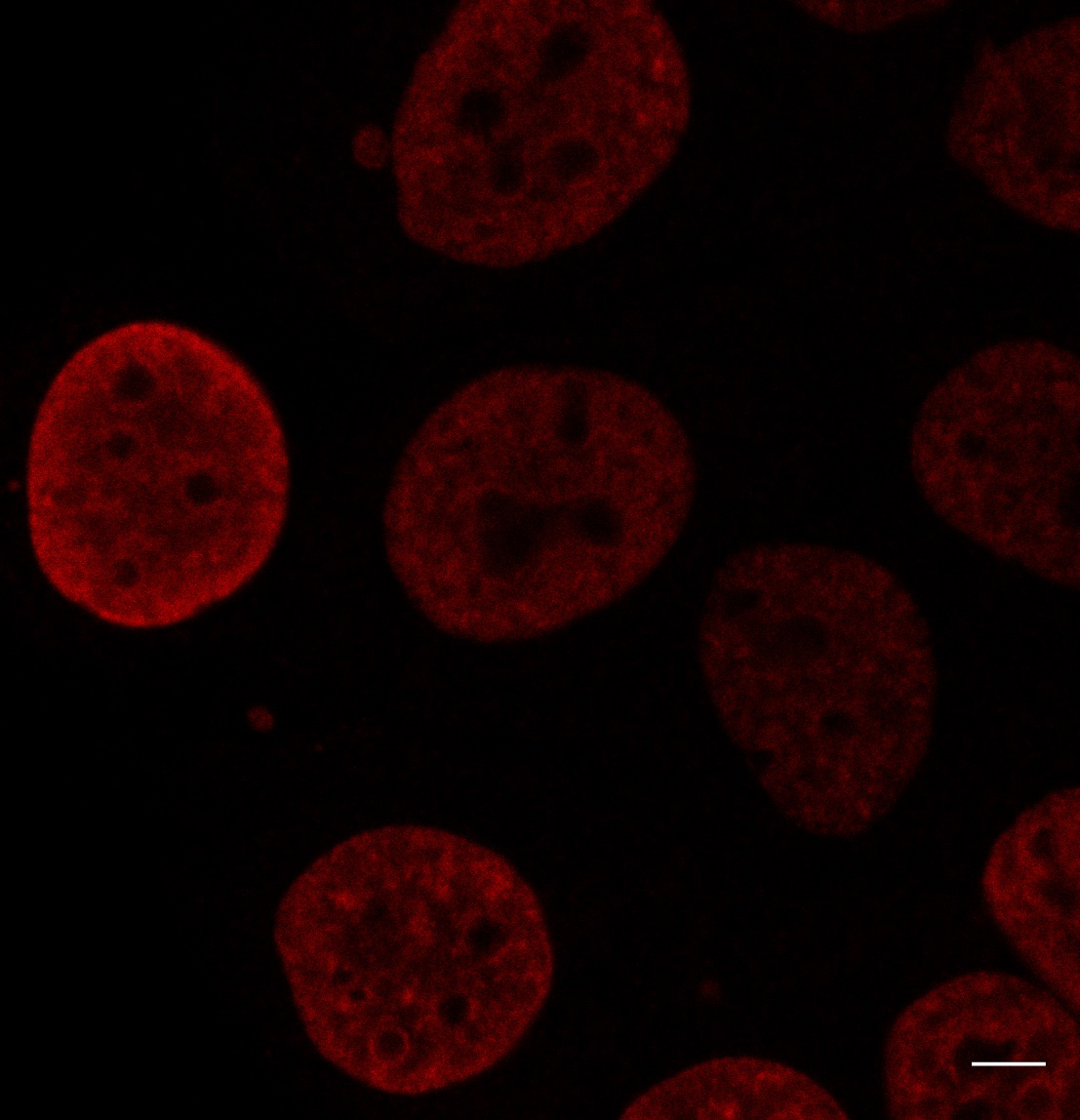

Supplement: Supplementary file 8 — EV and Appendix Figure Source Data [file 44318_2024_163_MOESM8_ESM.zip › Source Data for Expanded View and Appendix/Appendix Figure S3/S3B/Microscopy H3K27 pic2.jpg]

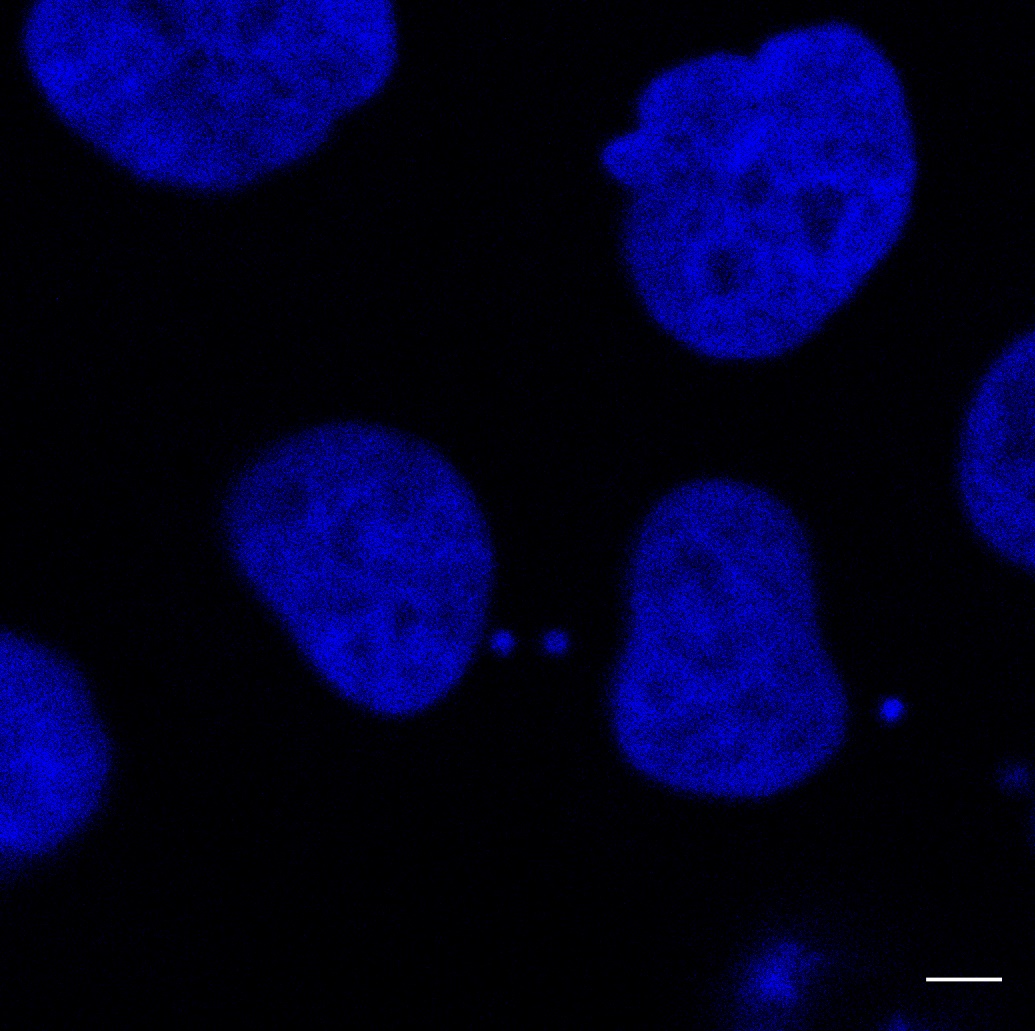

Supplement: Supplementary file 8 — EV and Appendix Figure Source Data [file 44318_2024_163_MOESM8_ESM.zip › Source Data for Expanded View and Appendix/Appendix Figure S3/S3B/Microscopy H3K9 DAPI pic1.jpg]

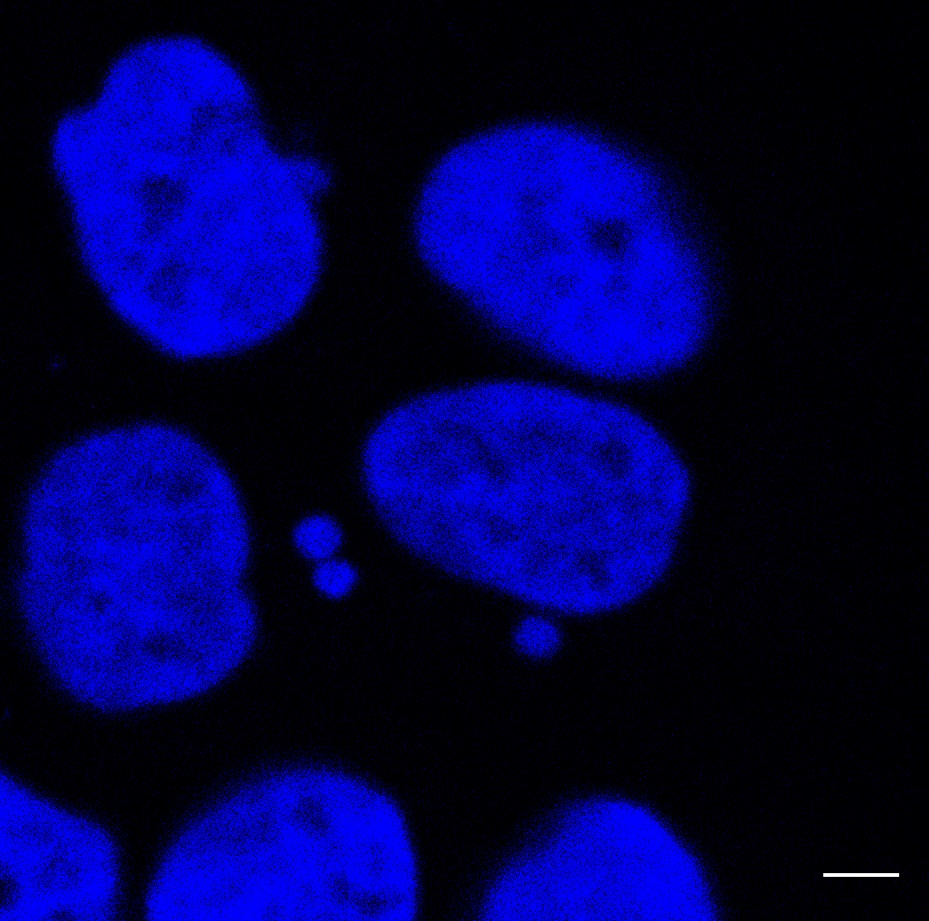

Supplement: Supplementary file 8 — EV and Appendix Figure Source Data [file 44318_2024_163_MOESM8_ESM.zip › Source Data for Expanded View and Appendix/Appendix Figure S3/S3B/Microscopy H3K9 DAPI pic2.jpg]

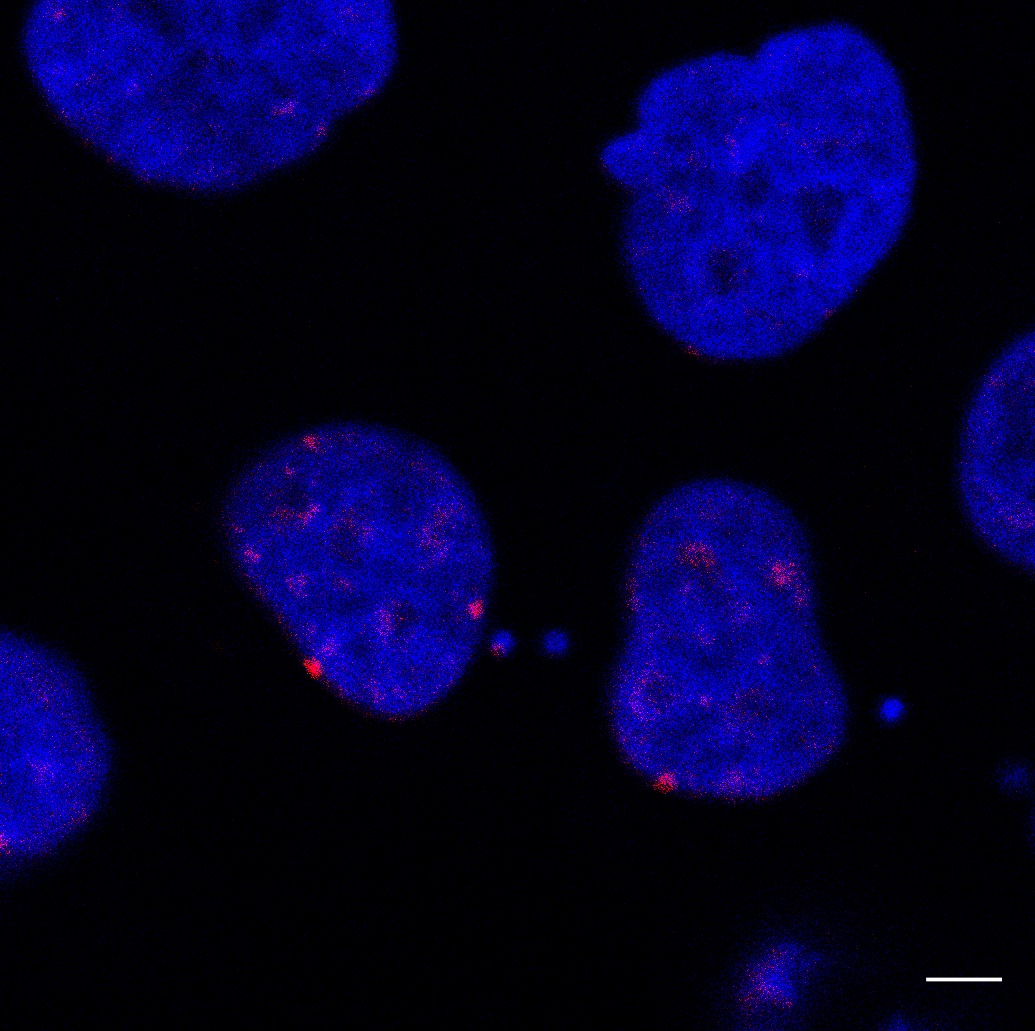

Supplement: Supplementary file 8 — EV and Appendix Figure Source Data [file 44318_2024_163_MOESM8_ESM.zip › Source Data for Expanded View and Appendix/Appendix Figure S3/S3B/Microscopy H3K9 merged pic1.jpg]

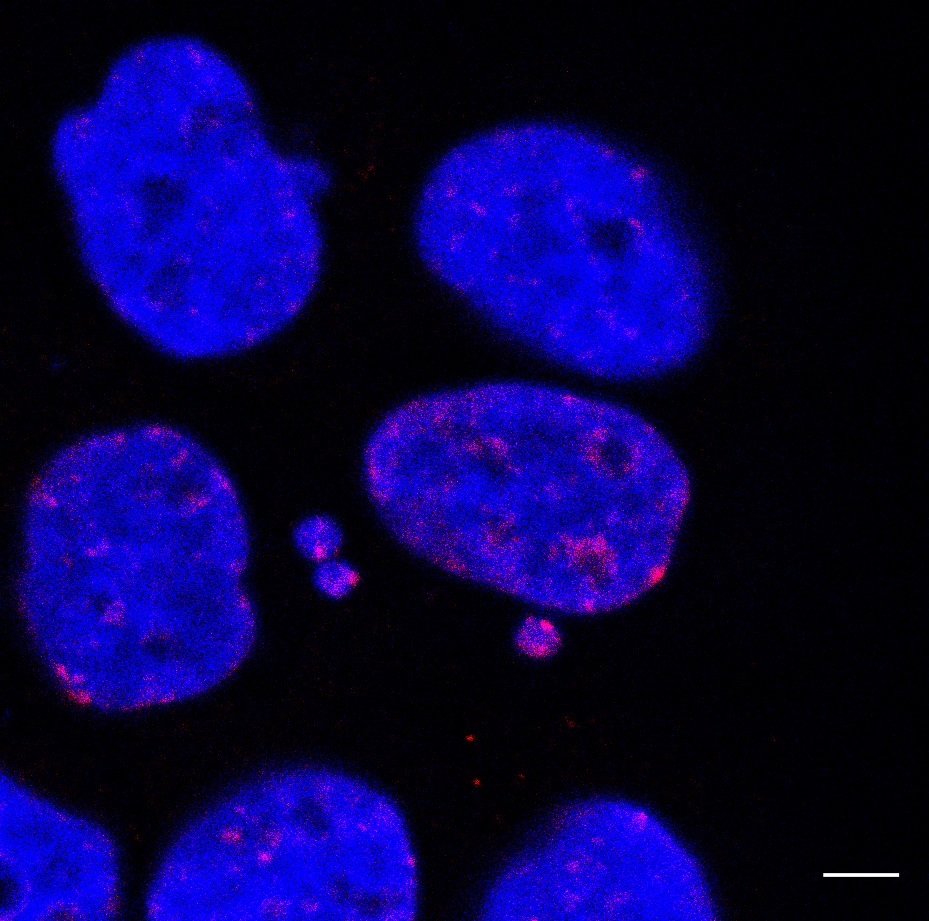

Supplement: Supplementary file 8 — EV and Appendix Figure Source Data [file 44318_2024_163_MOESM8_ESM.zip › Source Data for Expanded View and Appendix/Appendix Figure S3/S3B/Microscopy H3K9 merged pic2.jpg]

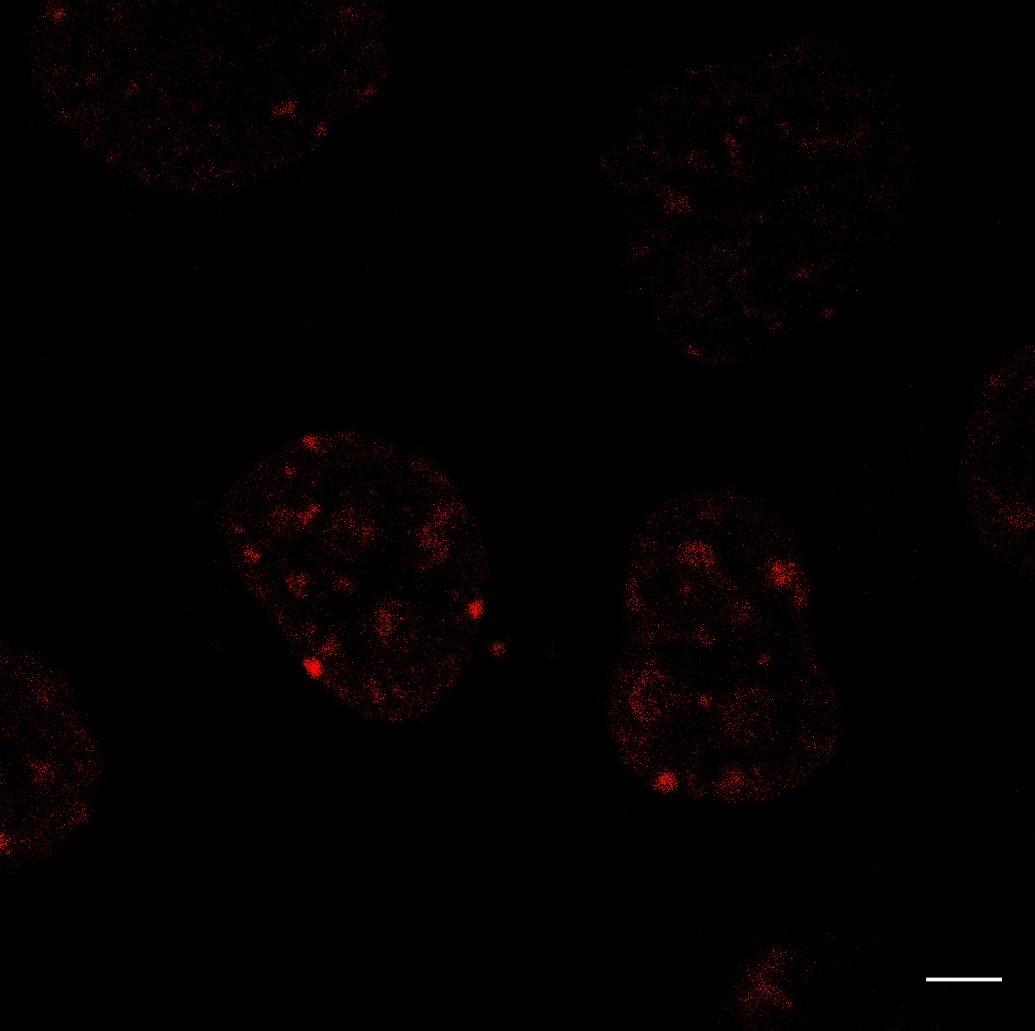

Supplement: Supplementary file 8 — EV and Appendix Figure Source Data [file 44318_2024_163_MOESM8_ESM.zip › Source Data for Expanded View and Appendix/Appendix Figure S3/S3B/Microscopy H3K9 pic1.jpg]

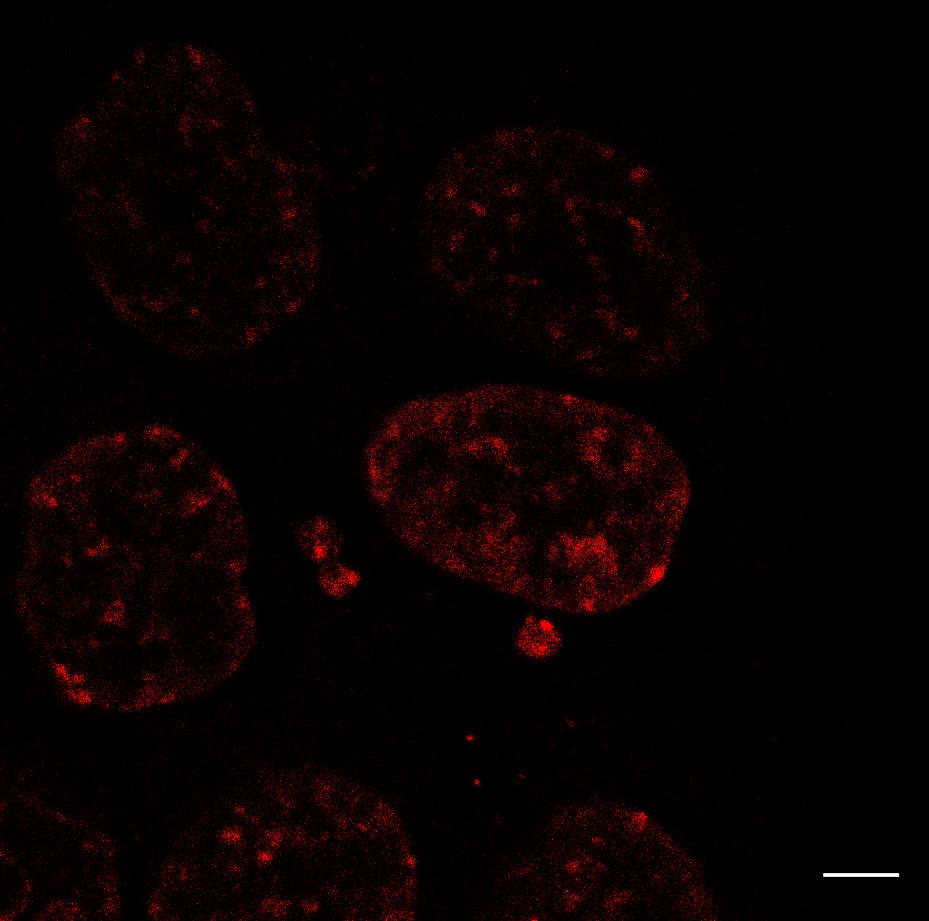

Supplement: Supplementary file 8 — EV and Appendix Figure Source Data [file 44318_2024_163_MOESM8_ESM.zip › Source Data for Expanded View and Appendix/Appendix Figure S3/S3B/Microscopy H3K9 pic2.jpg]

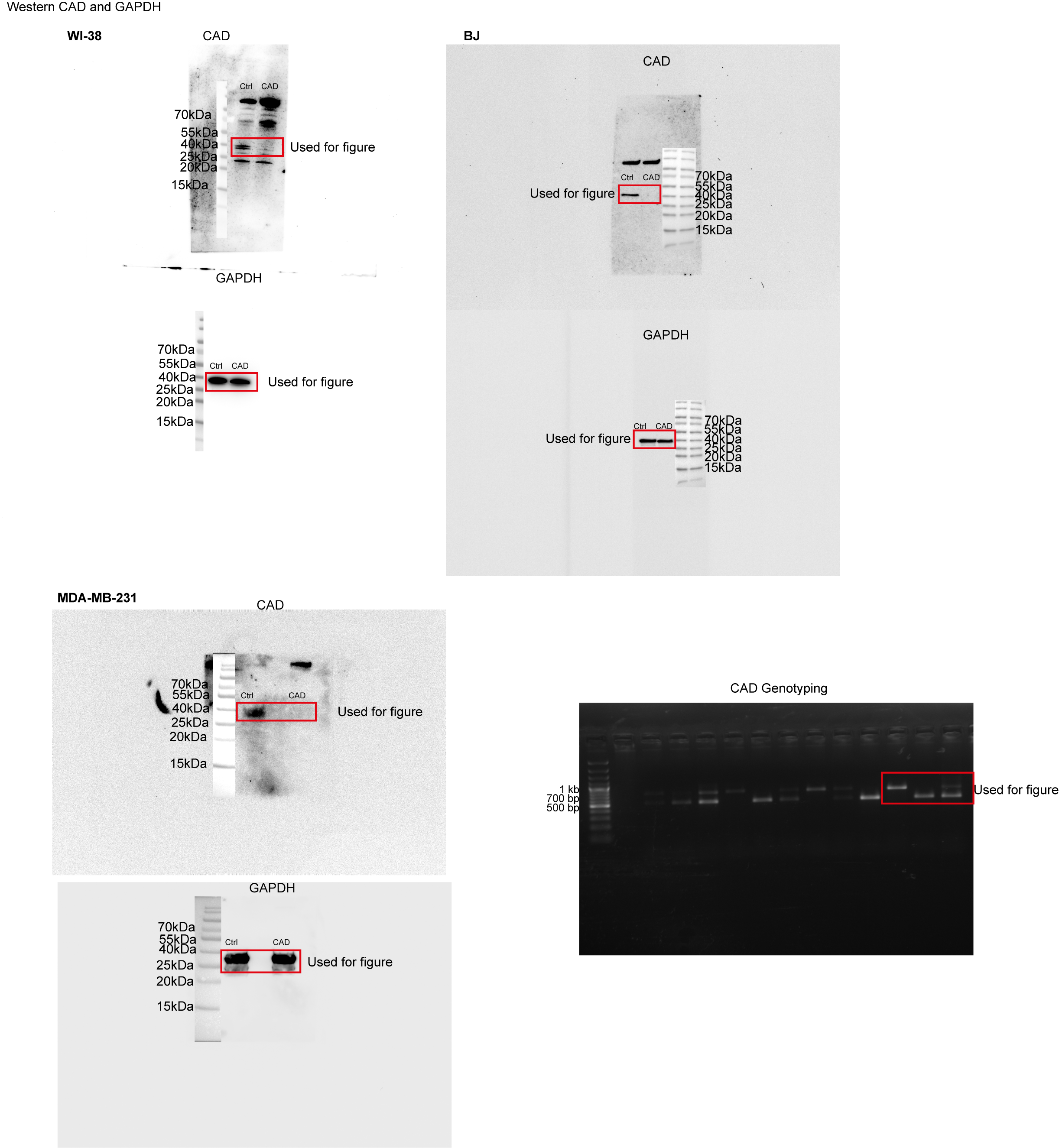

Supplement: Supplementary file 8 — EV and Appendix Figure Source Data [file 44318_2024_163_MOESM8_ESM.zip › Source Data for Expanded View and Appendix/EV1/1B/Western KO efficiency.tif]

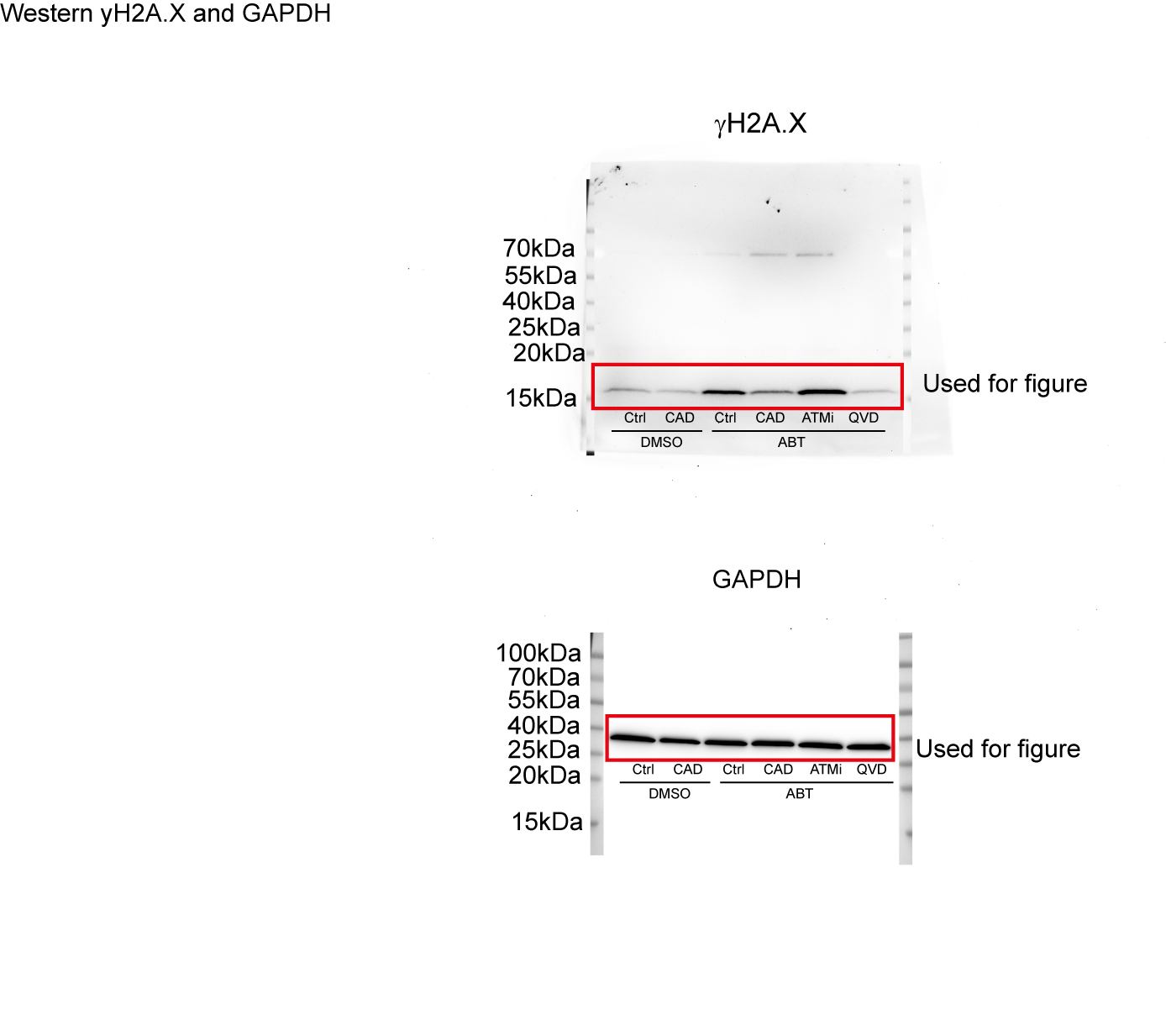

Supplement: Supplementary file 8 — EV and Appendix Figure Source Data [file 44318_2024_163_MOESM8_ESM.zip › Source Data for Expanded View and Appendix/EV1/1C/Western yH2AX GAPDH WI38.tif]

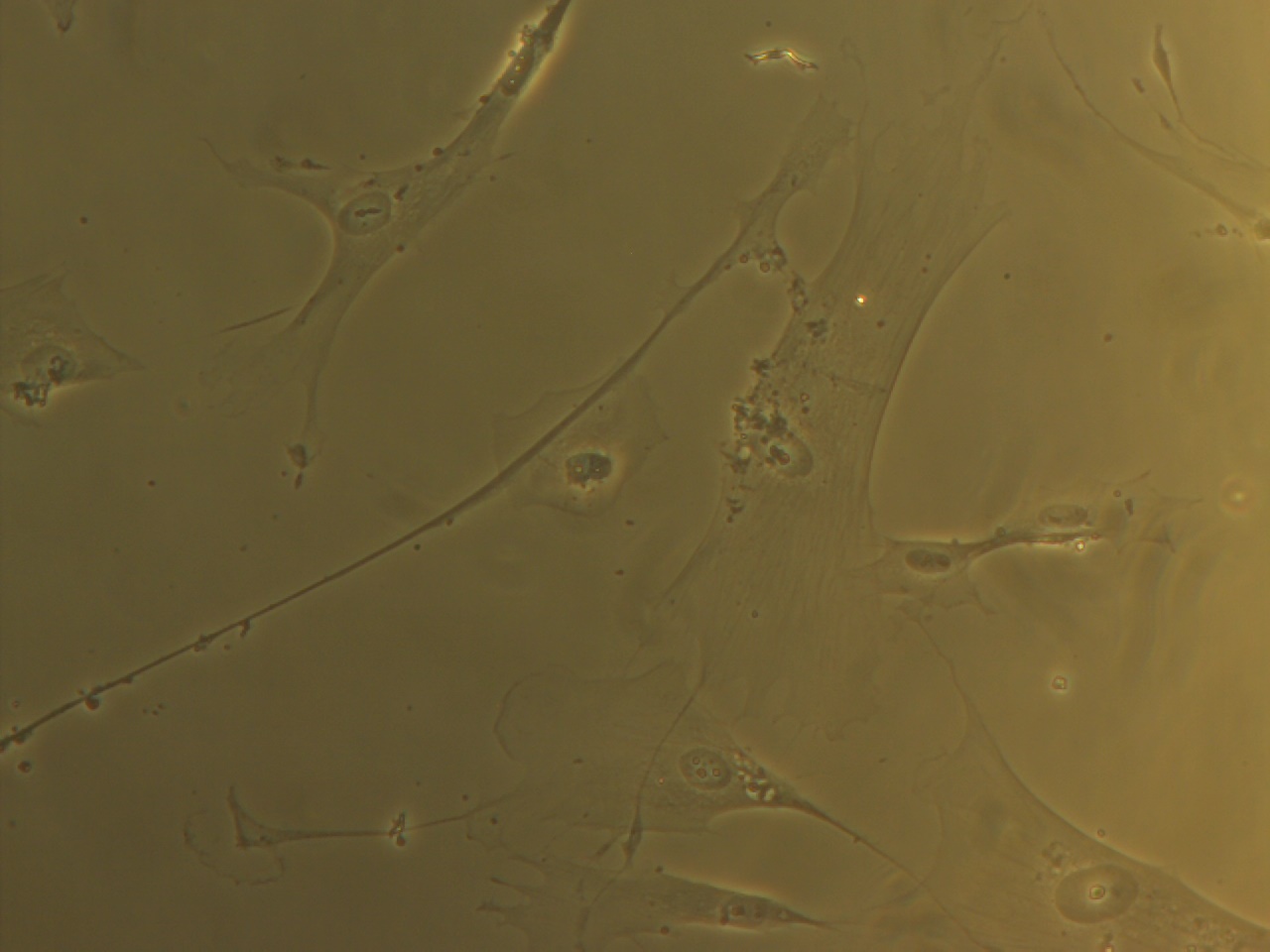

Supplement: Supplementary file 8 — EV and Appendix Figure Source Data [file 44318_2024_163_MOESM8_ESM.zip › Source Data for Expanded View and Appendix/EV2/2A/Microscopy BJ ABT.jpg]

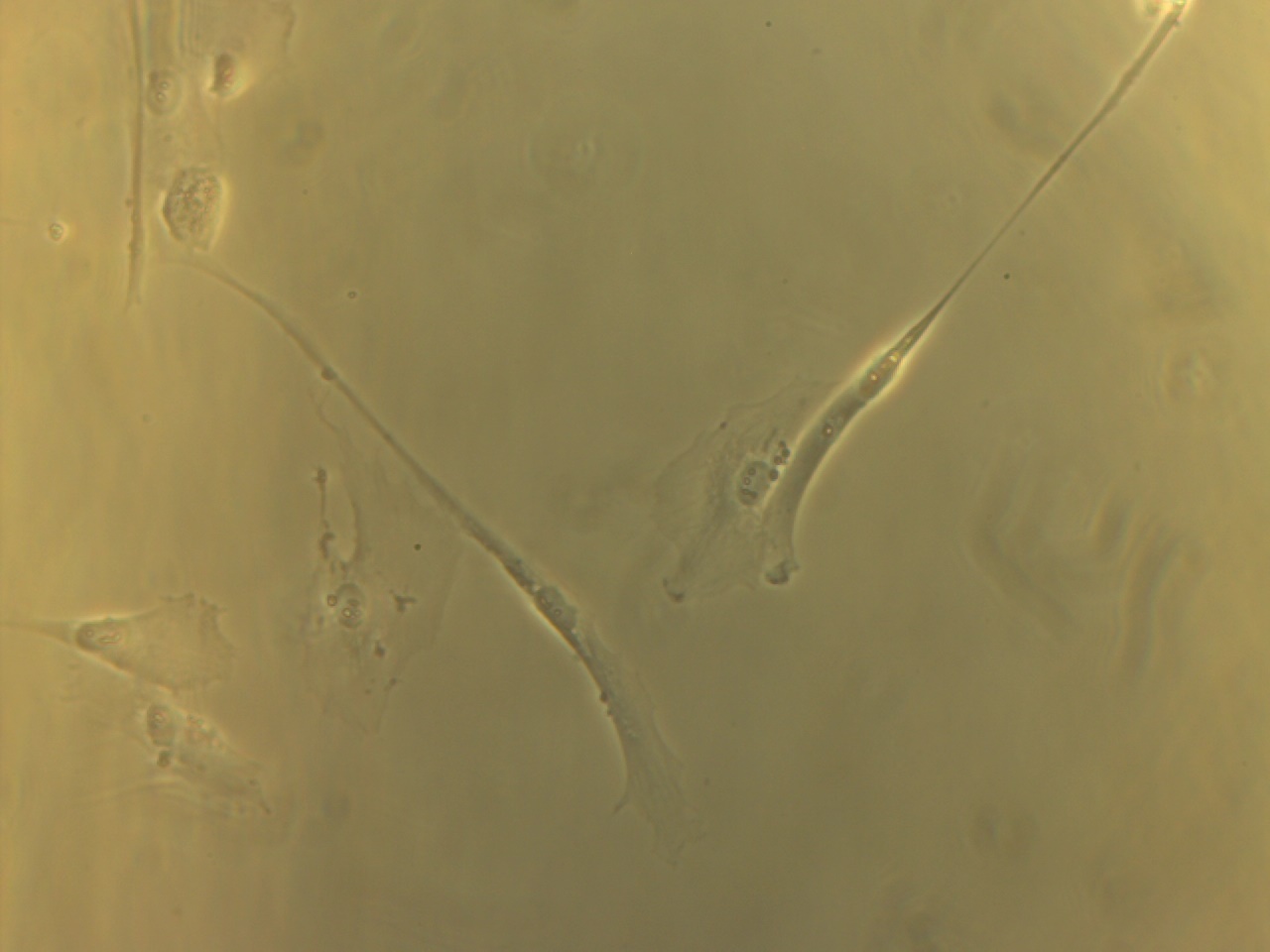

Supplement: Supplementary file 8 — EV and Appendix Figure Source Data [file 44318_2024_163_MOESM8_ESM.zip › Source Data for Expanded View and Appendix/EV2/2A/Microscopy BJ DMSO.jpg]

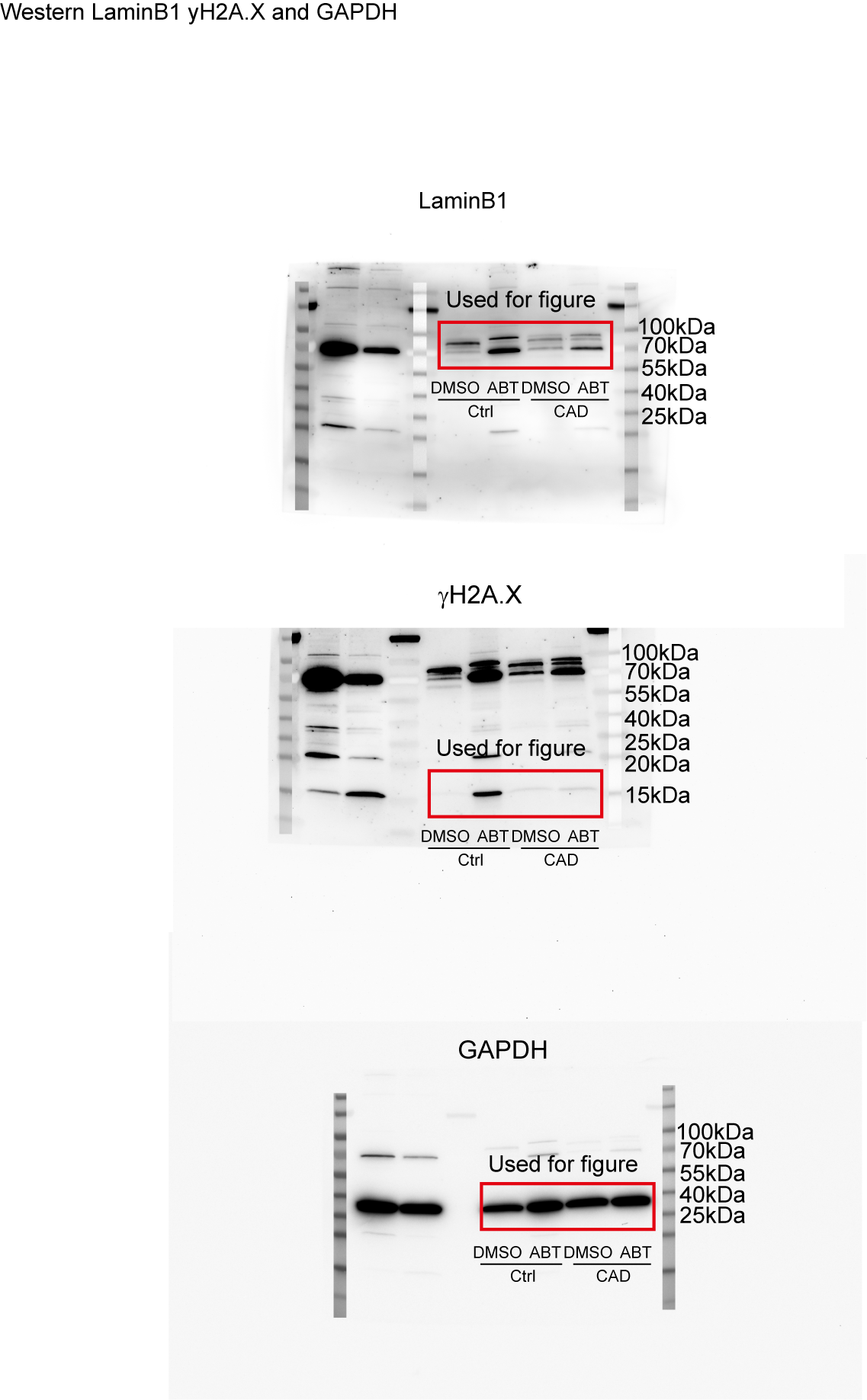

Supplement: Supplementary file 8 — EV and Appendix Figure Source Data [file 44318_2024_163_MOESM8_ESM.zip › Source Data for Expanded View and Appendix/EV2/2B/Western yH2AX LaminB1 GAPDH BJ ABT.tif]

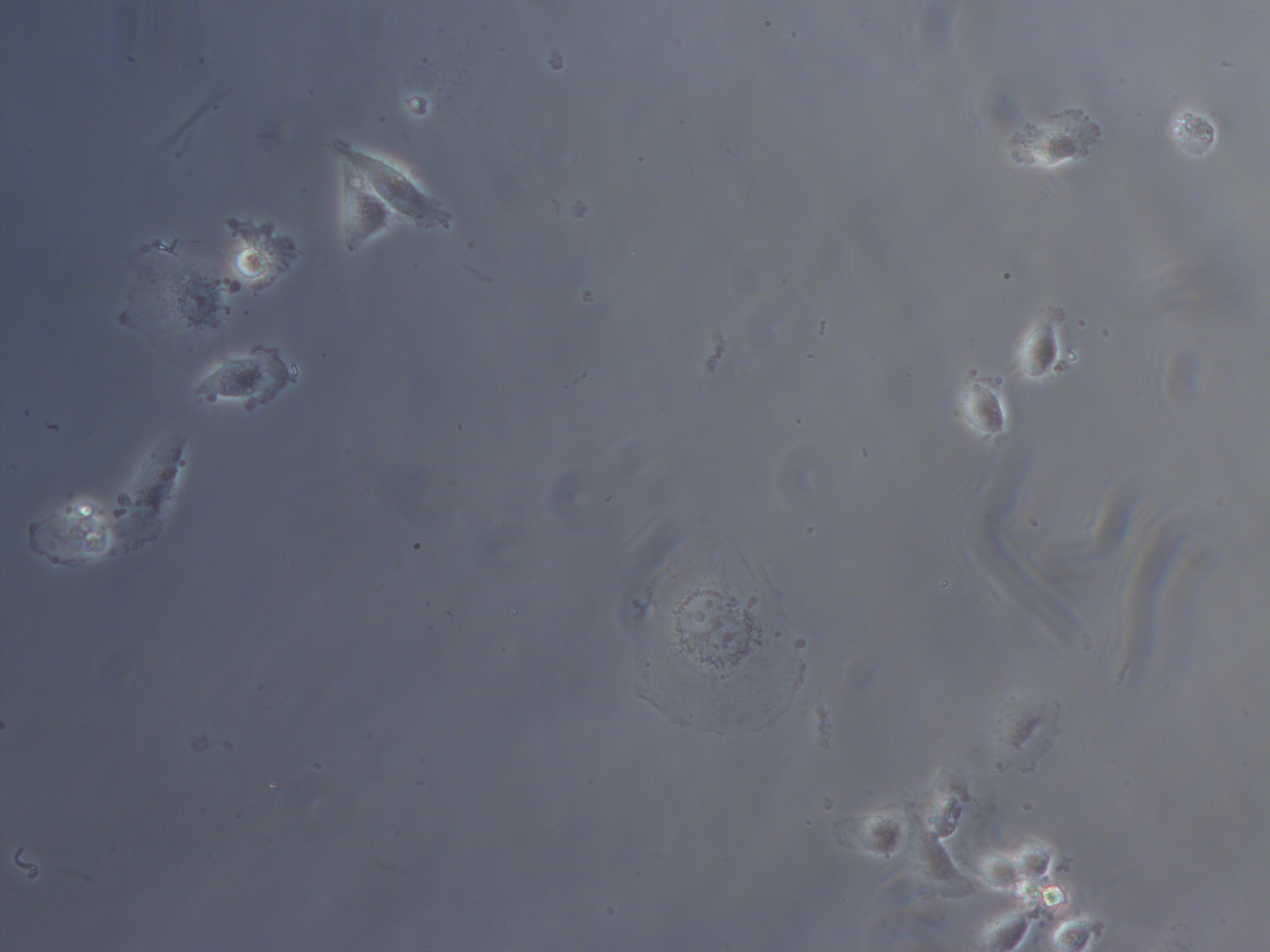

Supplement: Supplementary file 8 — EV and Appendix Figure Source Data [file 44318_2024_163_MOESM8_ESM.zip › Source Data for Expanded View and Appendix/EV2/2E/Microscopy MDA ABT.tif]

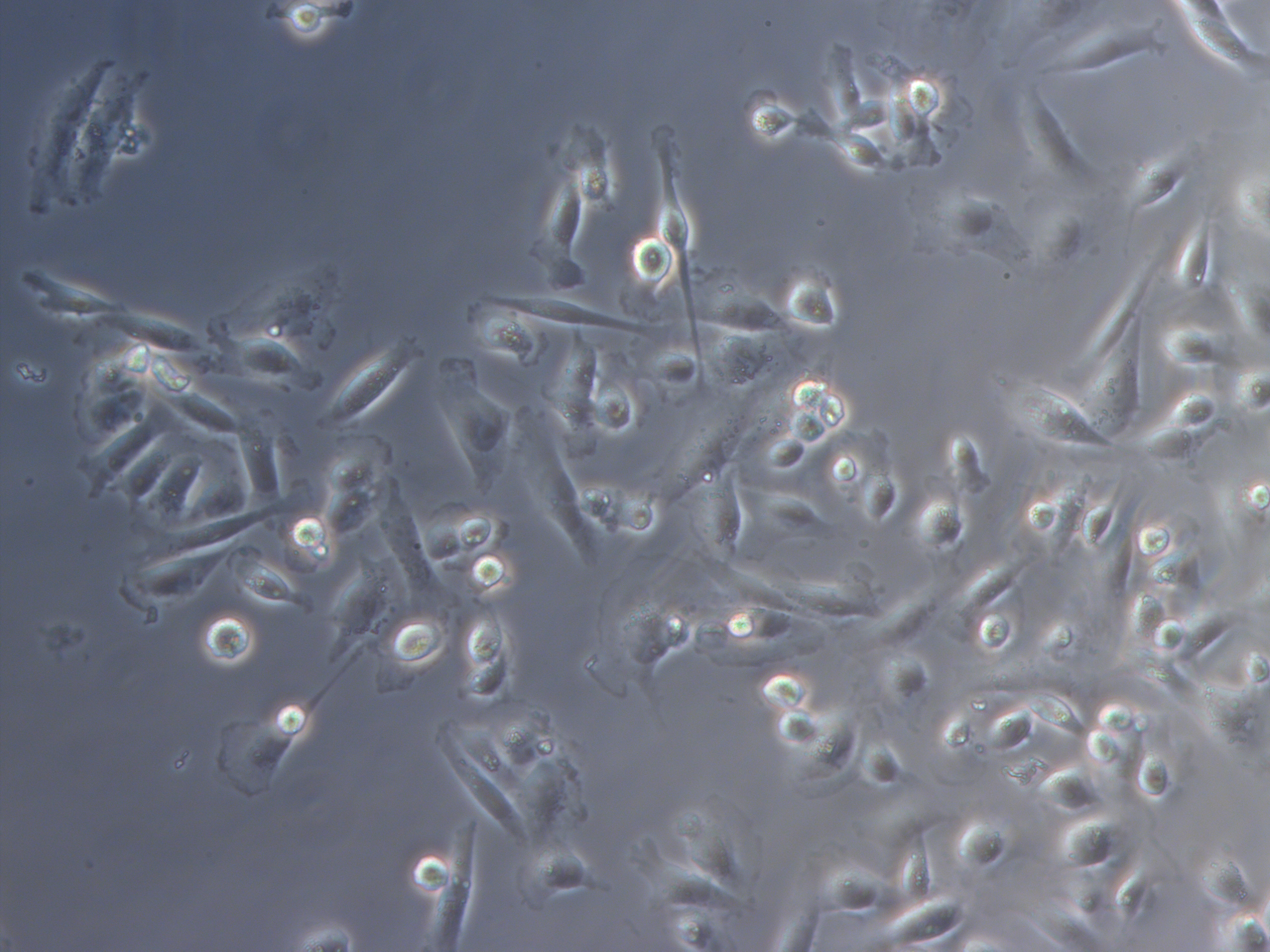

Supplement: Supplementary file 8 — EV and Appendix Figure Source Data [file 44318_2024_163_MOESM8_ESM.zip › Source Data for Expanded View and Appendix/EV2/2E/Microscopy MDA DMSO.tif]

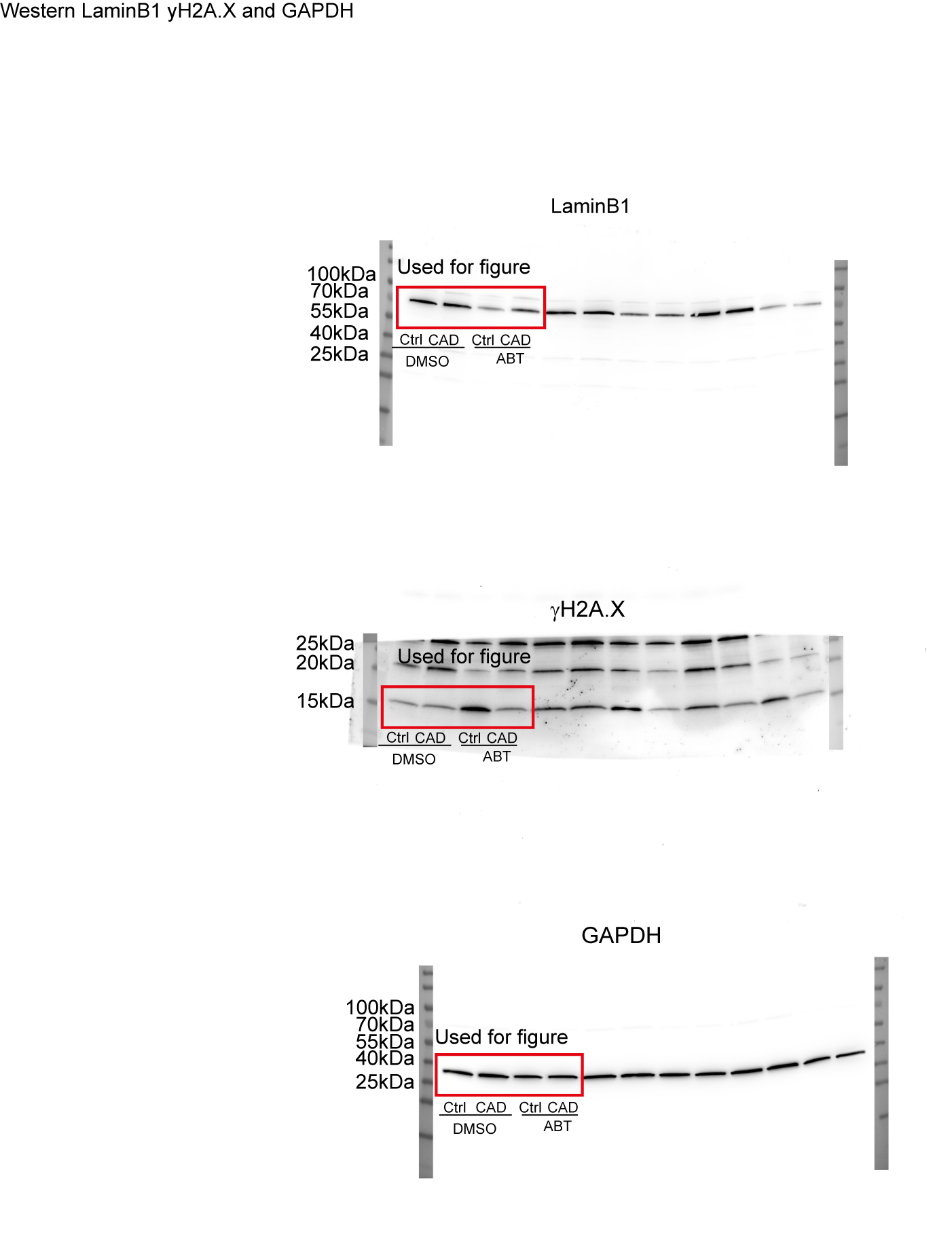

Supplement: Supplementary file 8 — EV and Appendix Figure Source Data [file 44318_2024_163_MOESM8_ESM.zip › Source Data for Expanded View and Appendix/EV2/2F/Western yH2AX LaminB1 GAPDH MDA ABT.tif]

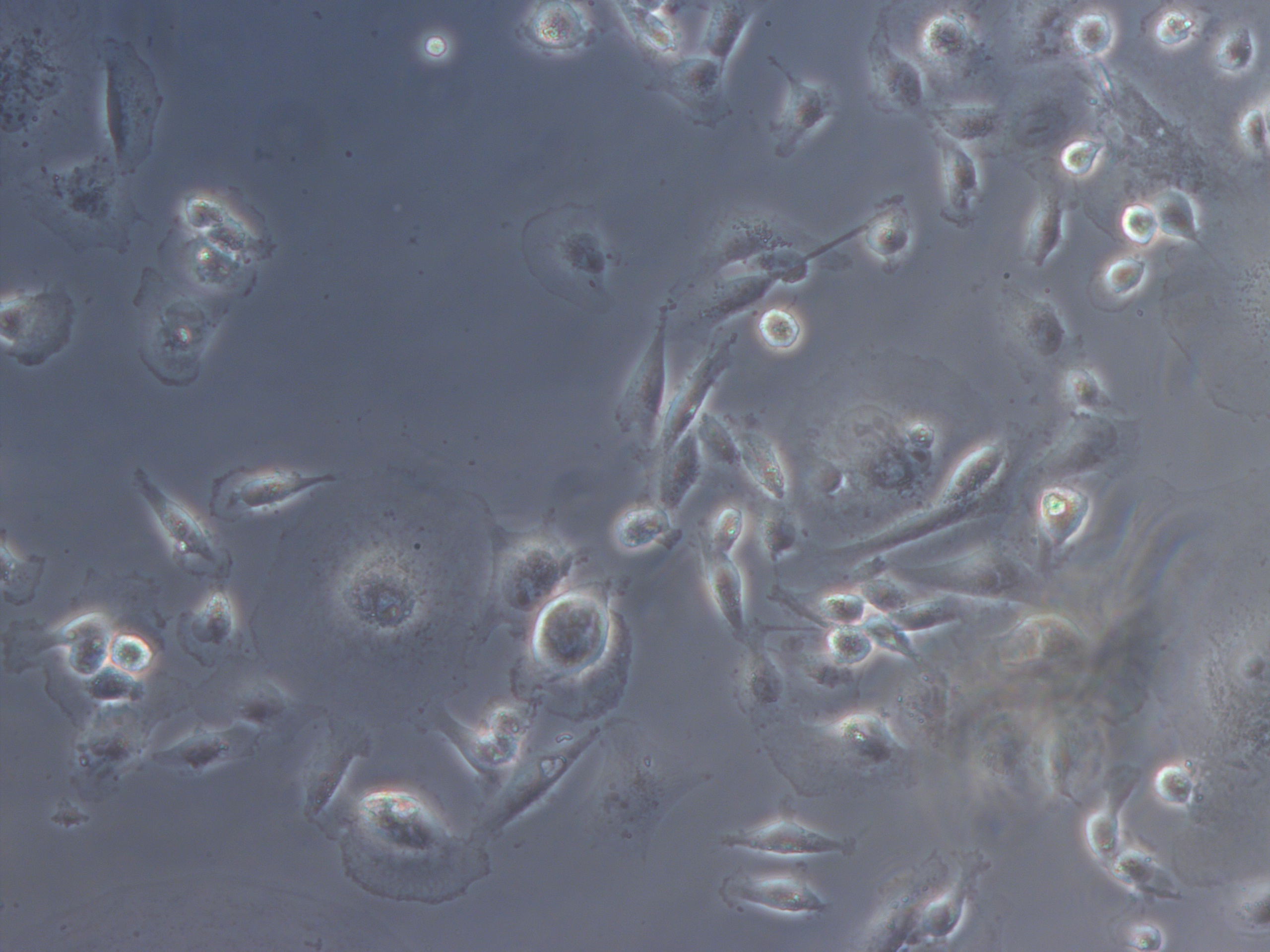

Supplement: Supplementary file 8 — EV and Appendix Figure Source Data [file 44318_2024_163_MOESM8_ESM.zip › Source Data for Expanded View and Appendix/EV3/3A/Microscopy MDA Doxo.jpg]

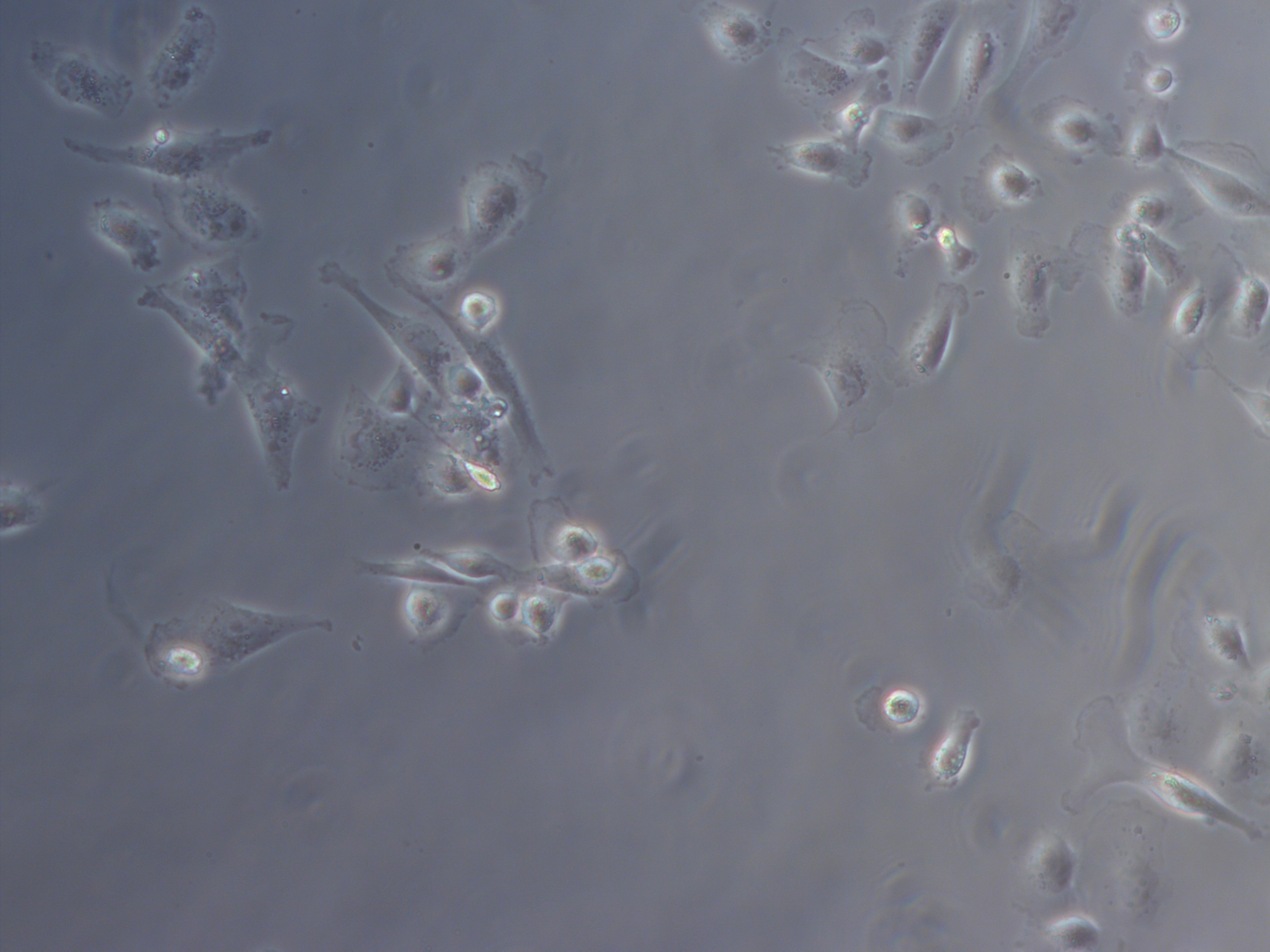

Supplement: Supplementary file 8 — EV and Appendix Figure Source Data [file 44318_2024_163_MOESM8_ESM.zip › Source Data for Expanded View and Appendix/EV3/3A/Microscopy MDA untreated.jpg]

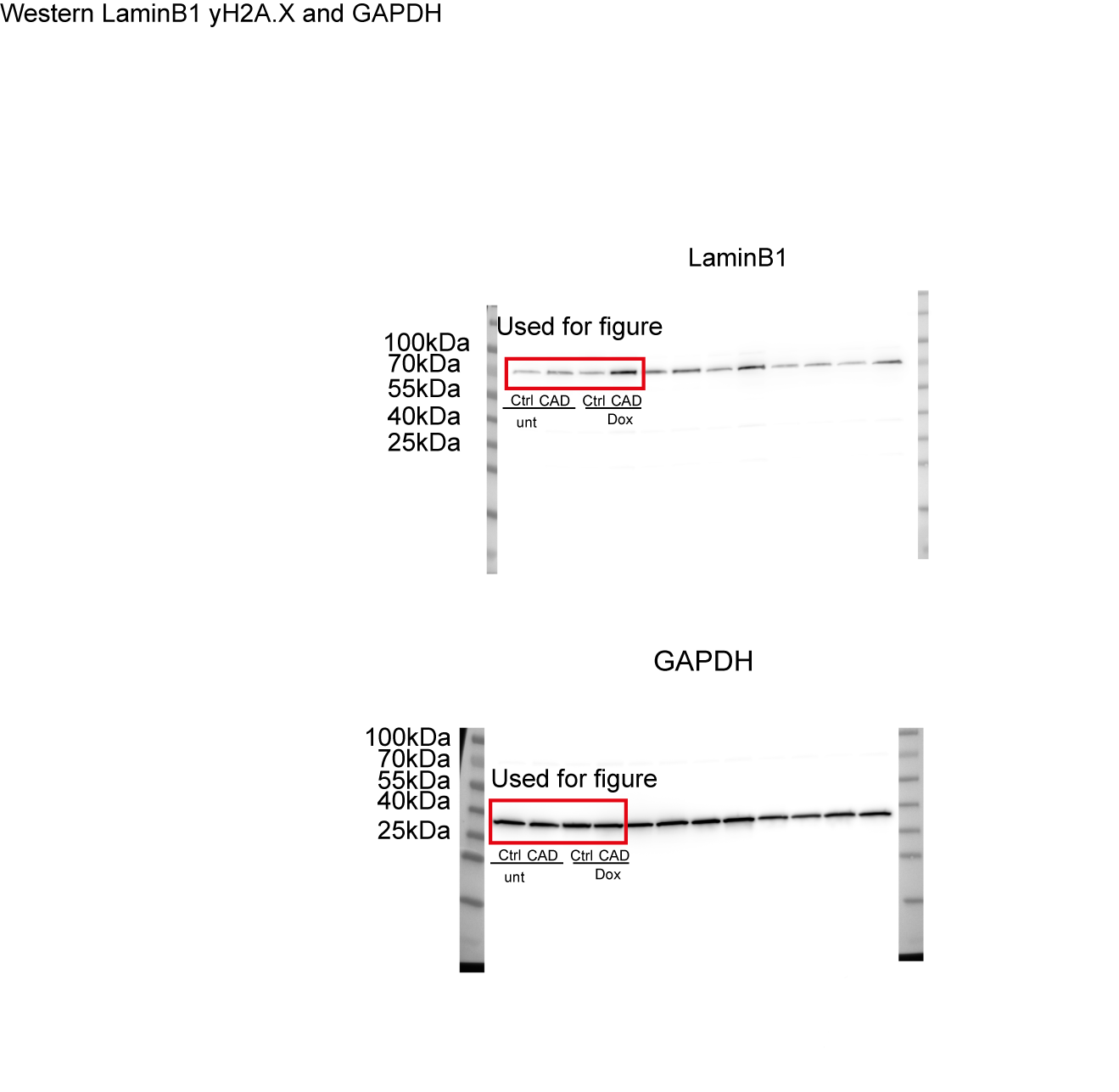

Supplement: Supplementary file 8 — EV and Appendix Figure Source Data [file 44318_2024_163_MOESM8_ESM.zip › Source Data for Expanded View and Appendix/EV3/3B/Western LaminB1 GAPDH MDA Doxo.tif]

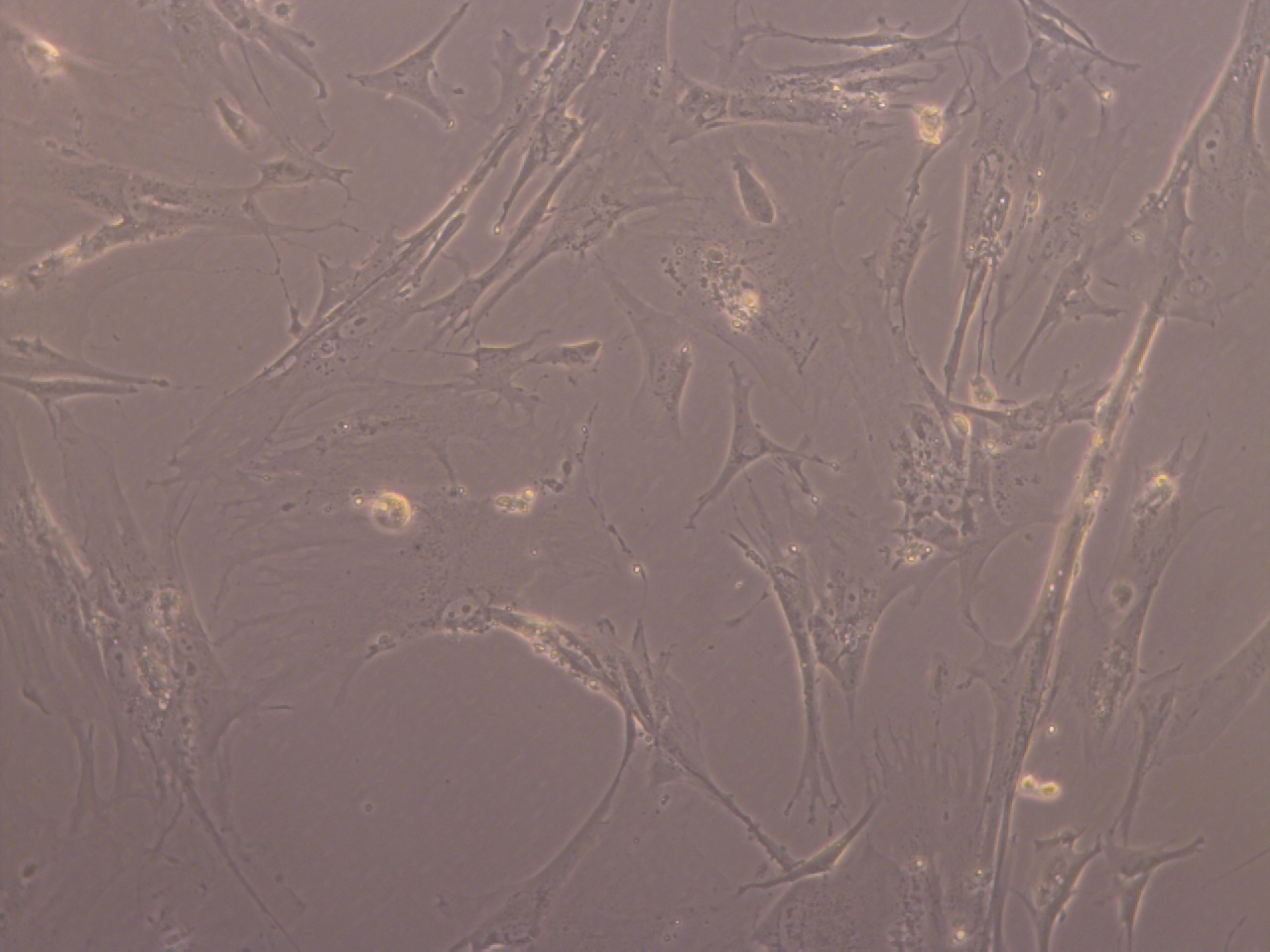

Supplement: Supplementary file 8 — EV and Appendix Figure Source Data [file 44318_2024_163_MOESM8_ESM.zip › Source Data for Expanded View and Appendix/EV3/3D/Microscopy WI38 Doxo.jpg]

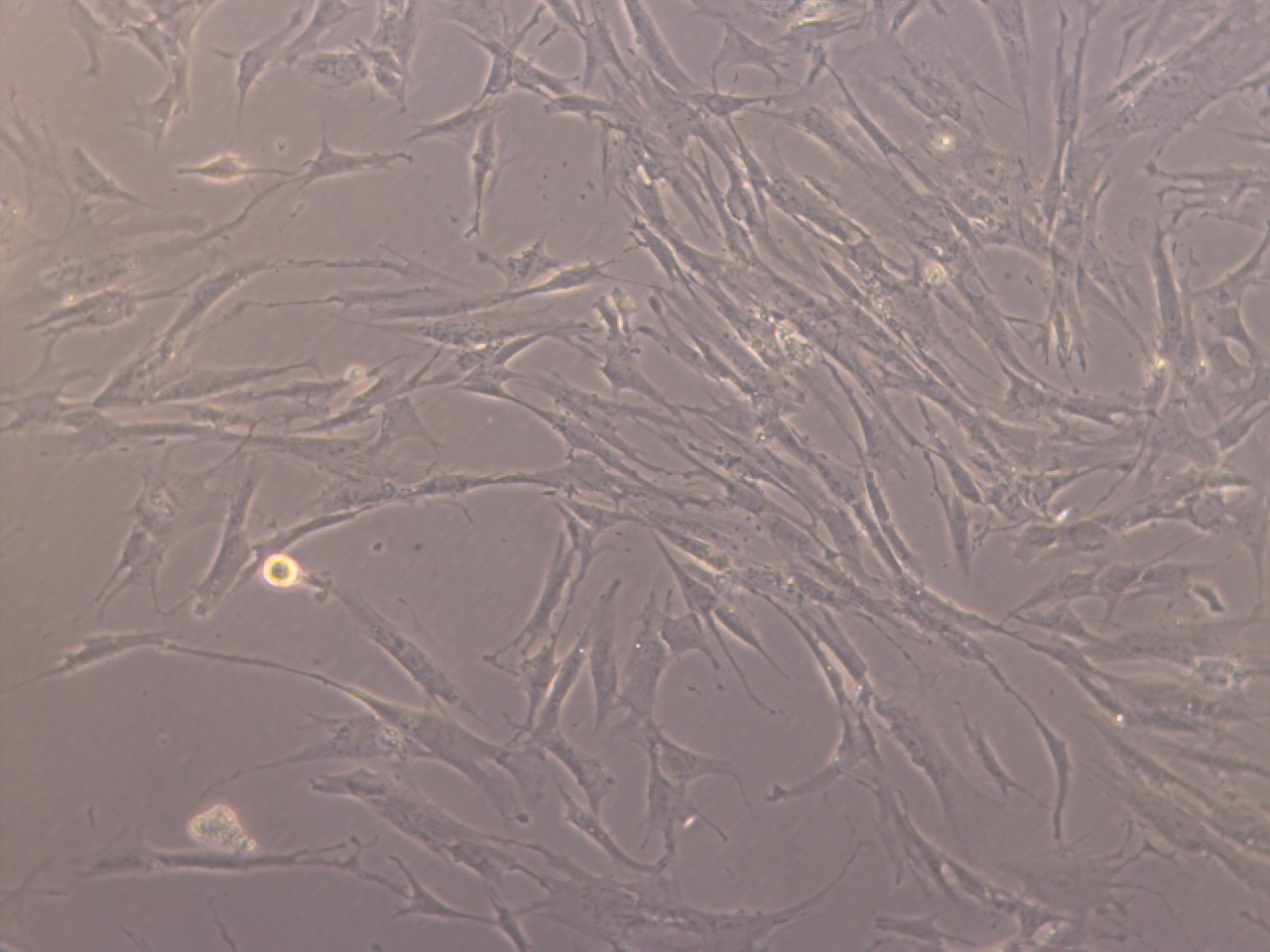

Supplement: Supplementary file 8 — EV and Appendix Figure Source Data [file 44318_2024_163_MOESM8_ESM.zip › Source Data for Expanded View and Appendix/EV3/3D/Microscopy WI38 untreated.jpg]

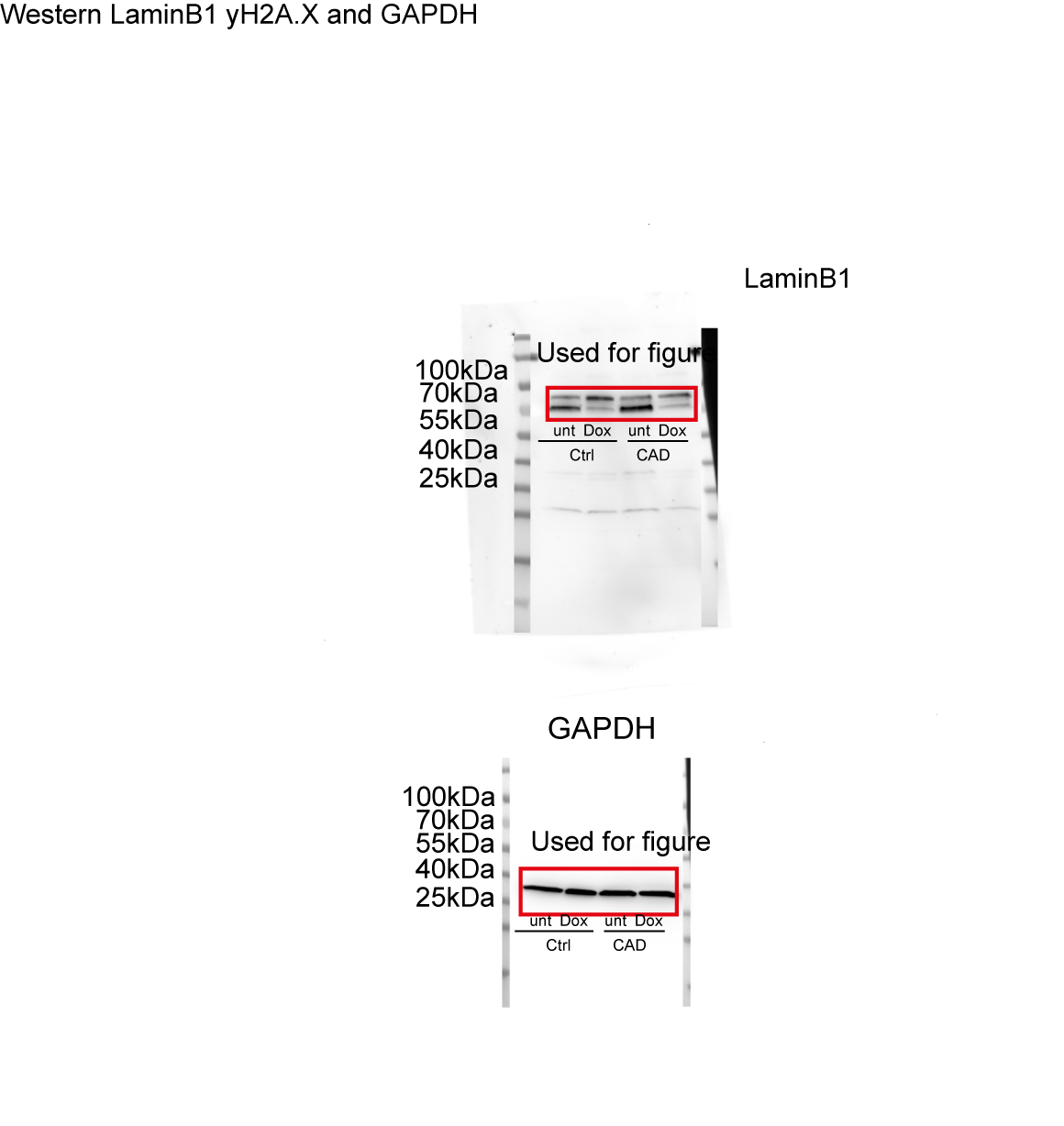

Supplement: Supplementary file 8 — EV and Appendix Figure Source Data [file 44318_2024_163_MOESM8_ESM.zip › Source Data for Expanded View and Appendix/EV3/3E/Western LaminB1 GAPDH WI38 Doxo.tif]

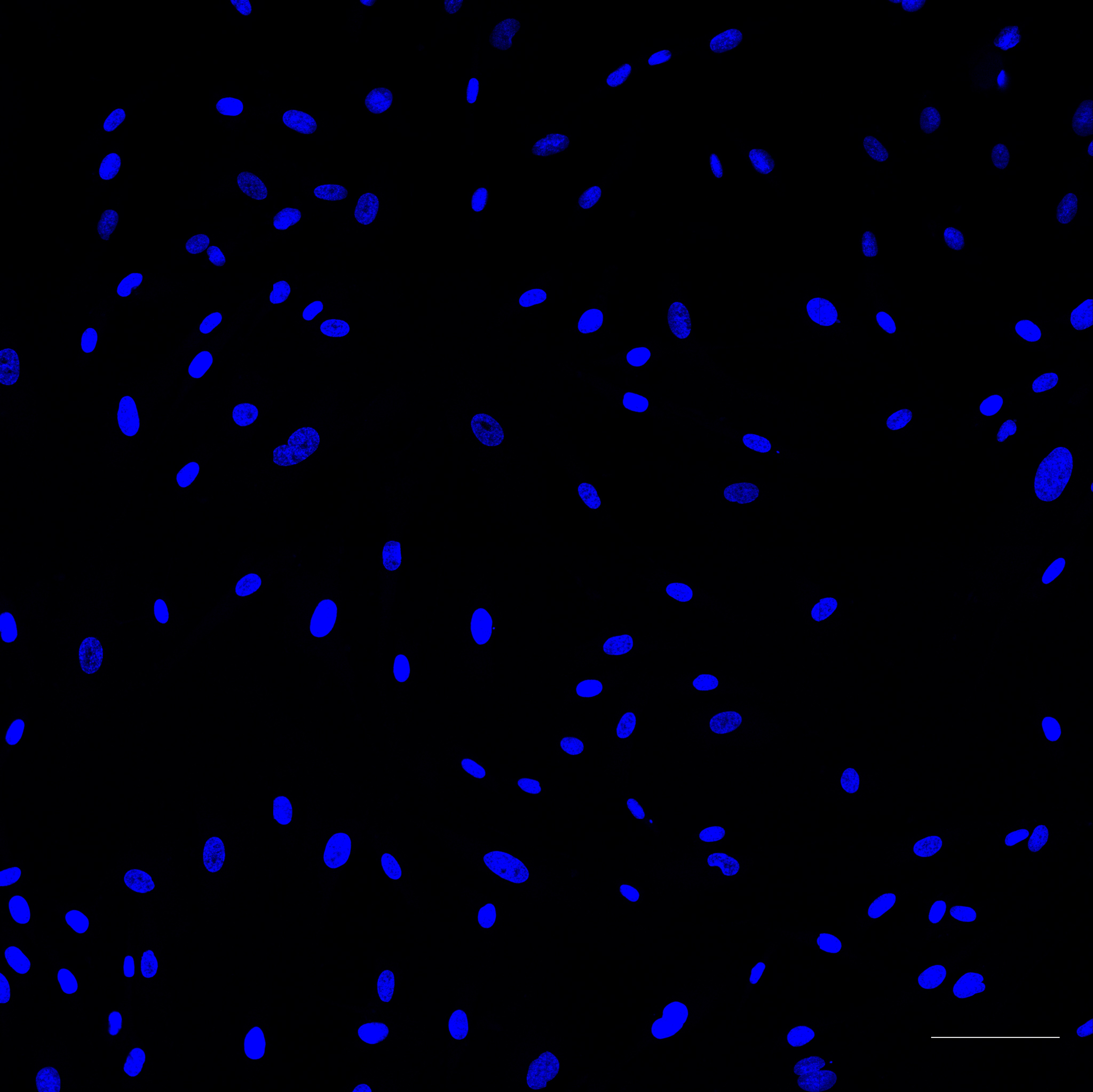

Supplement: Supplementary file 8 — EV and Appendix Figure Source Data [file 44318_2024_163_MOESM8_ESM.zip › Source Data for Expanded View and Appendix/EV4/4A/Microscopy Auxin DAPI.jpg]

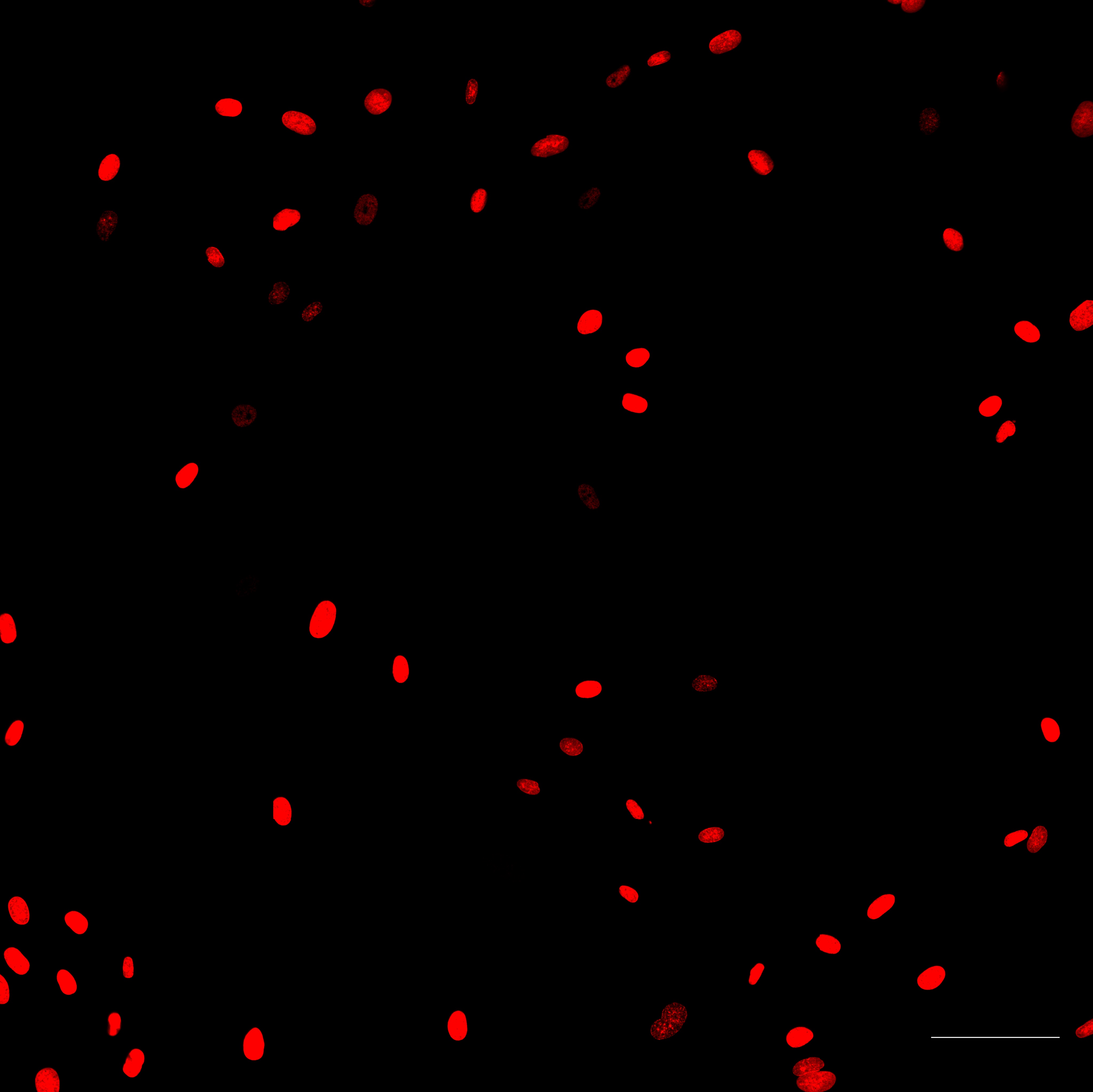

Supplement: Supplementary file 8 — EV and Appendix Figure Source Data [file 44318_2024_163_MOESM8_ESM.zip › Source Data for Expanded View and Appendix/EV4/4A/Microscopy Auxin Edu.jpg]

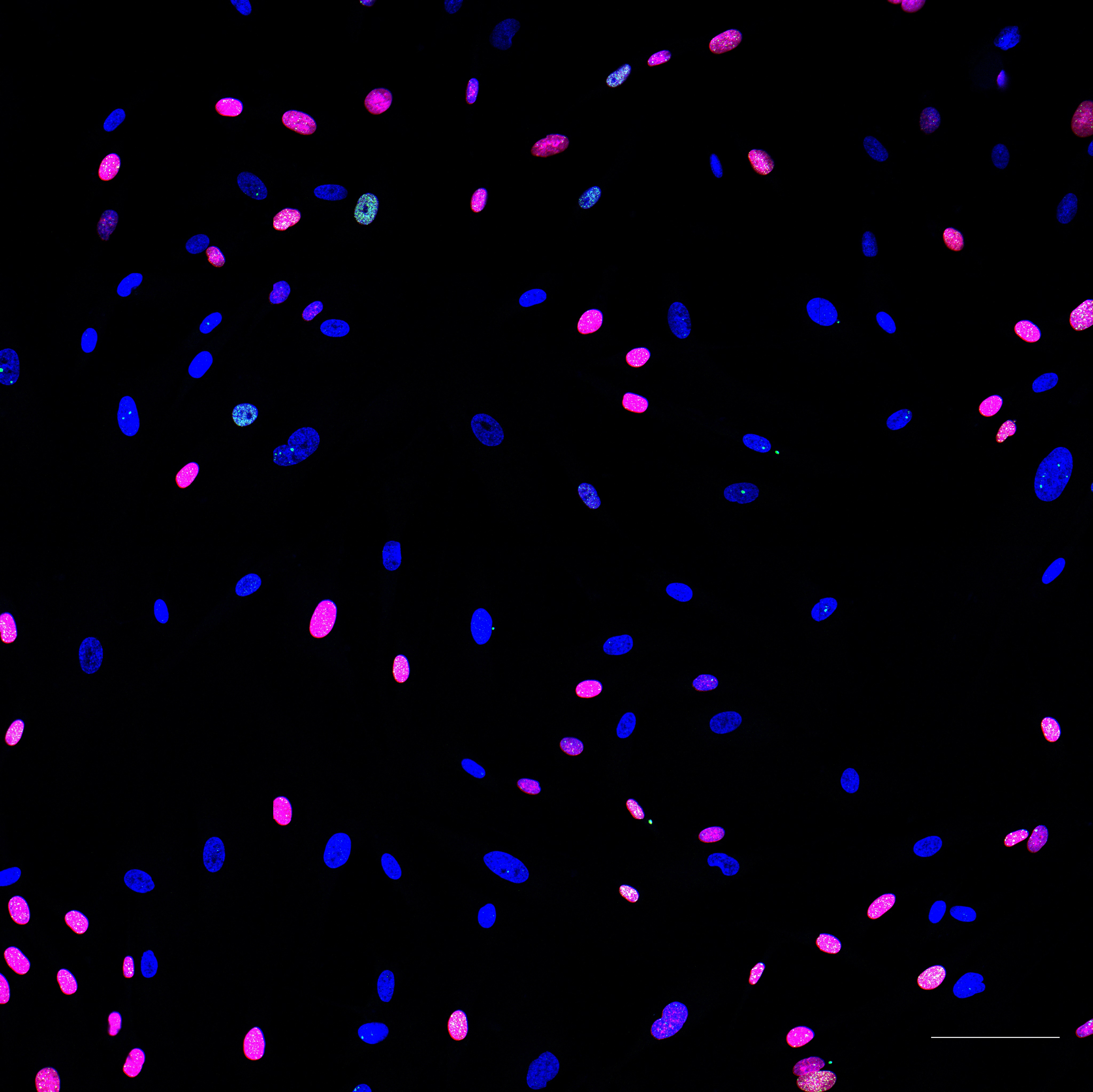

Supplement: Supplementary file 8 — EV and Appendix Figure Source Data [file 44318_2024_163_MOESM8_ESM.zip › Source Data for Expanded View and Appendix/EV4/4A/Microscopy Auxin merged.jpg]

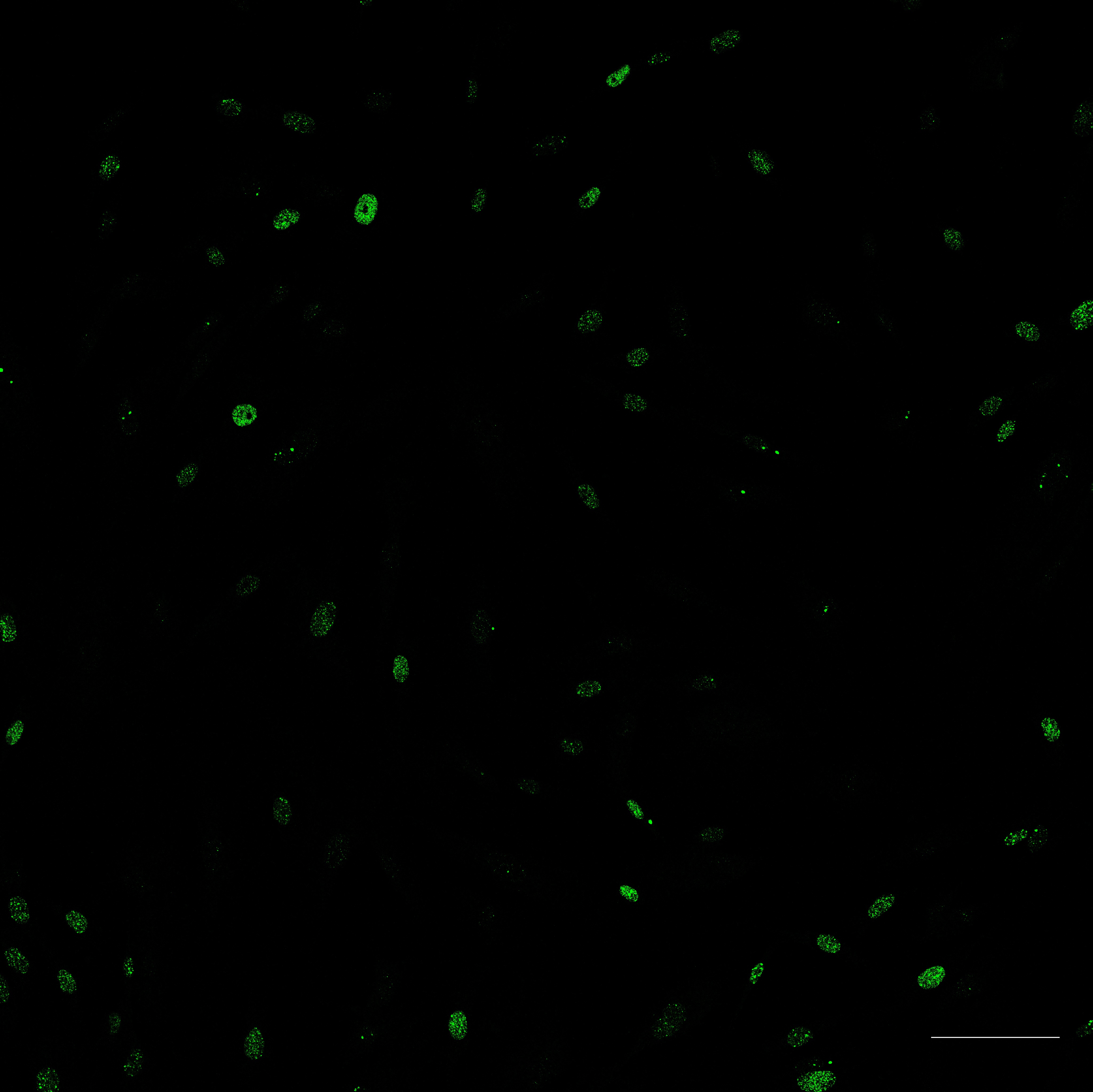

Supplement: Supplementary file 8 — EV and Appendix Figure Source Data [file 44318_2024_163_MOESM8_ESM.zip › Source Data for Expanded View and Appendix/EV4/4A/Microscopy Auxin yH2AX.jpg]

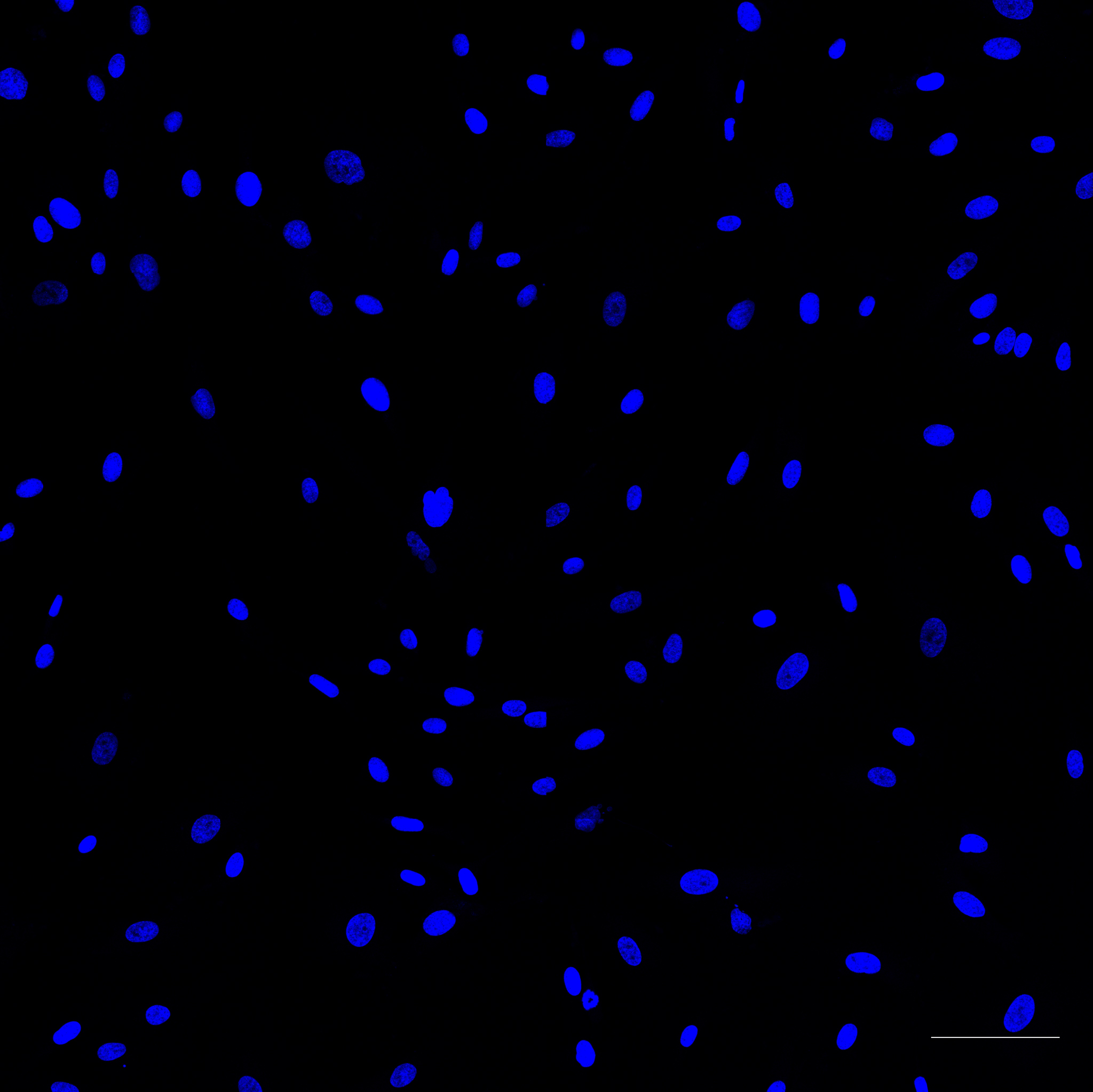

Supplement: Supplementary file 8 — EV and Appendix Figure Source Data [file 44318_2024_163_MOESM8_ESM.zip › Source Data for Expanded View and Appendix/EV4/4A/Microscopy DMSO DAPI.jpg]

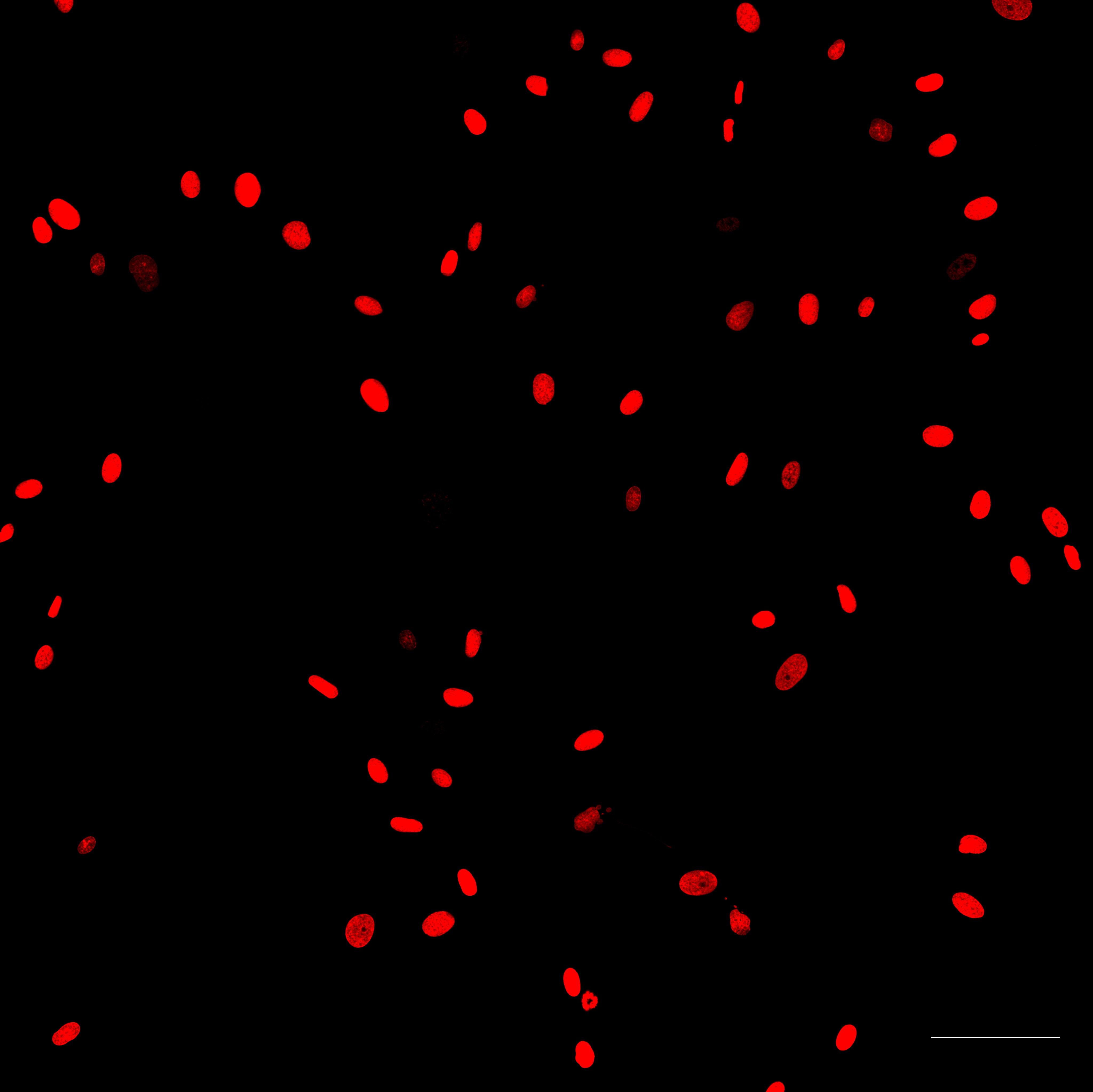

Supplement: Supplementary file 8 — EV and Appendix Figure Source Data [file 44318_2024_163_MOESM8_ESM.zip › Source Data for Expanded View and Appendix/EV4/4A/Microscopy DMSO Edu.jpg]

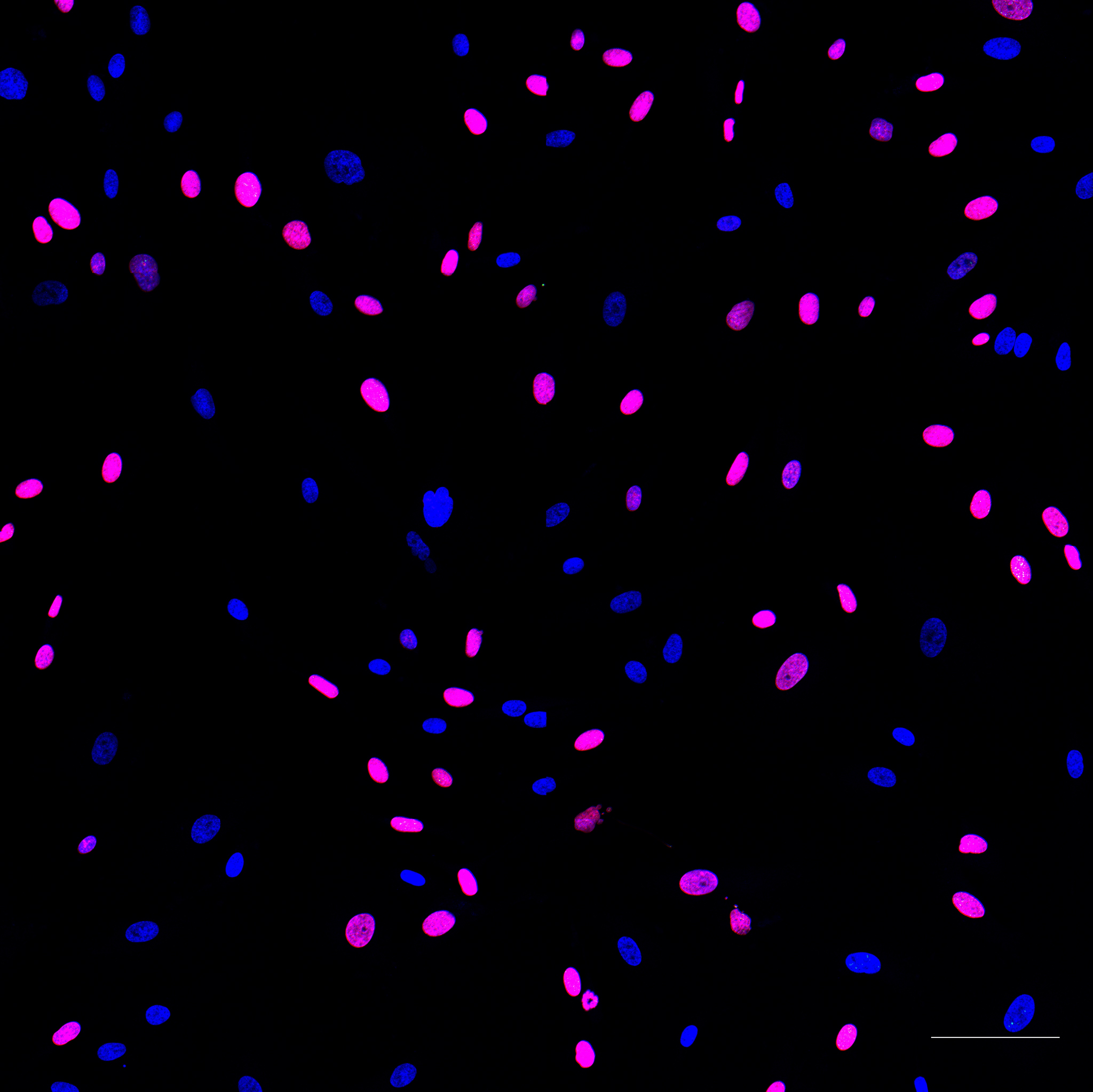

Supplement: Supplementary file 8 — EV and Appendix Figure Source Data [file 44318_2024_163_MOESM8_ESM.zip › Source Data for Expanded View and Appendix/EV4/4A/Microscopy DMSO merged.jpg]

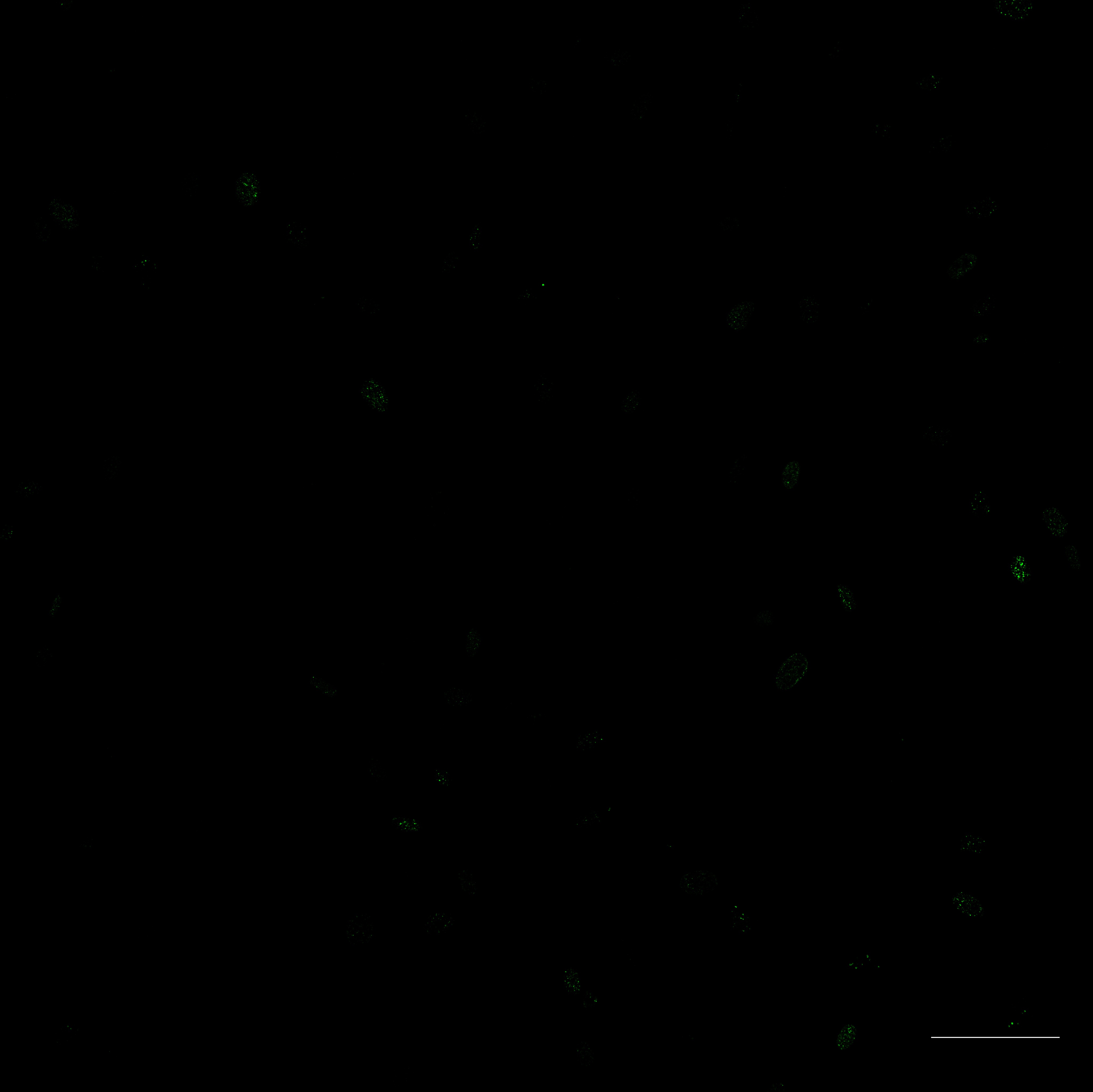

Supplement: Supplementary file 8 — EV and Appendix Figure Source Data [file 44318_2024_163_MOESM8_ESM.zip › Source Data for Expanded View and Appendix/EV4/4A/Microscopy DMSO yH2AX.jpg]

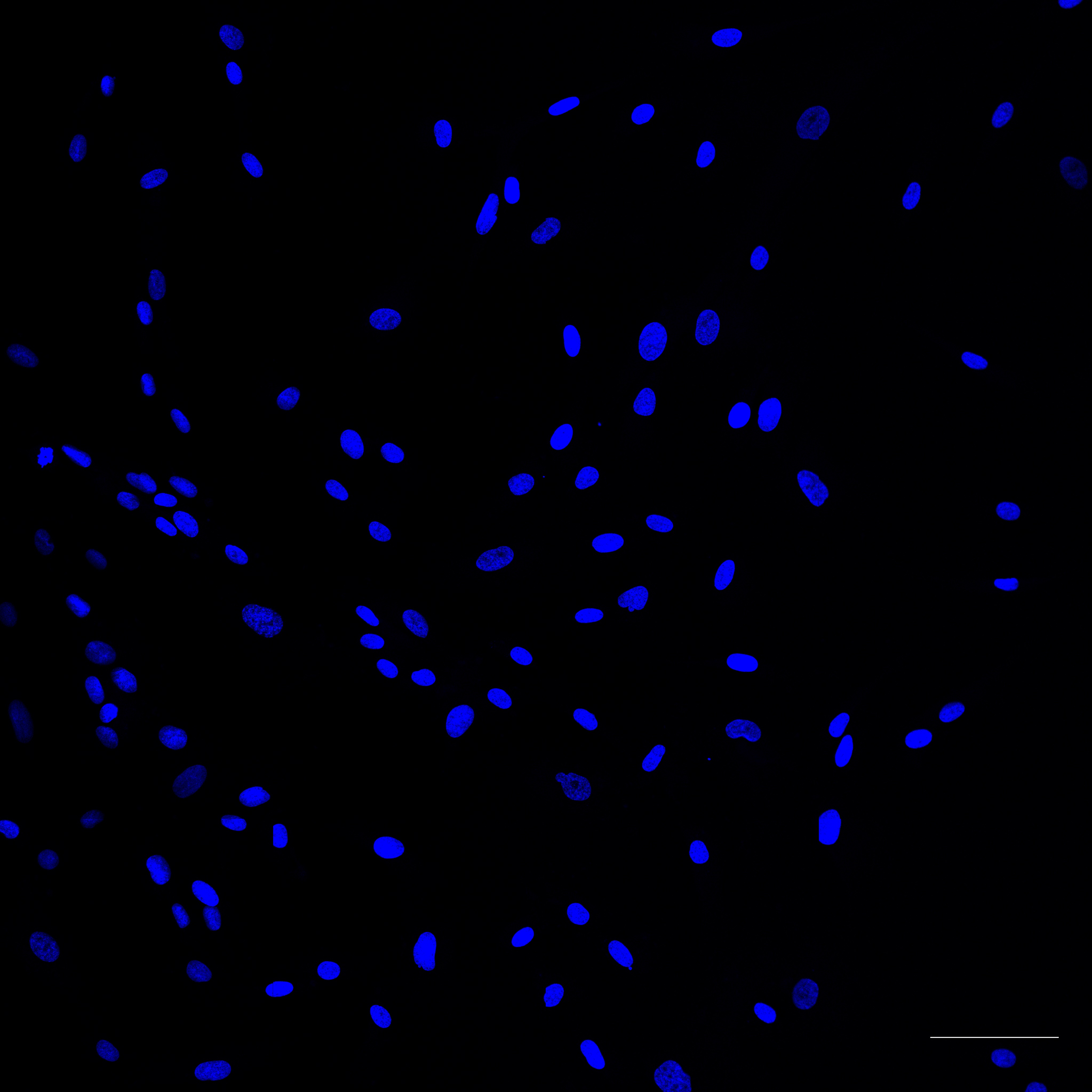

Supplement: Supplementary file 8 — EV and Appendix Figure Source Data [file 44318_2024_163_MOESM8_ESM.zip › Source Data for Expanded View and Appendix/EV4/4A/Microscopy NM DAPI.jpg]

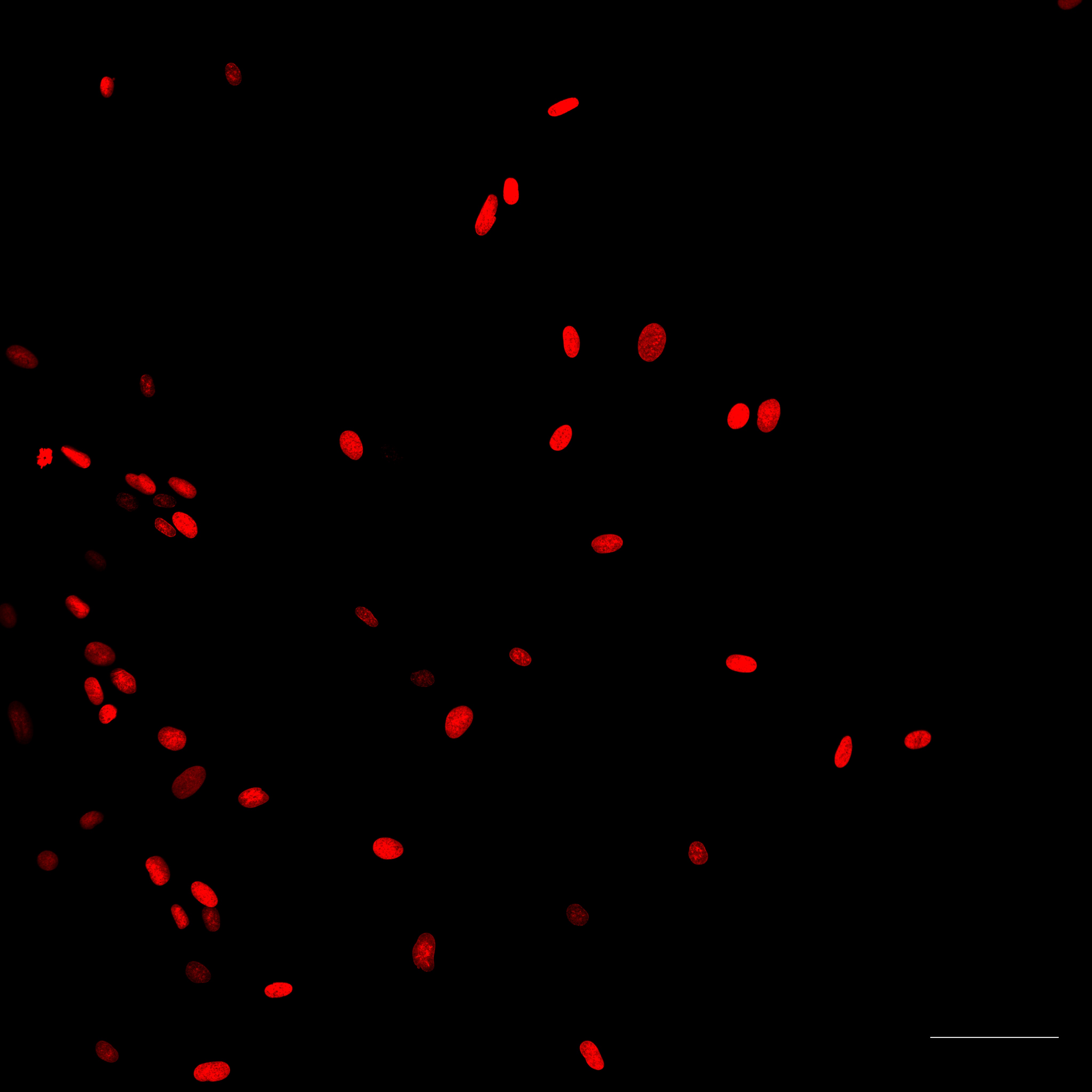

Supplement: Supplementary file 8 — EV and Appendix Figure Source Data [file 44318_2024_163_MOESM8_ESM.zip › Source Data for Expanded View and Appendix/EV4/4A/Microscopy NM Edu.jpg]

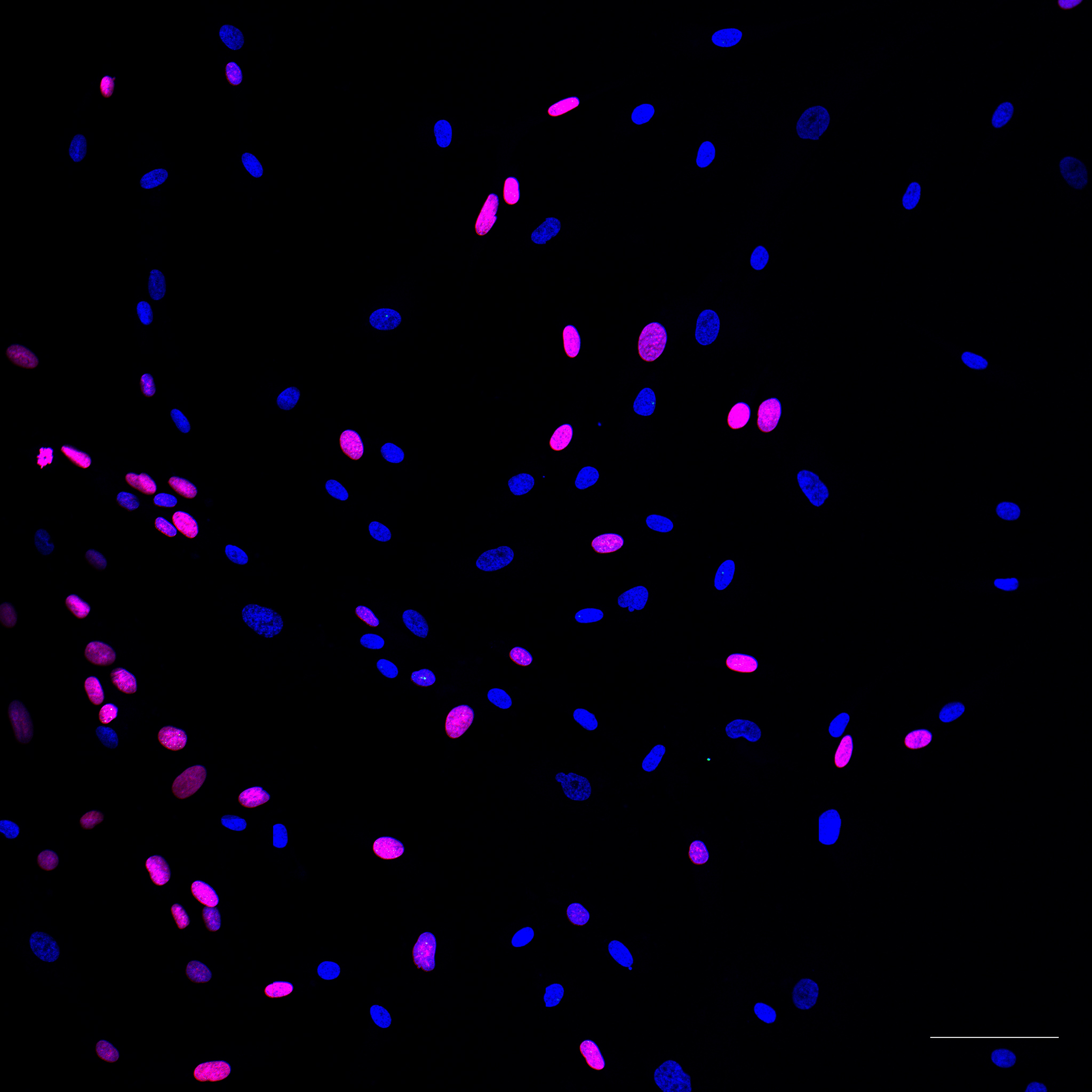

Supplement: Supplementary file 8 — EV and Appendix Figure Source Data [file 44318_2024_163_MOESM8_ESM.zip › Source Data for Expanded View and Appendix/EV4/4A/Microscopy NM merged.jpg]

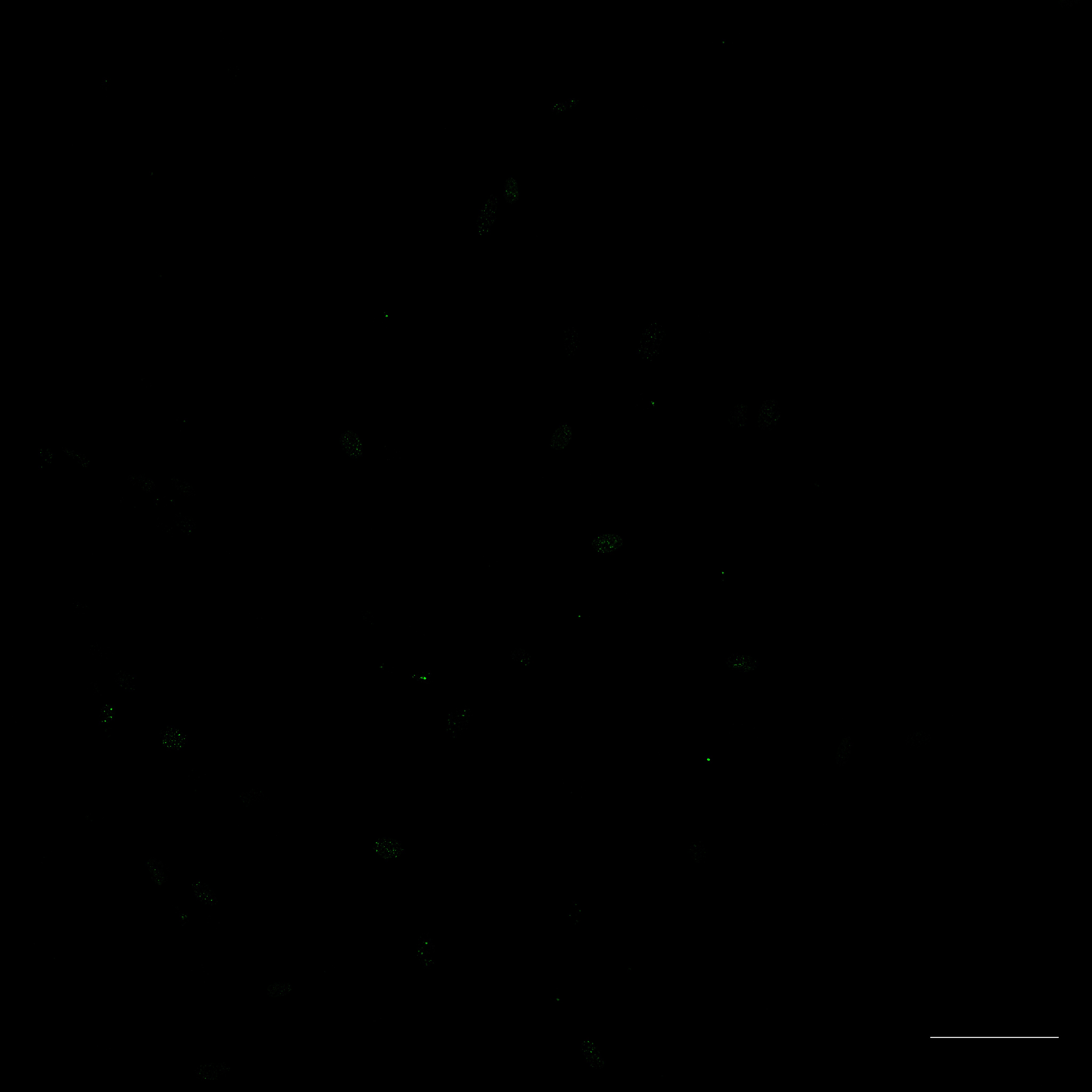

Supplement: Supplementary file 8 — EV and Appendix Figure Source Data [file 44318_2024_163_MOESM8_ESM.zip › Source Data for Expanded View and Appendix/EV4/4A/Microscopy NM yH2AX.jpg]

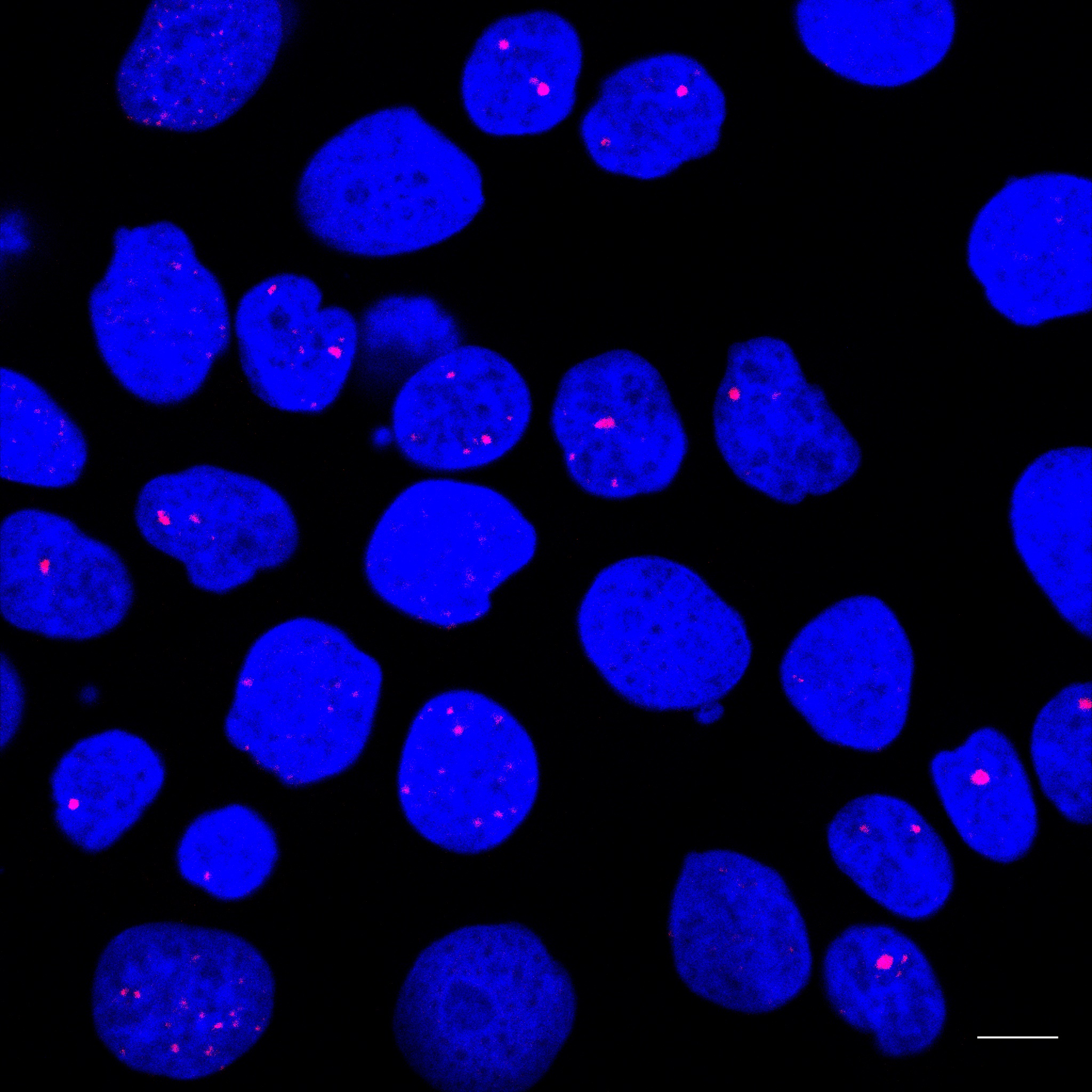

Supplement: Supplementary file 8 — EV and Appendix Figure Source Data [file 44318_2024_163_MOESM8_ESM.zip › Source Data for Expanded View and Appendix/EV4/4B/Microscopy Auxin 53BP1 DAPI.jpg]

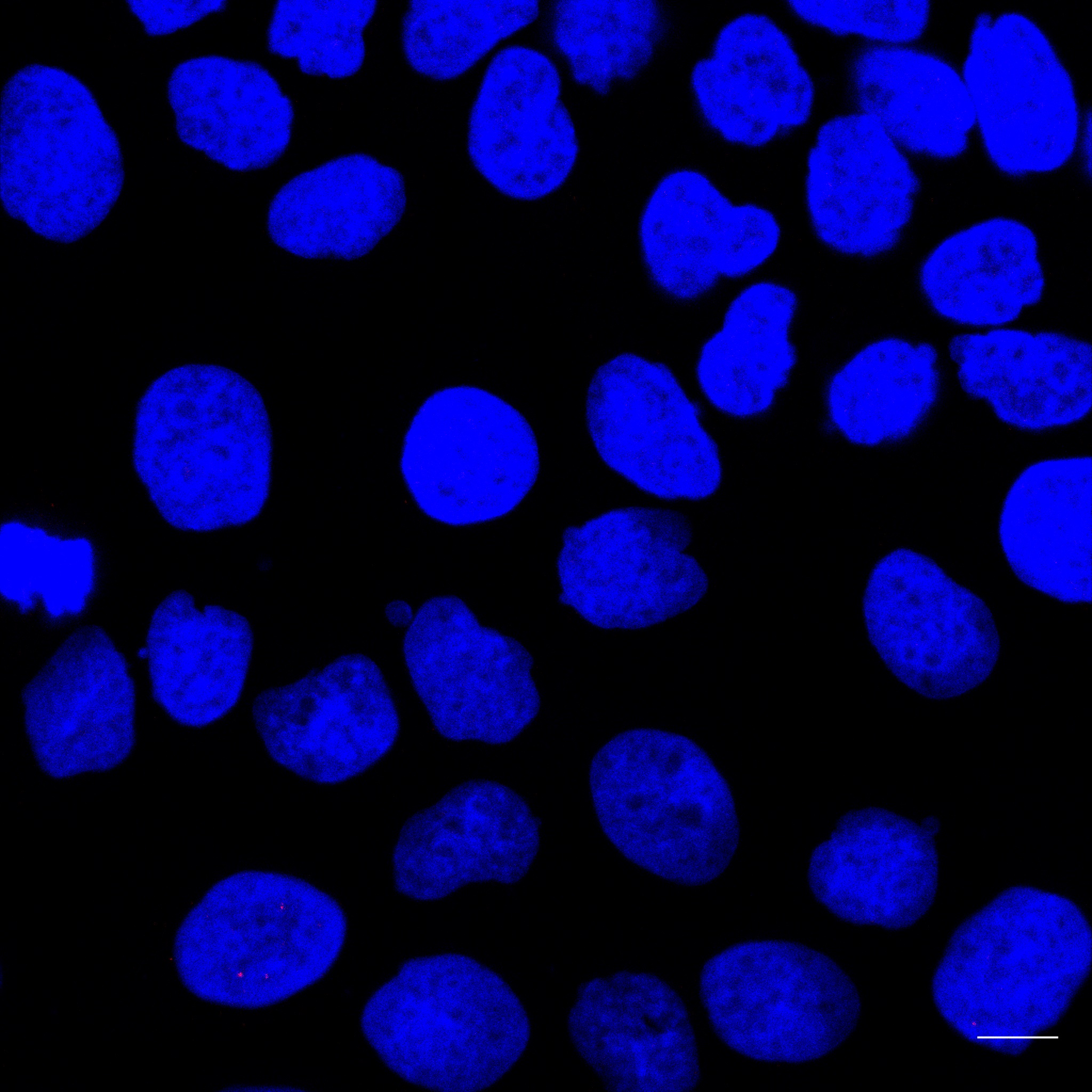

Supplement: Supplementary file 8 — EV and Appendix Figure Source Data [file 44318_2024_163_MOESM8_ESM.zip › Source Data for Expanded View and Appendix/EV4/4B/Microscopy DMSO 53BP1 DAPI.jpg]

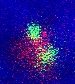

Supplement: Supplementary file 8 — EV and Appendix Figure Source Data [file 44318_2024_163_MOESM8_ESM.zip › Source Data for Expanded View and Appendix/EV4/4C/Inset 1.jpg]

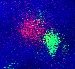

Supplement: Supplementary file 8 — EV and Appendix Figure Source Data [file 44318_2024_163_MOESM8_ESM.zip › Source Data for Expanded View and Appendix/EV4/4C/Inset 2.jpg]

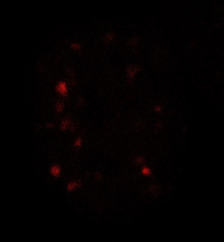

Supplement: Supplementary file 8 — EV and Appendix Figure Source Data [file 44318_2024_163_MOESM8_ESM.zip › Source Data for Expanded View and Appendix/EV4/4C/Microscopy 53BP1.tif]

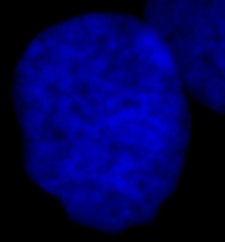

Supplement: Supplementary file 8 — EV and Appendix Figure Source Data [file 44318_2024_163_MOESM8_ESM.zip › Source Data for Expanded View and Appendix/EV4/4C/Microscopy DAPI.tif]

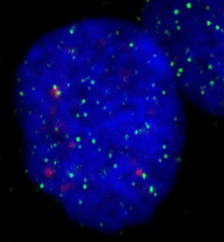

Supplement: Supplementary file 8 — EV and Appendix Figure Source Data [file 44318_2024_163_MOESM8_ESM.zip › Source Data for Expanded View and Appendix/EV4/4C/Microscopy merged.tif]

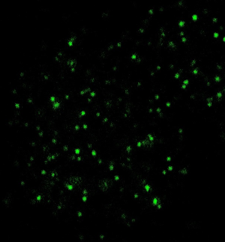

Supplement: Supplementary file 8 — EV and Appendix Figure Source Data [file 44318_2024_163_MOESM8_ESM.zip › Source Data for Expanded View and Appendix/EV4/4C/Microscopy Telomere-Cy3.tif]

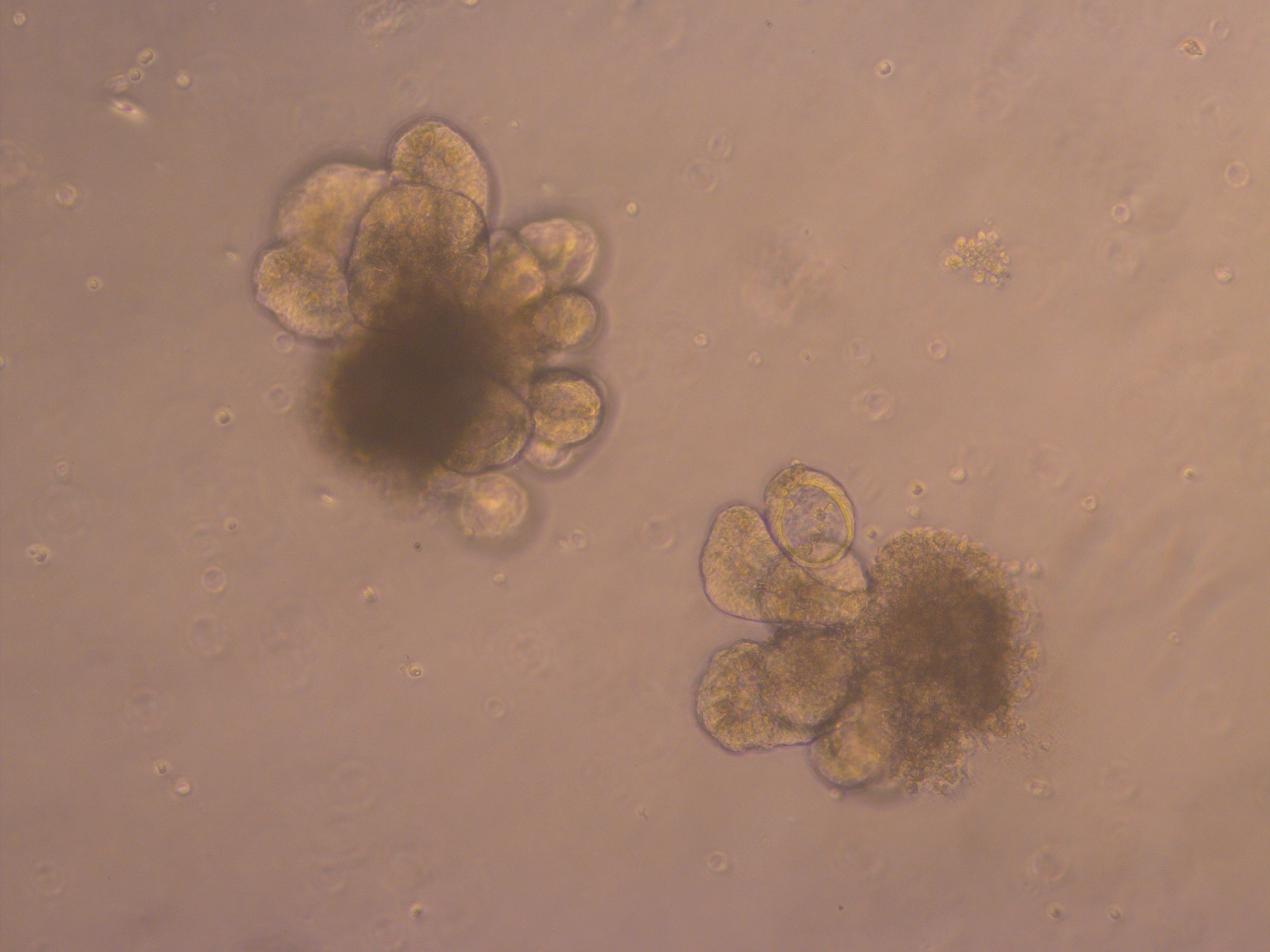

Supplement: Supplementary file 8 — EV and Appendix Figure Source Data [file 44318_2024_163_MOESM8_ESM.zip › Source Data for Expanded View and Appendix/EV5/5A/Microscopy organoids CAD KO.jpg]

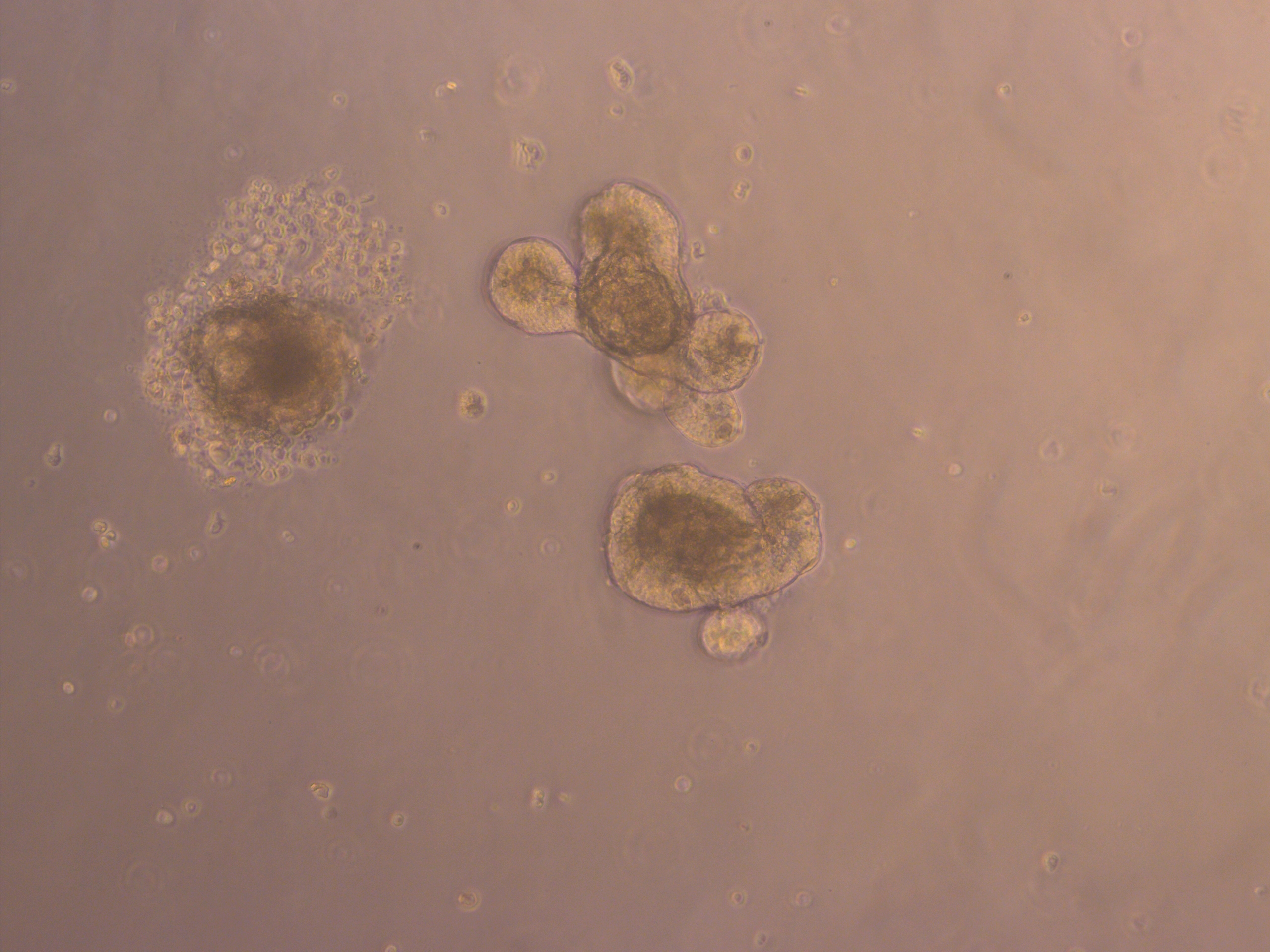

Supplement: Supplementary file 8 — EV and Appendix Figure Source Data [file 44318_2024_163_MOESM8_ESM.zip › Source Data for Expanded View and Appendix/EV5/5A/Microscopy organoids wt.jpg]

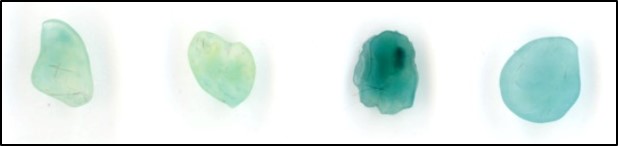

Supplement: Supplementary file 8 — EV and Appendix Figure Source Data [file 44318_2024_163_MOESM8_ESM.zip › Source Data for Expanded View and Appendix/EV5/5D/betaGal WAT.jpg]
