# Supplementary material for: The caspase-activated DNase promotes cellular senescence
Source: EMBO J. 2024 Jul 8;43(16):11. doi: 10.1038/s44318-024-00163-9 (PMC11329656; doi:10.1038/s44318-024-00163-9)
Supplement: Supplementary file 9 — Expanded View Figures [file 44318_2024_163_MOESM9_ESM.pdf]

Expanded View Figures

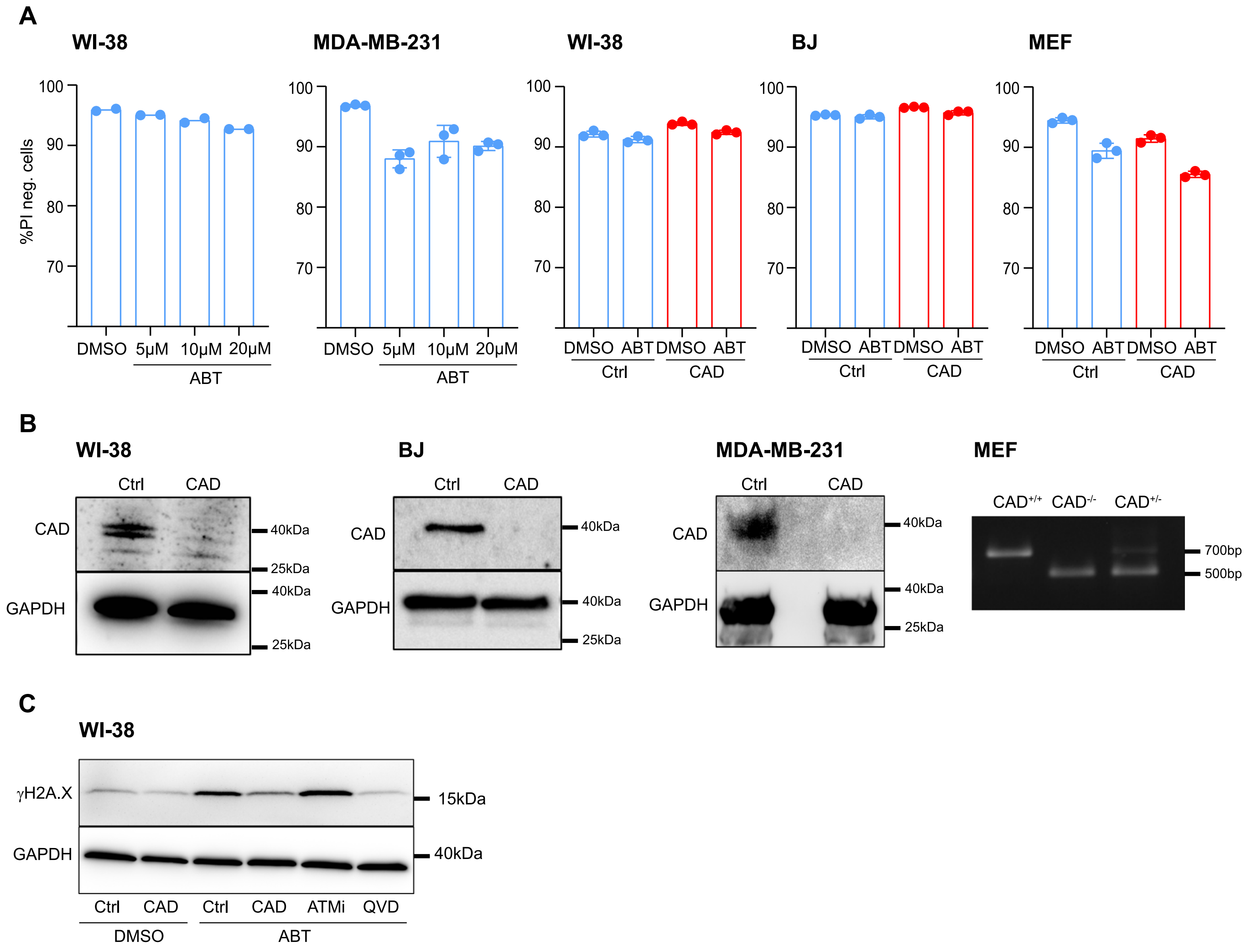

Figure EV1. Sub-lethal CAD-activation.

(**A**) Percentage of viable cells after ABT treatment (48 h) were measured by flow cytometry (PI staining). For the experiments where CAD-deficient cells are used, cells were treated with 5 μM ABT. Each symbol shows the result from one independent experiment. Data represent the mean/SD. (**B**) Confirmation of CAD-deletion was measured by western blot. GAPDH is used as loading control. For the MEF, genotyping for CAD is shown. (**C**) WI-38 cells were treated with 5 μM ABT-737 for 48 h, in the presence or not of QVD (20 μM) or the ATM inhibitor Ku55933 (10 μM). γH2A.X protein expression was analyzed by western blot. GAPDH is used as loading control. Blot is representative of 3 independent experiments. Source data are available online for this figure.

 © The Author(s)

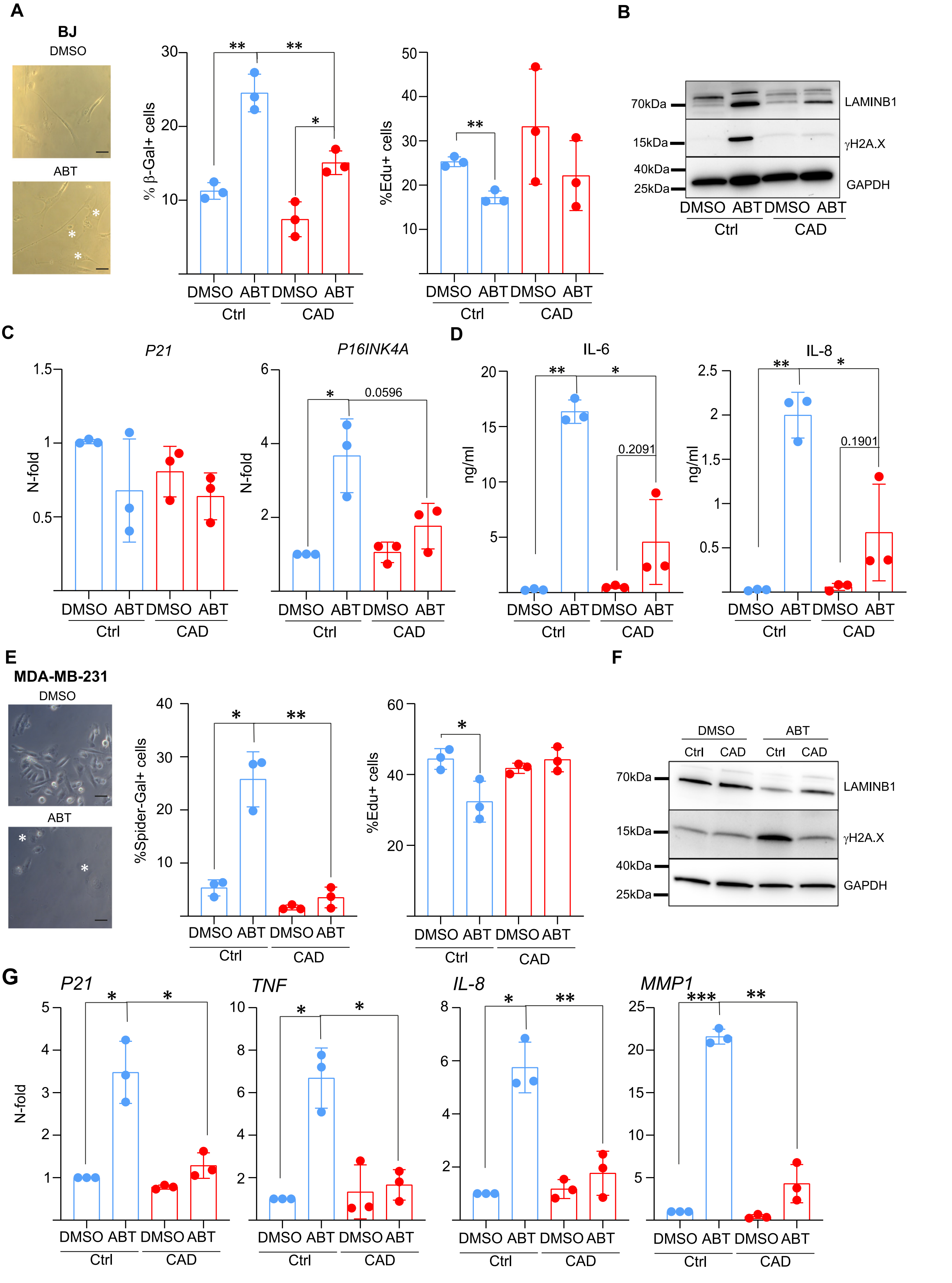
A
BJ
DMSO
ABT
% β-Gal+ cells
%Edu+ cells
DMSO ABT DMSO ABT
Ctrl CAD
B
70kDa
15kDa
40kDa
25kDa
LAMINB1
γH2A.X
GAPDH
C
P21
P16INK4A
N-fold
0.0596
D
IL-6
IL-8
ng/ml
0.2091
0.1901
E
MDA-MB-231
%Spider-Gal+ cells
F
G
TNF
IL-8
MMP1

Figure EV2. Sub-lethal activation of CAD can trigger senescence.

(**A**) BJ fibroblasts (carrying a non-targeting gRNA (Ctrl) or CAD-deficient) were treated with 5 μM ABT-737. Media was replaced with fresh ABT every 48 h over a period of 21 days. Bright-field images show cells after 21 days of ABT treatment. White stars highlight cells with enlarge and flat morphology. Scale bar: 50 μm. Cells were stained with β-galactosidase staining solution and Edu and percentages of SA-β-Gal$^+$ and Edu$^+$ cells were quantified by microscopy. (**B**) Cells were treated as in (**A**). Lamin B1 and γH2A.X protein expression was analyzed by western blot. GAPDH was used as loading control. (**C**) Expression of senescence-associated genes was measured by RT-PCR. (**D**) Supernatants from cells cultured for 21 days were analyzed by ELISA for IL-6 and IL-8. (**E**) MDA-MB-231 cells (carrying a non-targeting gRNA (Ctrl) or CAD-deficient) were treated with 10 μM ABT-737 for 24 h, washed and incubated for 7 days in normal media. Bright-field images show cells after 7 days of ABT treatment. Scale bar: 50 μm. White stars highlight cells with enlarge and flat morphology. SA-β-Gal activity was measured using Spider-Gal. Proliferation was measured with Edu incorporation. Percent positive cells are shown. (**F**) Lamin B1 and γH2A.X protein expression was analyzed by western blot. GAPDH was used as loading control. (**G**) Expression of senescence-associated genes was measured by RT-PCR. Each symbol shows the result from one independent experiment. Data represent the mean/SD. Unpaired parametric *t* test (with Welch's correction) was used to calculate statistical significance. *$P < 0.05$, **$P < 0.01$, ***$P < 0.001$. Source data are available online for this figure.

© The Author(s)

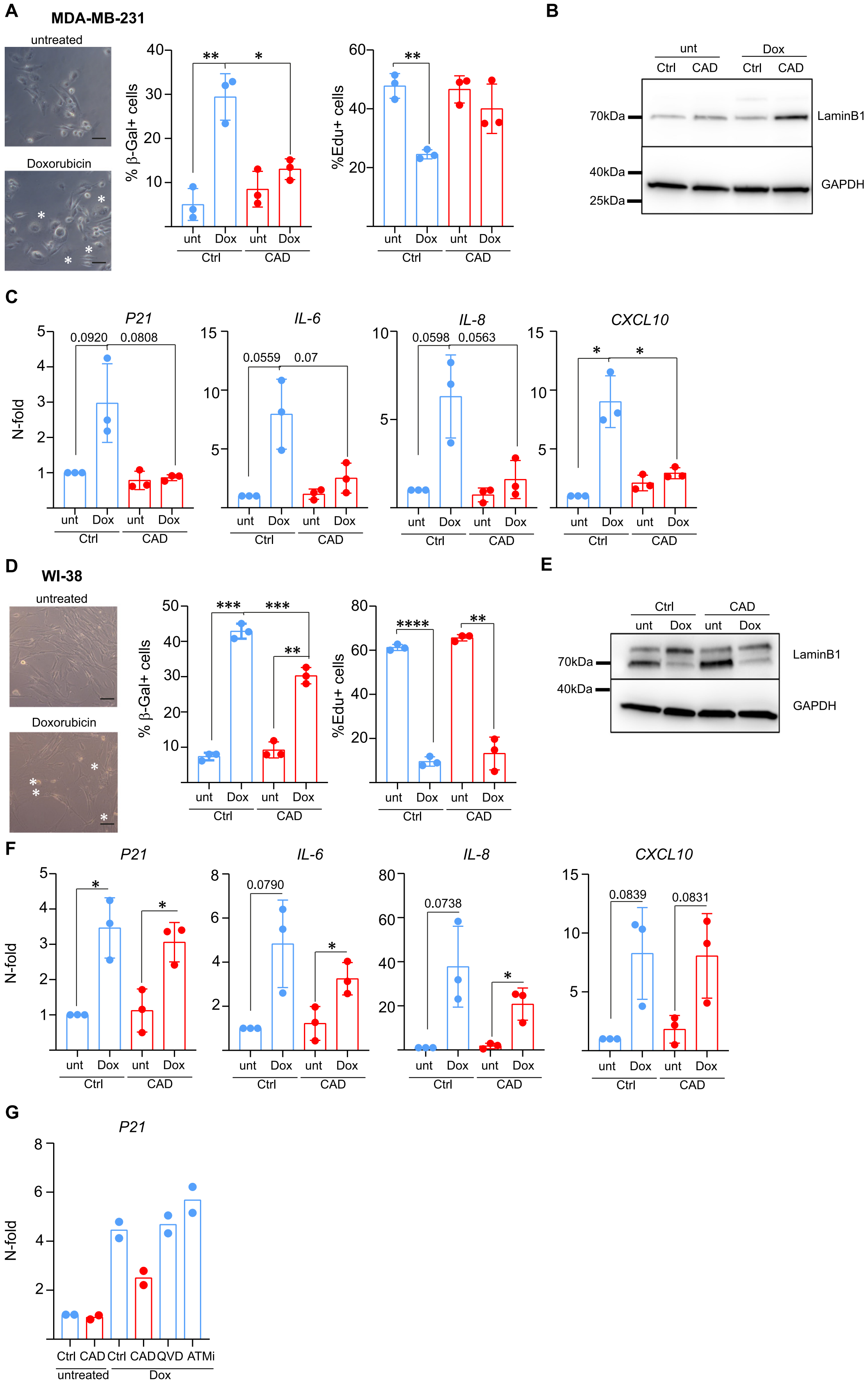
A
MDA-MB-231
untreated
Doxorubicin
% β-Gal+ cells
%Edu+ cells
unt Dox unt Dox
Ctrl CAD
B
unt Dox
Ctrl CAD Ctrl CAD
70kDa
40kDa
25kDa
LaminB1
GAPDH
C
P21
IL-6
IL-8
CXCL10
N-fold
0.0920 0.0808
0.0559 0.07
0.0598 0.0563
D
WI-38
untreated
Doxorubicin
% β-Gal+ cells
%Edu+ cells
E
Ctrl CAD
unt Dox unt Dox
70kDa
40kDa
LaminB1
GAPDH
F
P21
IL-6
IL-8
CXCL10
N-fold
0.0790
0.0738
0.0839
0.0831
G
P21
N-fold
Ctrl CAD Ctrl CAD QVD ATMi
untreated Dox

Figure EV3. CAD-activation may contribute to doxorubicin-induced senescence.

(**A**) MDA-MB-231 cells (carrying a non-targeting gRNA (Ctrl) or CAD-deficient) were treated with 50 nM Doxorubicin for 24 h. Medium was replaced and cells were incubated for 7 days. Bright-field images show cells after 7 days of Doxorubicin treatment. White stars highlight cells with enlarge and flat morphology. Scale bar: 50 μm. Cells were stained with β-galactosidase and Edu staining solution and percentages of SA-β-Gal$^+$ and Edu$^+$ cells were quantified by microscopy. (**B**) Lamin B1 protein expression was analyzed by western blot. GAPDH was used as loading control. (**C**) Expression of senescence-associated genes was measured by RT-PCR. (**D**) WI-38 cells (carrying a non-targeting gRNA (Ctrl) or CAD-deficient) were treated with 250 nM Doxorubicin for 24 h. Medium was replaced and cells were incubated for 7 days. Bright-field images show cells after 7 days of Doxorubicin treatment. White stars highlight cells with enlarge and flat morphology. Scale bar: 50 μm. Cells were stained with β-galactosidase and Edu staining solution and percentages of SA-β-Gal$^+$ and Edu$^+$ cells were quantified by microscopy. (**E**) Lamin B1 protein expression was analyzed by western blot. GAPDH was used as loading control. (**F**) Expression of senescence-associated genes was measured by RT-PCR. (**G**) MDA-MB-231 cells were treated as in (**A**), in presence or absence of QVD or ATM inhibitor. P21 expression was measured by RT-PCR after 7 days of treatment. Each symbol shows the result from one independent experiment. Data represent the mean/SD. Unpaired parametric *t* test (with Welch's correction) was used to calculate statistical significance. *$P < 0.05$, **$P < 0.01$, ***$P < 0.001$, ****$P < 0.0001$. Source data are available online for this figure.

 © The Author(s)

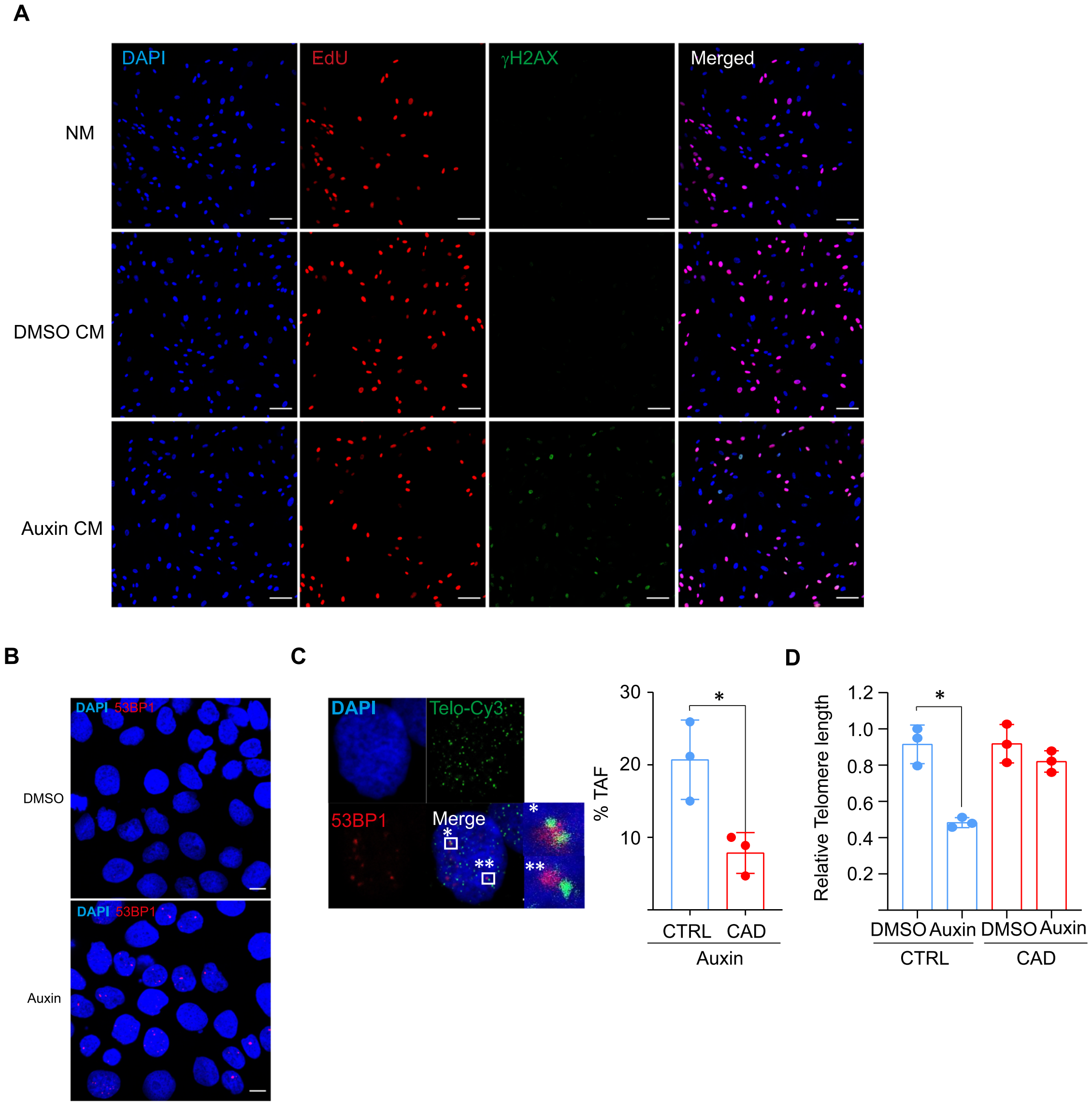

Figure EV4. A CAD-induced DDR at telomeres.

(**A**) Representative fluorescent images of BJ fibroblasts after incubation with normal media (NM), DMSO or Auxin condition media. Cells were stained for Edu, γH2A.X and DAPI. Scale bar: 10 µm. (**B**) HaCaT-ICAD-mAID-GFP cells were treated with auxin for 6 h, fixed and stained for 53BP1 (DDR) with DAPI (DNA). Scale bar: 10 µm. (**C**) HaCaT-ICAD-mAID-GFP cells were treated with auxin for 6 h, fixed and stained for 53BP1 (DDR), for telomeres (using a telomere-specific DNA-probe) and with DAPI (DNA). Images are maximum intensity projections of at least 20 planes. Amplified images on the right (* and **) are from single Z planes where colocalization (*)/close proximity (**) was found. Frequencies of TAF in HaCaT-ICAD-mAID-GFP cells and control cells (HaCaT cells expressing only the Tir1 F-box protein but not the degradable ICAD; see "Methods") upon auxin treatment for 6 h is shown. Results are expressed as percentage of 53PB1 foci that co-localized with the telomere probe. Symbols show results from independent experiments. Data represent the mean/SD. (**D**) Relative telomere length in HaCaT-ICAD-mAID-GFP and HaCaT-ICAD-mAID-GFP CAD-deficient treated with DMSO or auxin for 6 h. Symbols show results from independent experiments ($n = 3$). Data represent the mean/SD. Unpaired parametric *t* test (with Welch's correction) was used to calculate statistical significance. $*P < 0.05$. Source data are available online for this figure.

© The Author(s)

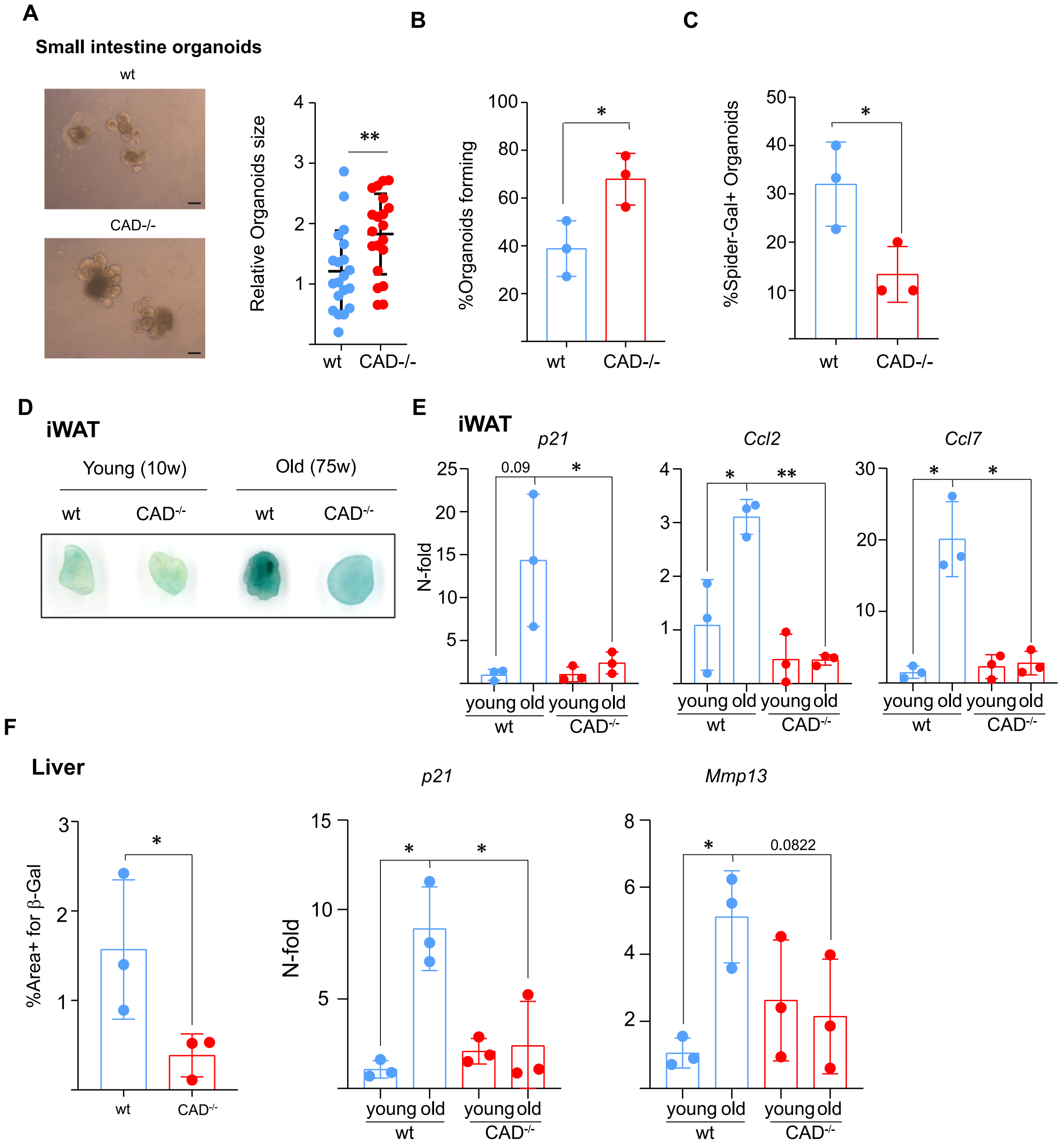

Figure EV5. Small intestinal organoids, adipose tissue and liver were isolated from old (75 weeks) wt and CAD-deficient mice.

(**A**) Exemplary organoids (left) and quantification of organoid size (right) after 5 days in culture are shown. Scale bar: 10 µm. Each symbol shows the size of one organoid. Twenty organoids per group were measured. Data represent the mean/SD. (**B**) Fifty intestinal crypts were seeded per intestine and the percentage of organoids forming after 5 days were quantified. Each symbol represents one mouse/intestine. Data represent the mean/SD. (**C**) Organoids were subjected to staining for SA-β-Gal using Spider-gal. Percentage of organoids staining positive for each mouse are given. Each symbol represents one mouse/intestine ($n = 3$ mice per group). Data represent the mean/SD. Unpaired parametric t test (with Welch's correction) was used to calculate statistical significance. *$P < 0.05$, **$P < 0.01$. (**D**) Inguinal white adipose tissue (iWAT) was isolated from three young and three old mice of each genotype. Tissues were stained for SA-β-Gal activity. Image is representative of 4 stained WAT tissues per group. (**E**) Expression of senescence-associated genes from iWAT samples. Gene expression was measured by RT-PCR. Young (8–10 weeks) ($n = 3$) and old (75 weeks) ($n = 3$) mice per genotype were analyzed. Data represent the mean/SD. (**F**) Liver cryosections from 75-week-old mice were stained for SA-β-Gal activity and the positive area was measured by microscopy (left panel). Each symbol represents one mouse. Expression of senescence-associated genes in liver samples (right panel). Gene expression was measured by RT-PCR. Young (8–10 weeks) ($n = 3$) and old (75 weeks) ($n = 3$) animals were analyzed. Each symbol represents one mouse. Data represent the mean/SD. Unpaired parametric *t* test (with Welch's correction) was used to calculate statistical significance. *$P < 0.05$. Source data are available online for this figure.

 © The Author(s)
